# Hearing Sensitivity of Primates: Recurrent and Episodic Positive Selection in Hair Cells and Stereocilia Protein-Coding Genes

**DOI:** 10.1093/gbe/evab133

**Published:** 2021-06-17

**Authors:** Andreia Moreira, Myriam Croze, Franklin Delehelle, Sylvain Cussat-Blanc, Hervé Luga, Catherine Mollereau, Patricia Balaresque

**Affiliations:** 1 Anthropologie Moléculaire et Imagerie de Synthèse (AMIS), Faculté de Médecine Purpan, CNRS UMR5288, Université de Toulouse, Université Toulouse III Paul Sabatier, France; 2 Institut de Recherche en Informatique de Toulouse (IRIT), CNRS UMR5505, Université Toulouse III Paul Sabatier, France

**Keywords:** inner-ear-expressed genes, positive selection, branch-site test, stereocilia, primates, hearing

## Abstract

The large spectrum of hearing sensitivity observed in primates results from the impact of environmental and behavioral pressures to optimize sound perception and localization. Although evidence of positive selection in auditory genes has been detected in mammals including in Hominoids, selection has never been investigated in other primates. We analyzed 123 genes highly expressed in the inner ear of 27 primate species and tested to what extent positive selection may have shaped these genes in the order Primates tree. We combined both site and branch-site tests to obtain a comprehensive picture of the positively selected genes (PSGs) involved in hearing sensitivity, and drew a detailed description of the most affected branches in the tree. We chose a conservative approach, and thus focused on confounding factors potentially affecting PSG signals (alignment, GC-biased gene conversion, duplications, heterogeneous sequencing qualities). Using site tests, we showed that around 12% of these genes are PSGs, an α selection value consistent with average human genome estimates (10–15%). Using branch-site tests, we showed that the primate tree is heterogeneously affected by positive selection, with the black snub-nosed monkey, the bushbaby, and the orangutan, being the most impacted branches. A large proportion of these genes is inclined to shape hair cells and stereocilia, which are involved in the mechanotransduction process, known to influence frequency perception. Adaptive selection, and more specifically recurrent adaptive evolution, could have acted in parallel on a set of genes (*ADGRV1*, *USH2A*, *PCDH15*, *PTPRQ*, and *ATP8A2*) involved in stereocilia growth and the whole complex of bundle links connecting them, in species across different habitats, including high altitude and nocturnal environments.

## Introduction

Significance

Positive selection signals, known to fix mutations beneficially and shaping certain physiological traits, are revealed by comparing genome sequences of related species. Here, we investigate how positive selection may have shaped auditory genes and focused on inner-ear-expressed genes of primates. We found recurrent and episodic signals of positive selection in the primate tree, and observed a concentration of signals in species living at high altitude and in nocturnal species. Physically, these signals manifest themselves in the alteration of genes expressed in hair cells and stereocilia, which are involved in frequency perception.

Audition constitutes a key gate between organisms and their acoustic environments. Hearing sensitivity varies between placental mammal species, influenced by a species’ body size, middle and inner ear features, and continuous adaptation to their acoustic biotope (Vater et al. 2004; Bernardi et al. 2019; Köppl and Manley 2019). Primates diverged from the rest of the mammals around 67.8 Ma (Springer et al. 2012) and colonized very contrasting acoustic biotopes, from rainforest to savanna. Their adaptation to these new biotopes and associated sound propagation modalities (Brown and Waser 2017) has likely had repercussions on their hearing sensitivity, optimizing sensitivity for their lifestyles (including circadian rhythms, i.e., nocturnal, diurnal, or cathemeral) and for interindividual communication (Ramsier and Rauschecker 2017), in a process similar to what has been reported in subterranean mammal species (Heffner 2004; Charlton et al. 2019). Therefore, the acoustic properties of a habitat may have acted as a direct source of selection (Brown and Waser 2017), similarly to how primate calls and the corresponding hearing frequency sensitivity are driven by natural selection (Ramsier et al. 2012). Although the precise morphology of the ear varies across primate species (Coleman and Colbert 2010), all therian mammal ears have the same three-part structure: an external ear, a middle ear, and a coiled inner ear (cochlea). The external ear modulates sound perception (useful for localizing prey or congener) (Coleman and Ross 2004; Dominy et al. 2004; Fleagle 2013), whereas the middle and inner ear are known to influence perception on a spectral basis, weighing signals according to their frequential components. In therian mammals, the middle and inner ear are thought to have coevolved to improve high-frequency sensitivity (Coleman and Boyer 2012; Manley 2017; Köppl and Manley 2019). The ancestors of extant primates are believed to have been nocturnal, small-sized animals with low interaural distances and short cochlea, features associated with high-frequency sensitivity and similar to those found in small mammals and rodents (Heffner 2004; Cao et al. 2015; Nummela 2017; Charlton et al. 2019). Nowadays, Strepsirrhini and tarsiers exhibit the same characteristics and are also more sensitive to high frequencies (Bernardi et al. 2019). Following the trend, New World monkeys, Platyrrhini, discriminate high frequencies better than their Catarrhini relatives (Old World monkeys and Apes) whose larger external ears and cochlea are more sensitive to lower frequencies (Coleman and Ross 2004; Heffner 2004; Nummela 2017). Besides morphological features, the length of the outer hair cells, housed within the cochlea in the organ of Corti, is also linked to hearing sensitivity (Fettiplace 2017). The length varies not only along the cochlea, but also between species. Species more sensitive to higher frequencies typically exhibit relatively shorter cells and stereocilia, whereas species displaying a lower frequency sensitivity have longer cells (Fettiplace 2017). These differences in cellular morphology may be evolutionarily selected to adapt hearing to specific frequency ranges and improve communication. Moreover, the somatic electromotility of the outer hair cells plays a fundamental role in cochlear amplification (Köppl and Manley 2019), and genes implicated in this function (e.g., *SLC26A5*, a gene encoding for prestin) are found to be under positive selection in some mammalian lineages (Franchini and Elgoyhen 2006; Pisciottano et al. 2019). Due to the diversity in frequency sensitivity observed in the order Primates, we hypothesized that positive selection may have shaped genes expressed in the inner ear. Across the primate tree, and depending on the approach used and the number of species considered, 3% (Van Der Lee et al. 2017) to 13% (Yin et al. 2020) of protein-coding genes were found to present sites under positive selection. They were mainly enriched in proteins of immunity, olfaction, and gustatory and auditory perception (Van Der Lee et al. 2017; Yin et al. 2020). Focusing on the hearing system, evidence of molecular adaptation impacting three genes of the auditory genome (*OTOG*, *TECTA*, *MYO15*) was found in a cross-species comparison focusing on “deafness” genes in 69 mammals, including echolocating species such as bats and cetaceans (Kirwan et al. 2013). However, although natural selection and convergent evolution have been examined in echolocating species (Li et al. 2008; Parker et al. 2013), no specific research has been conducted on selective adaptation in the evolution of primate auditory genes. In this article, we focused on 123 genes highly expressed in the inner ear and 27 primate species to obtain a deep evolutionary insight into hearing adaptation. Using site test analyzes, we assessed whether positive selection has influenced the auditory genes, and more specifically, the fraction expressed in the inner ear where signal amplification occurs. Using branch-site tests, we investigated which branches of the primate tree were the most susceptible to selection regarding this set of genes. We chose a conservative approach, and focused on the potential confounding factors affecting positively selected gene (PSG) signals (namely alignment errors, multiple comparisons tests, genomic duplications, and gBGC). We also investigated and discussed the impact of heterogeneous sequencing quality, and demography when data availability allowed. Our results show that adaptive selection acts on inner-ear-expressed genes, and more specifically on genes coding for stereocilia structures inside hair bundles.

## Results

**Fig. 1.**
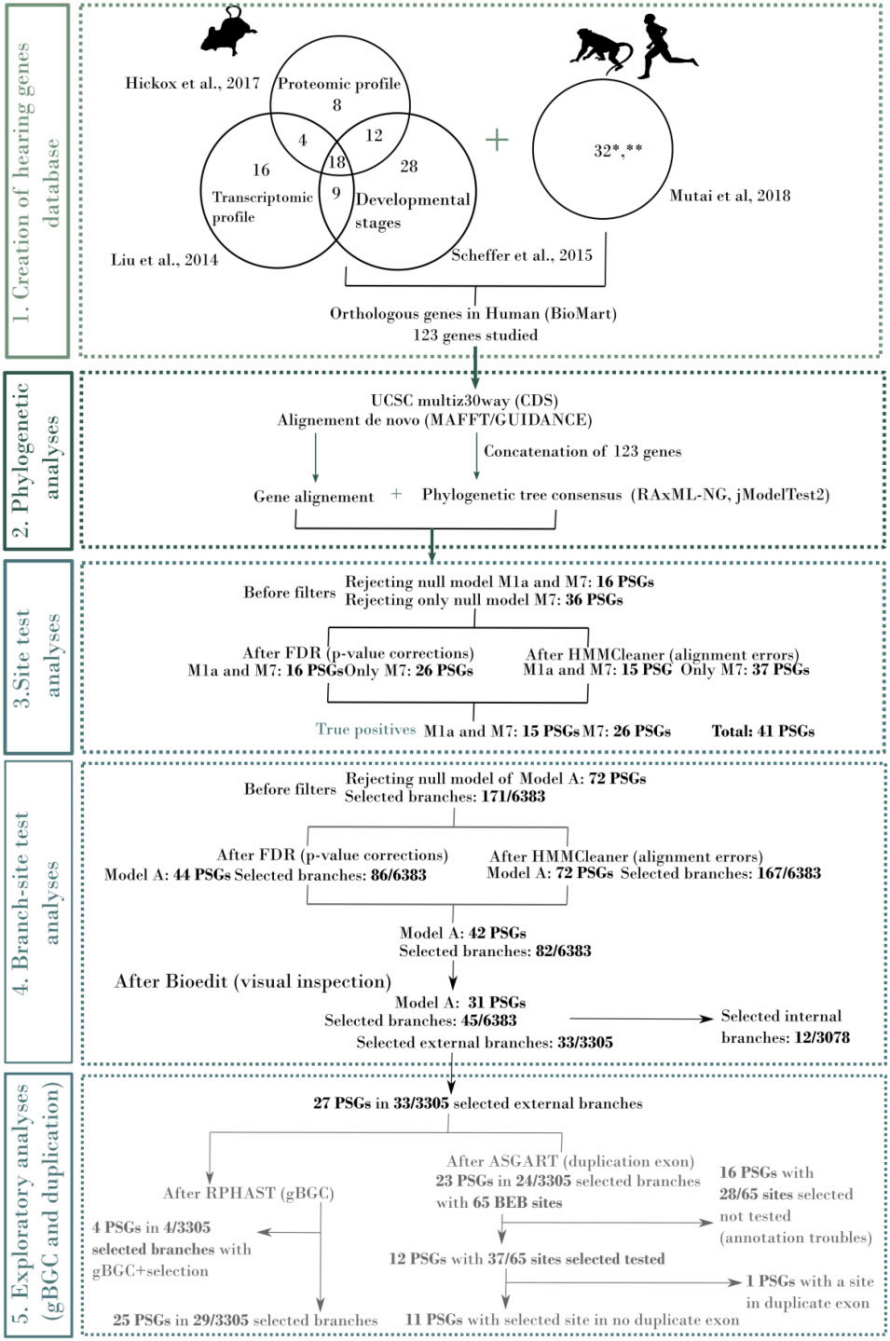
Overview of our comparative evolutionary analysis procedure for conservative inference of positively selected genes and branches. 1) Selection of 123 genes from the EARxpressed repository; 2) multiple sequence alignments and maximum likelihood-based phylogenetic analyzes; 3) site tests (PAML) and confounding factor analyzes (alignment and multiple test corrections); 4) branch-site tests (PAML) and confounding factor analyzes (alignment and multiple test corrections); 5) additional analyzes of complexifying factors (duplications and gBGC).

We analyzed 123 genes expressed in the inner ear of 27 primate species using both site tests, determining selection signals at the base level, and branch-site tests, determining selection signals at the phylogenetic branch level. The five steps of analyzes and results, including evolutionary analyzes and filtering for confounding factors are summarized in figure 1.

### Phylogenetic Analyzes: The 123 Auditory Genes Super-Tree and *ω* Values

The phylogenetic super-tree obtained from the concatenated 319,074 bp of the 123 selected genes is similar to the well-established primate phylogeny (supplementary fig. S34, Supplementary Material online) (Perelman et al. 2011; Springer et al. 2012). The number of nucleotide substitutions per codon ranges from 0.01 to 0.18 across all branches analyzed, in accordance with the whole-genome estimate based on nine primate species (Van Der Lee et al. 2017). *ω* values were calculated for each gene and the median values fluctuate from 0.05 to 0.1 (supplementary table 14, Supplementary Material online) in accordance with Gibbs et al. (2007). The overall *ω* for the whole tree is 0.157 and coherent with purifying selection being the main driving force acting on both primate and mammal genomes as a whole (Mikkelsen et al. 2005; Gibbs et al. 2007; Kosiol et al. 2008; Scally et al. 2012; Van Der Lee et al. 2017).

### Site Test Analyzes and Global Estimate of Inner-Ear-Expressed PSGs in the Primate Tree

Signals of positive selection were detected on 52/123 genes using site test analyzes, including both the genes rejecting the M1a and M7 null models, as well as those rejecting only the M7 null model (fig. 1). After correcting for alignments and multiple tests, 41 PSGs remained (33% of the initial set), including 15 genes rejecting both the M1a and M7 models, and 26 genes rejecting only the M7 model (supplementary table 6, Supplementary Material online). Among the 41 PSGs detected in the site test analysis, 30 were positive to Bayes Empirical Bayes (supplementary table 6, Supplementary Material online). In total, 68 out of 319,074 sites tested were found to be under positive selection (0.021%).

### Branch-Site Test Analyzes: Are Specific Branches Affected by Episodic Positive Selection?

**Fig. 2.**
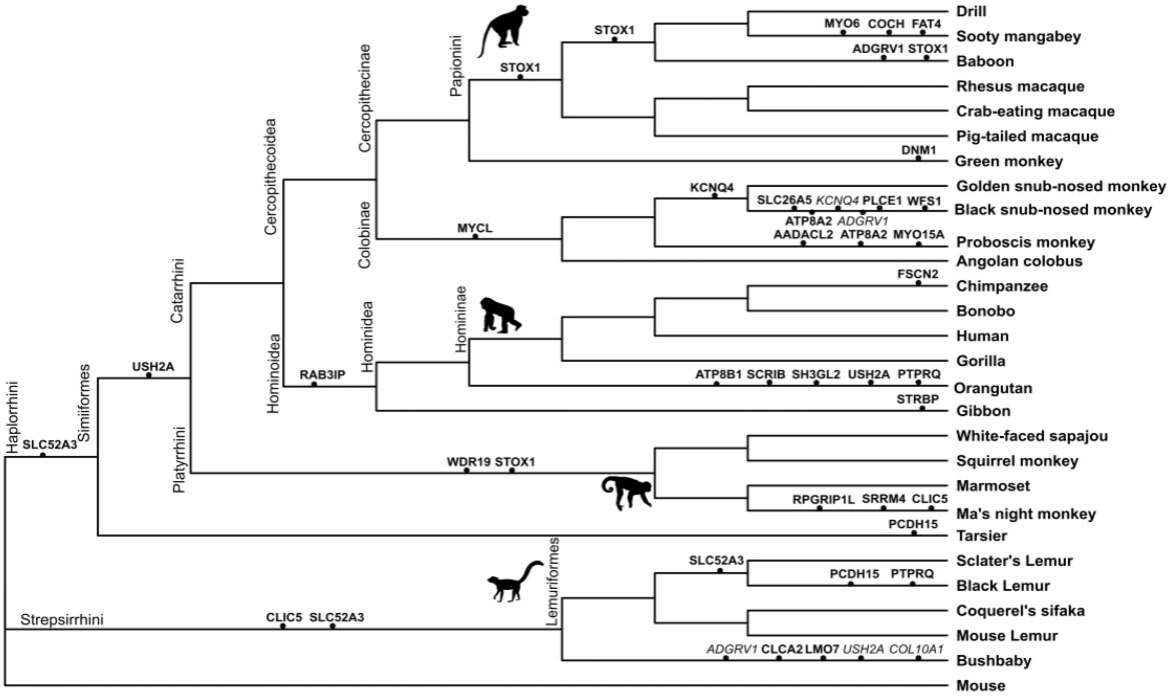
Cladogram of primate species displaying the 31 validated PSGs of branch-site test analyzes after multiple tests plus alignment corrections and a final visual inspection. PSGs potentially impacted by duplications and gBGC are mentioned in italics. Branch lengths are unscaled.

The branch-site test determined which branches of the primate tree were affected by episodic or continuous positive selection. A total of 72 PSGs were detected (fig. 1 and supplementary table 15, Supplementary Material online). After correction for alignments and multiple comparison issues, 42 PSGs remained (supplementary table 16, Supplementary Material online). After a final visual inspection, performed using the Bioedit tool (Hall 1999), 31 PSGs were considered as being true signals (i.e., 25% of the initial gene set, protein alignment in supplementary figs. 1–31, Supplementary Material online) and were placed on the cladogram of primate species (fig. 2). These 31 PSGs represent 45 selective signatures distributed on 12 distinct internal branches and 33 distinct terminal branches. From the 6,383 branches analyzed, 45 showed evidence of positive selection, resulting in a total of 0.70% impacted branches. These 31 validated PSGs are heterogeneously distributed in the whole tree, and are detected preferentially in the black snub-nosed monkey (six PSGs), the bushbaby (five PSGs), and the orangutan (five PSGs). Some subfamilies and families such as Homininae, Hylobatidae, and Cercopithecoidea are almost unaffected (fig. 2). We analyzed the detailed functional enrichment for these 31 genes and found they were mainly annotated with GO terms related to stereocilium organization and neuron projection, suggesting structural changes in hair cells and more specifically in stereocilium structure. Branch-site test analyzes identified the branches displaying the strongest signals, and also the occurrence and temporal scheme of these selective signals. We defined three types of PSGs, the “One-time PSG,” the “Recurrent-PSG,” and the “Continuous-PSG.” Out of the 31 validated PSGs, most were detected only once in the whole primate tree and defined as “One-time PSG” (20/31 PSGs: 65% of all PSG detected) suggesting either the punctual nature of this signal or the lack of genomic information in adjacent branches to make them detectable on deeper branches. Five “Recurrent-PSGs” were detected on the whole tree: *ADGRV1* (Baboon, bushbaby, black snub-nosed monkey), *USH2A* (Bushbaby, orangutan), *PCDH15* (Black lemur and tarsier), *PTPRQ* (Black lemur and orangutan), and *ATP8A2* (Black snub-nosed monkey and proboscis monkey). These five genes code for membrane proteins constitutive of the hair bundles, and more specifically of ankle links (*ADGRV1*, *USH2A*), the tip and kinocilial links (*PCDH15*) and shaft connectors (*PTPRQ*). Nucleotide changes observed affecting the same gene in several lineages might suggest recurrent pressures on this specific cellular component. In the case of *ADGRV1* and *PTPRQ*, the amino acid changes are predicted to have a functional impact (Polyphen2, supplementary table 7, Supplementary Material online). They are located in the extracellular domain of the proteins involved in the links. For *PCDH15*, the site under selection is located in a highly variable intracellular domain, although it is predicted to be potentially damaging. Finally, two genes *STOX1* and *KCNQ4* were identified as “Continuous PSGs” in the Papionini/baboon respectively, snub-nosed monkey (genus *Rhinopithecus*)/black snub-nosed monkey. The gene *STOX1* is involved in regulating the proliferation of inner ear epithelial cells (Chen et al. 2019) and *KCNQ4* is a voltage-gated potassium channel that plays an essential role in maintaining ion homeostasis and regulating hair cell membrane potential. Interestingly, two of the amino acid changes in *KCNQ4* correspond to human polymorphisms, and could suggest potential balancing selection acting in humans (supplementary table 7, Supplementary Material online). To complete the branch-site analyzes, we explored the potential impact of the duplications and the biased gene conversion (gBGC) on the remaining 31 PSGs. Out of these 31 PSGs, 27 PSGs correspond to 33 external branches that could be investigated. Using RPHAST, we showed that gBGC could have affected four genes (*ADGRV1*, *COL10A1*, *USH2A*, and *KCNQ4*) out of the 27 PSGs, specifically in the bushbaby and the black snub-nosed monkey (supplementary table S10, Supplementary Material online). However, the probability of detecting gBGC is systematically associated with the same probability of detecting either positive selection (e.g., *KCNQ4* for the black snub-nosed monkey and *COL10A1* for the bushbaby) or relaxation of purifying selection (e.g., *ADGRV1* and *USH2A* for the bushbaby). None of the signals detected display a unique probability of detecting gBGC. In total, 25 PSGs could still be considered as true PSGs after filtering for gBGC (supplementary table 10, Supplementary Material online). We explored the potential impact of duplication by analyzing only the PSGs associated with identified BEB sites: 23 PSGs, corresponding to 65 identified BEB sites in external branches were tested. Due to partially available genomic information for some species, the analyzes were restricted to 12 PSGs out of these 23, further limiting our conclusions (fig. 2 and supplementary table 8, Supplementary Material online). BEB and SD intersections were tested for 12 PSGs, representing 12 external branches, 17 exons, and an associated 37 BEB sites. Most of the PSGs were considered as true PSGs, and only one duplicated exon intersected with a BEB in *ADGRV1* in the black snub-nosed monkey and was considered as a potentially false PSG (supplementary tables 8 and 9, Supplementary Material online). Although the differences in genome quality, annotations, and SD detection between species could limit our current investigation, at this stage, neither duplication nor gBGC could be considered as a strong confounding factor. More genomic information would be required for these tests to be conclusive.

### Site and Branch-Site Tests: Shared-PSG Signatures between Both Approaches

We used site and branch-site tests in parallel to detect: 1) evidence of adaptive selection on the whole primate phylogeny using site tests; and 2) the most impacted PSG/branch couples using branch-site tests. A total of 35 shared PSGs were revealed by both approaches before filtering for confounding factors which resulted in a remaining 13 PSGs (Venn diagram in supplementary fig. 35, Supplementary Material online). Five of these 13 PSGs have been already reported in the literature as PSGs or accelerated genes involved in auditory function in Homininae (Humans, Chimpanzees, Gorillas) and others mammals *ADGRV1* (Scally et al. 2012; Pisciottano et al. 2019), *CLIC5* (Cao et al. 2015), *MYO15A* (Kirwan et al. 2013), *RPGRIP1L* (Van Der Lee et al. 2017), and *SRRM4* (Lindblad-Toh et al. 2011). Among the filtered PSGs, *CDH23*, *TMC1*, and *OTOF* have also been reported in the literature (Scally et al. 2012) and could have been overfiltered using our procedure. Interestingly, the genes detected in the Homininae branch were not detected before or after filtering for confounding factors; this might be explained by our use of more recent versions of the concerned genomes compared with Scally et al. (2012), removing potentially ambiguous sites or due to the higher number of primate species analyzed here allowing a more accurate detection of adaptive selection (McBee et al. 2015). New PSGs have been revealed by our study including *AADACL2*, *CLCA2*, *WFS1*, *COL10A1*, *LMO7*, *SLC52A3*, and *STOX1*. Two of these genes are involved in cell adhesion: *CLCA2* a calcium activated chloride channel regulator, and *LMO7* described to organize the actin network in the cuticular plate and cell junctions (Du et al. 2019). The splicing *(SRRM4*) and transcription *(STOX1*) factors are involved in gene regulation. *WFS1* is a reticulum endoplasmic cation channel playing a role in Ca^2+^ homeostasis, and is responsible for the hearing disorder Wolfram Syndrome Type l. In the case of the deacetylase *AADACL2* and the collagen *COL10A1*, several sites are found under selection and these nucleotide sites are polymorphic in humans and might suggest that balancing selection could be active at the intraspecific level in humans (supplementary table 7, Supplementary Material online).

### Impact of Sequencing Quality on PSG Detection

We tested the impact of heterogeneous genome quality on PSG detection by comparing a set of metrics with the number of PSGs detected. To be objective and in the absence of NG50 and Q-score metrics for all primates, we chose to run the QUAST-LG tool on raw data to generate the quality metrics (supplementary table 12, Supplementary Material online) (Kuderna et al. 2020; Mikheenko et al. 2018). The relative position of the analyzed genome was assessed using these metrics and a PCA; no significant association between PSG number and genome quality was observed: PCA coordinates were not detected for axis 1 (Kruskal–Wallis test, χ^2^ = 5.15, *P* value <0.08) nor axis 2 (Kruskal–Wallis test, χ^2^ = 1.5 *P* value <0.47). We concluded that the number of PSGs detected is not associated to the relative quality of the genome analyzed.

### Impact of Demographic History on Detected PSGs

The retention of the adaptive allele and the efficacy of selection depend on population genetics and the demographic history of each allele; thus, the large differences in effective population sizes of the primate species analyzed from very large in owl monkeys (Schrago and Seuánez 2019) to relatively low for the genus *Rhinopithecus* (Zhou et al. 2014) might have impacted the heterogeneous fixation of the adaptive alleles. We used the IUCN status as a proxy for demographic history, and found no relation between IUCN status and the number of PSGs detected (Kruskal–Wallis, χ^2^ = 0.88068, df = 4, *P* value = 0.9273). Although we cannot rule out that IUCN status is a poor proxy for current census size and effective population size, IUCN status does provide an approximate index of recent population demography. Further investigations on species-specific demography should be planned, especially on long-term effective population size which should be a more valid indicator compared with current census size or effective population size (Cagan et al. 2016).

## Discussion

In this article, we comprehensively and conservatively analyzed a chosen set of 123 inner-ear-expressed genes in 27 primate species. We performed both site and branch-site test analyzes, aiming to establish selection patterns both at the gene and at the clade/lineage levels. We found signals of selective pressure, and, building upon the expression characteristics and the environment of the concerned species, we hypothesize some macroscopical factors from which this selective pressure may stem.

### Questioning Auditory Gene Selection and the Impact of Confounding Factors

#### Auditory Gene Selection

We chose to analyze a subset of genes highly expressed in the inner ear, gathered in the EARxpress repository to include the maximum number of primate species and to give us the opportunity to explore alignment by visual inspection due to the highly heterogeneous sequencing qualities. By focusing on genes displaying a strong expression pattern in the inner ear, but also known to be transcribed and associated with “hearing” Gene Ontology term (GO), we aimed to target key auditory genes, without any a priori in regard to the literature. We confirmed the impact of adaptive selection on a few auditory genes previously revealed in Homininae and other mammals. We highlighted new genes and new species to consider in relation with their functions and ecology, respectively. Further analyzes on a much larger set of genes expressed in hearing would help to progress further in identifying the key gene network involved in auditory function for different species and their associated habitat and social structure.

#### Impact of Alignment and Multiple Test Corrections

We estimated the relative impact of the main sources of ambiguities stemming from the large number of species analyzed. For both site and branch-site tests, correcting for multiple tests was the most severe filtering step, removing 8% and 22% of the initially captured PSGs, respectively (fig. 1). In contrast, correcting for alignment ambiguities using automatic filtering methods had a marginal impact on the final list of the selected PSGs, impacting no more than 1% of the initial candidates. However, the final visual inspection had a stronger impact, filtering out more than 10% of the remaining PSGs in branch-site test analyzes. Similar filters have been used in previous studies (Daub et al. 2017; Van Der Lee et al. 2017), in which alignment ambiguities due to incorrect sequences, frameshift or alternative exons are often mentioned as the most problematic sources of false PSGs (Kosiol et al. 2008; Wong et al. 2008; Mallick et al. 2009; Markova-Raina and Petrov 2011). If we had considered only automatic filtering methods (HMMCleaner and GUIDANCE), without considering visual inspection, this would have had much less of an impact on our results, as has been noted in the literature (Fletcher and Yang 2010). The drastic effect of this step attests the need for better automated methods to highlight alignment failures or additional control procedures.

#### Exploring the Impact of gBGC and Segmental Duplications on PSG Signals

We tested the impact of gBGC and SD on PSG signals. gBGC is known to affect around 10% of the primate genomes (Galtier et al. 2009); however, it was not detected as acting independently in our study, but rather as always coinciding with positive selection, as also noticed by Van Der Lee et al. (2017). Similarly, SDs as a proxy for duplication processes modestly impact our estimates, as also shown in Daub et al. (2017). However, the large fraction of unplaced scaffolds in many genomes, limits the output of realistic SDs maps and the conclusions that we can draw from it (from 0.37% to 100%, in base pairs, of nonchromosomal top-level scaffolds, supplementary table 9, Supplementary Material online). We found evidence of duplication for only one exon containing positively selected sites *ADGRV1* in the black snub-nosed monkey (supplementary table 8, Supplementary Material online). This result indicates either the low incidence of SDs in the set of analyzed genes, or the large underestimation of duplications in absence of reliable structural variation maps for the genomes of the primates analyzed in our study. More investigations are needed to determine whether evidence of duplication for *ADGRV1* in the black snub-nosed monkey could have potentially biased our results by considering a very close paralogous gene, or whether this gene could be Copy Number Variant (CNV) and should be considered as a driving force for this gene. The significant relationship between duplications, CNV, and adaptive traits (Prunier et al. 2017) and especially of the key role played by CNV in climatic and environmental adaptation should be kept in mind for future studies focusing on auditory genes, especially for species leaving in extreme environments, such as the black snub-nosed monkey. GC-biased gene conversion, also known to mimic positive selection through the biased fixation of GC sites has been investigated previously (Ratnakumar et al. 2010; Lartillot 2013; Romiguier and Roux 2017). In our study, none of the PSGs detected display an unambiguous gBGC signal, but are rather always, when present, equiprobable with a positive selection signal and could not help us to conclude on any potential impact of this force here. Collecting both high-quality assembled genomes and associated genome annotation, for the low-profile primate genomes studied here, and more specifically in Strepsirrhini, would contribute to better discussions on the impact of duplication and gene conversion on PSG estimates.

### The Impact of Adaptive Selection on Auditory Genes

**Fig. 3.**
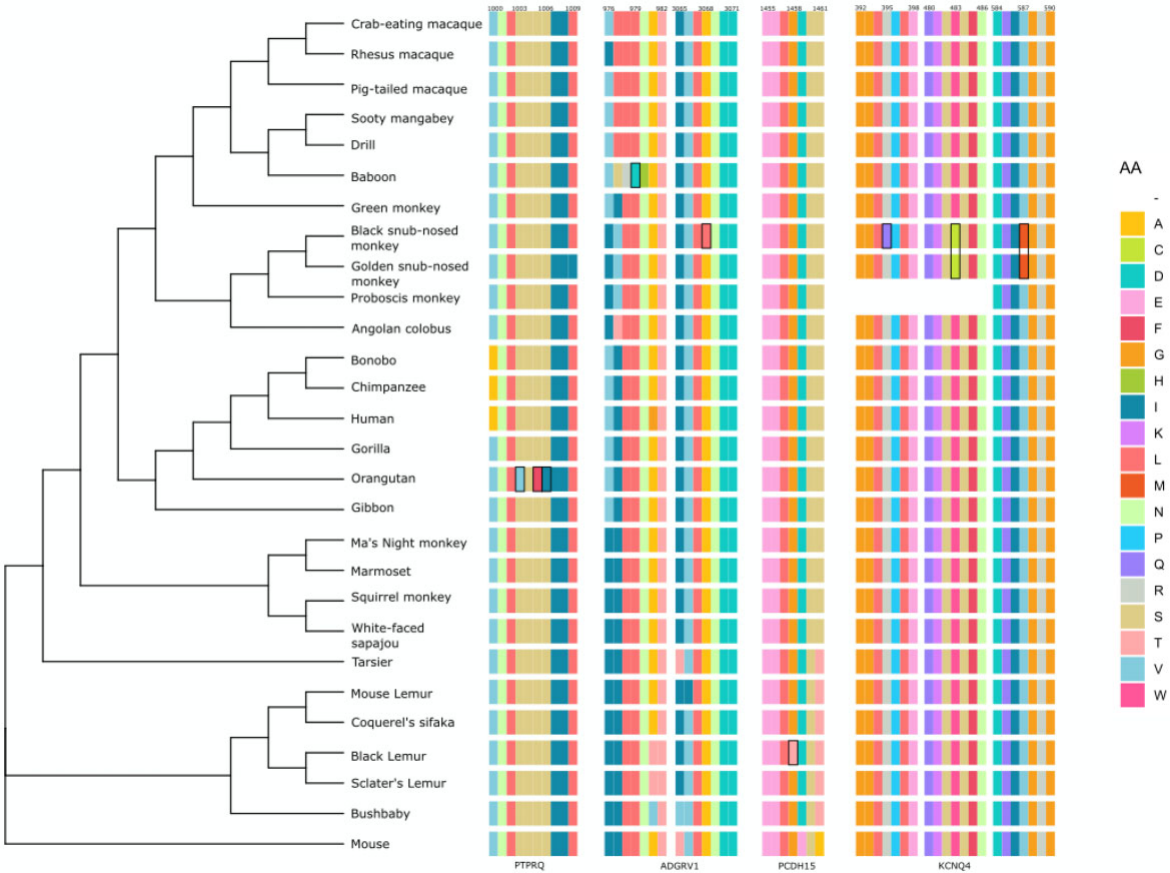
Examples of the position of selected PSG amino acid (AA) sites (in black square) in the protein sequences of the four genes: *ADGRV1*, *KCNQ4*, *PCDH15*, and *PTPRQ*.

**Fig. 4.**
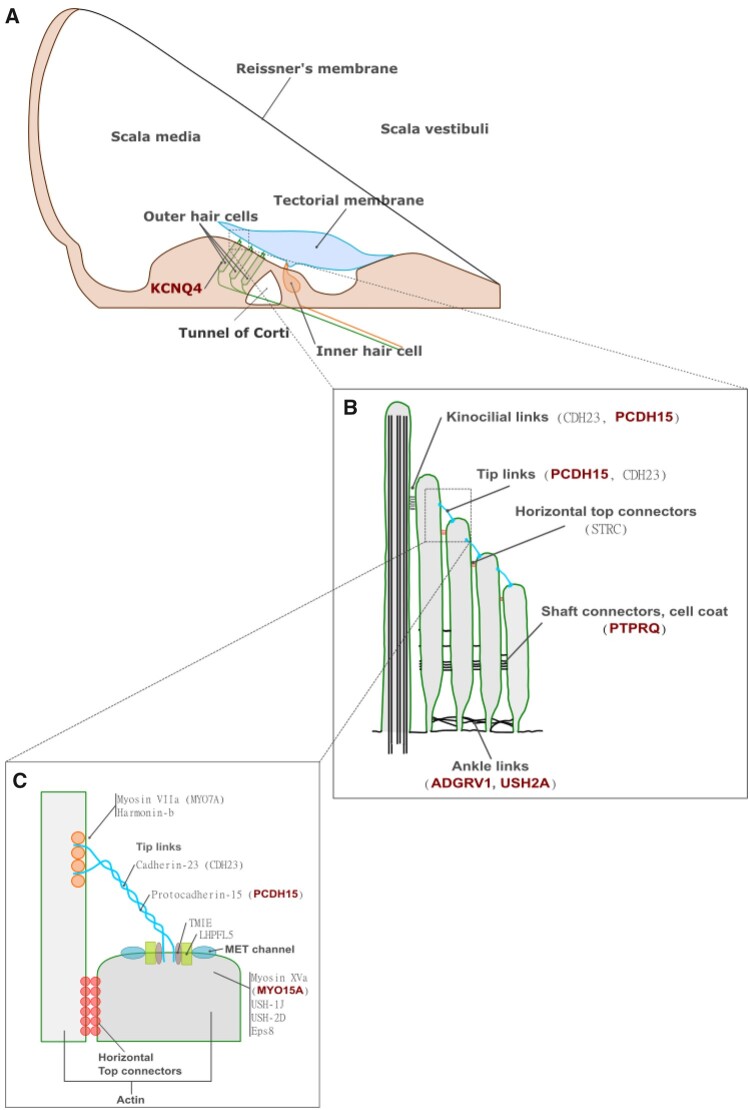
(*A*) Cochlear duct and its cellular structure including hair cell stereociliary bundles; (*B*) details of the stereocilium and hair bundles, and the structure and location of link types including kinocilial links, tip links, horizontal top connectors, shaft connectors, and ankle links (b); (*C*) Zoom on the tip link connecting two stereocilium—adapted from Fettiplace (2017) and Richardson and Petit (2019).

We found that 12% of the 123 genes analyzed show evidence of positive selection when considering the M1a-M7 models, and 21.1% when considering the M7 model alone. This estimate is four times higher than genome-wide PSGs estimates based on nine primate species (Van Der Lee et al. 2017), and nearly identical to the genome-wide estimate based on 12 primate species (Yin et al. 2020). This result stresses the importance of the number of species considered, and the procedure used to treat confounding factors (Arbiza et al. 2006). We chose a conservative approach, favoring to err in the direction of underdetection. However, the proportion of PSGs found in this study appears quite high despite neurological and auditory genes being one of the most enriched sensory families with 8.5% of genome-wide PSG estimates in mammals, compared with chemical perception (6%), smell (3.75%), or taste (2%) (Kosiol et al. 2008). The impact of positive selection on the auditory genes in primates could reflect the adaptation of the inner ear to the spectra of perceived frequencies encountered in the ecology of each species (Ramsier et al. 2012; Brown and Waser 2017; Ramsier and Rauschecker 2017) that ranges from 28 Hz to 65 kHz. Hearing sensitivity in these clades has evolved in parallel with key changes in morphology and cellular structure of the inner ear (Vater et al. 2004; Köppl and Manley 2019), detectable by selective signatures on the genomic fraction involved in inner ear functioning (Kosiol et al. 2008; Pisciottano et al. 2019). This evolution could be linked to the sound propagation in their respective biotope and species-specific needs in term of acoustic communication (Waser and Brown 1986). Five genes (*ADGRV1*, *USH2A*, *PCDH15*, *PTPRQ*, and *ATP8A2*), predicted to be functionally associated (supplementary fig. 36, Supplementary Material online), showed evidence of adaptive selection in several lineages and could reflect convergent adaptive evolution acting on the whole complex of links connecting the stereocilia (figs. 3 and 4). Most of the PSGs show species-specific patterns (20/31 One-time PSG) and might reflect the need to adapt differently at the genetic level, whatever the environment, a result that has already been detected and discussed in primates (Cagan et al. 2016). Alternatively, these regions may represent cases of convergent evolution, where the same phenotype is positively selected across species (Cagan et al. 2016). Specific targets in the inner ear are discussed later in this article.

### PSG Realms: Hair Cells, Stereocilia, and Mechanotransduction

The mechanotransduction process occurs in hair cells and stereocilia structures; atmospheric waves are transformed into a nervous message that is then sent to the brain. These cells and structures are also involved in signal amplification and modification, and alterations of these cellular components could explain the different modalities of hearing perception (frequencies and amplitude) between primate families (Coleman and Ross 2004; Fettiplace 2017). Each hair cell carries hundreds of stereocilia, organized either in three rows at their apical pole (OHC scheme), or in a staircase pattern with multiple rows (IHC scheme), connected to their neighbors by extracellular filaments, hair bundle links (Fettiplace 2017; Richardson and Petit 2019). The development of hair bundles is characterized by transitory links such as ankle links, shaft connectors, horizontal top connectors, tip links, and kinocilial links (Richardson and Petit 2019). All these types of links are required to maintain the structure, function, and normal development of hair bundles (Richardson and Petit 2019). The latter consist of actin-rich stereocilia and a microtubule-based kinocilium. The PSGs protocadherin 15 (*PCDH15*) and cadherin 23 (*CDH23*) form the kinociliary link complex between the kinocilium and the longest stereocilia, as well as the tip links that connect stereocilia (Fettiplace 2017; Richardson and Petit 2019) (fig. 4). The *ADGRV1* (also called *VLGR1* or *GPR98*) and *USH2A* compounds are found at the base of the stereocilia, where they are thought to form ankle links (Richardson and Petit 2019). Myosins apply tension on the actin filaments and are also implicated in the stereocilia length (Michalski and Petit 2015). Myosin 6 (*MYO6*) is highly concentrated in the cuticular plate at the apical hair cell surface, but is also localized in stereocilia. *MYO7A* is expressed throughout stereocilia and is notably present in ankle links in some auditory epithelial. Most of the PSGs detected correspond to key proteins involved in mechanotransduction (Fettiplace 2017) and hair bundle structure, and the recurrent PSGs suggest recurrent changes affecting these genes in multiple lineages. Among the stereocilia links, the component of the shaft connectors, *PTPRQ*, is also positively selected and plays an important role in the maturation of hair bundles (Richardson and Petit 2019). In our study, most of the components of these links are positively selected, showing that the development of the stereocilia and their cohesion could have undergone a specific adaptation in some lineages. The selection signatures of this type of link component is not specific to a given primate hearing range, but is found multiple times in their phylogenetic tree, which points to the recurrent adaptation of this set of genes to external constraints. Further investigations on the relation between transient links, actin-binding proteins, and stereocilia length will be of importance to link observed hearing sensitivity differences to molecular and cellular characteristics.

### Adaptation of Hair Cells and Hair Bundles to Extrinsic or Intrinsic Parameters

The PSGs detected in the 27 analyzed species are heterogeneously distributed among the primate phylogenetic tree. Going further in interpreting such heterogeneous distribution in regard to the ecological parameters would require accessing the whole-genome auditory-linked PSGs for populations and species living in distinctively identified ecological niches. Nevertheless, it worth noticing that the most affected species is the black snub-nosed monkey which lives at the highest altitude of any known nonhuman primate. High altitude is responsible for various physiological adaptations in many organisms (Beall 2006; Qiu et al. 2012; Ai et al. 2014); however, few studies have supported the strong effects of high altitude on the inner ear (Cingi et al. 2010; Fan et al. 2016). The exception is the domestic Yak: this species lives at a similar altitude range as the black snub-nosed monkey and presents an accelerated rate of evolution for its genes involved in sound perception (Qiu et al. 2012). If altitude is a key factor modeling auditory genes, we should notice that the black snub-nosed monkey and its closest relative, the golden snub-nosed monkey, displaying a totally different PSG pattern, have divergent phylogenetic histories and are now living in different habitats. The black snub-nosed monkey lives at higher altitudes (3,400 to 4,600 m) and faces environmental challenges such as low temperature and oxygen levels (Zhou et al. 2016), whereas the golden snub-nosed monkey lives at more moderate elevations (1,500 to 3,400 m). To determine whether PSGs detected in the black snub-nosed monkey reflect a process of physiological adaptation and modulation in frequency or amplitude perception abilities, and the potential existence of an altitude threshold, auditory brainstem responses, or other methods to estimate the hearing sensitivity spectrum would have to be tested and compared on several individuals for these two species. The second most affected species is the bushbaby, a nocturnal species that exhibits a higher sensitivity to low frequencies (around 250 Hz) compared with diurnal species (Masters 1991; Bernardi et al. 2019). Specific constraints associated with a nocturnal life might have modulated the molecular network responsible for mechanotransduction explaining the enrichment in PSGs for this species. In addition, specificity between species could explain the different sets of PSGs detected. Different biochemical or regulatory networks could be involved; the connectivity and centrality of the genes in these networks might differ between networks, thus modulating the effect size of the genes (Olson-Manning et al. 2012). This could be especially true for genes included in more than one pathway and those influencing multiple phenotypes, such as those coinvolved in vision detection for these species, that may experience pleiotropic effects. Some PSGs that form hair bundle links are also implicated in vision; for example, *PCDH15*, *USH2A*, and *ADGRV1* are all involved in Usher syndrome, congenital deafness due to stereocilia disorganization with associated progressive retinitis pigmentosa (Fettiplace 2017). This might suggest specific hearing sensitivity characteristics associated to nocturnal life, together with a coadaptation of vision-linked expressed genes to optimize light perception. Together with *RPGRIP1L*, *CLIC5*, and *WFS1* are implicated in both hearing and visual perception, suggesting that these genes had a continuous and complex evolution pattern during primate evolution, with various adaptation phases when species converted from nocturnal to diurnal activities. Supporting this hypothesis would require more investigations on both physiological and genomic components (Nummela 2017).

## Materials and Methods

### Genes Expressed in the Inner Ear: EARxpress, a Dedicated Database

In the absence of a comprehensive repository of all genes involved in auditory perception and, more specifically, inner ear expression, we gathered all publicly available information on these genes, in primates and the mouse. Information on auditory genes were retrieved from four papers focusing on the genes expressed in the inner ear of *Macaca fascicularis* (Mutai et al. 2018), and on the transcriptomic (Liu et al. 2014), developmental (Scheffer et al. 2015), and proteomic expression of inner ear genes in the mouse (Hickox et al. 2017). We gathered 2,367 genes and consolidated them in our EARxpress database (supplementary table 1, Supplementary Material online). We retrieved orthologous loci in the human genome using Biomart when possible, and BLAST when no orthologous sequences could be detected. Genes were considered orthologous when their sequence identity exceeded 80% and the e-value of the BLAST matches remained below 0.05 (Kosiol et al. 2008). To explore the potential role played by adaptive selection on genes expressed in the inner ear in the order Primates, we chose to focus our attention on a restricted number of genes that would have a greater probability of playing a role in the functioning of the inner ear and to include the maximum number of primate species. We prioritized genes overexpressed in the inner ear with regard to other tissues and selected first the 32 genes expressed in *M. fascicularis* (Mutai et al. 2018). Then, we selected 95 additional genes common to at least three or two of the remaining studies (Liu et al. 2014; Scheffer et al. 2015; Hickox et al. 2017), or associated with a GO term related to “hearing.” This selection was made without any a priori regarding the PSGs detected in the literature or their specific functions in the inner ear. Out of these 127 preselected genes, three genes (*COCH*, *MAF*, *SLC17A8*) were common between the two data sources and were considered only once. The *DEFB122* was excluded from the following analyzes because it is a pseudogene in humans and it could not be used as a functional gene reference to retrieve functional orthologous genes in other nonhuman primate species. In this study, we considered 123 genes in the phylogenetic tree construction and selective pressures analyzes (supplementary table 2, Supplementary Material online), following the pipeline detailed in figure 1.

### Orthologous Identification, Alignment Quality, and Phylogenetic Tree Reconstruction

The orthologous coding DNA sequences (CDS) of all available nonhuman primate species were retrieved from the UCSC Genome Browser (Karolchik et al. 2004) using the Multiz tool. In total, 27 available species were retrieved: *Homo sapiens* (Human), *Colobus angolensis palliates* (Angolan colobus), *Papio anubis* (Olive baboon), *Eulemur macaco* (Black lemur), *Rhinopithecus bieti* (Black snub-nosed monkey), *Pan paniscus* (Bonobo), *Otolemur garnetti* (Northern greater bushbaby), *Pan troglodytes* (Chimpanzee), *Propithecus coquereli* (Coquerel’s sifaka), *Macaca fascicularis* (Crab-eating macaque), *Mandrillus leucophaeus* (Drill), *Nomascus leucogenys* (Northern white-cheeked gibbon), *Rhinopithecus roxellana* (Golden snub-nosed monkey), *Gorilla gorilla gorilla* (Western lowland gorilla), *Chlorocebus sabaeus* (Green monkey), *Aotus nancymaae* (Ma’s night monkey), *Callithrix jacchus* (Commen marmoset), *Microcebus murinus* (Mouse lemur), *Pongo pygmaeus abelii* (Sumatran orangutan), *Macaca nemestrina* (Pig-tailed macaque), *Nasalis larvatus* (Proboscis monkey), *Macaca mulatta* (Rhesus macaque), *Eulemur flavirons* (Sclater’s lemur), *Cercocebus atys* (Sooty mangabey), *Saimiri boliviensis* (Black-capped squirrel monkey), *Tarsius syrchta* (Philippine tarsier), and *Cebus capucinus imitator* (White-faced sapajou) (supplementary table 3, Supplementary Material online). We downloaded multiple sequence alignments using Multiz Align from the Table Browser according to the GENCODE v24 track. When multiple transcripts variants were available, we chose the variant featuring the longest open reading frame compared with the human sequence (hg38/GRCh38, December 2013). Details on the human transcripts are provided in supplementary table 2, Supplementary Material online; for seven genes and a few species, orthologous sequences were missing and the corresponding positions were filled with unspecified nucleotides (N) (supplementary table 4, Supplementary Material online). The GUIDANCE2 server was used to generate a new realignment for each gene using MAFFT (Katoh and Standley 2013). To assess the robustness of the alignments, we ran 100 bootstrap tree iterations (Penn et al. 2010; Sela et al. 2015). GUIDANCE2 ranged from 0.98 to 1 for 121 genes, with two genes, *LOR* and *NEFH*, falling below at 0.92 and 0.94, respectively, which might suggest further misalignments regarding these two genes. Additionally, regardless of their GUIDANCE score, all alignments were filtered and cleaned with HMMCleaner, using the default parameters (Di Franco et al. 2019). Ambiguous nucleotide positions were masked and replaced automatically by HMMCleaner using gaps and were considered in the following analyzes. We controlled that aligned cDNA sequences corresponded to an ORF by the systematic control of a methionine and a stop codon (see supplementary figs. 1–31, Supplementary Material online); these sequences were also compared with those from the NCBI website to be certain that the ORF was present. The last filtering step consisted in a simple visual inspection of the protein sequences alignment by using BioEdit (URL: https://bioedit.software.informer.com/; last accessed June 28, 2021) in order to eliminate artefacts due to alternative transcription or unreliable alignment surrounding the positive selected residues (Wong 2008; Van der lee 2017). We built the phylogenetic super tree from the concatenated-alignment obtained with the 123 genes using RAxML-NG v.0.6.0 (Kozlov et al. 2019), using the mouse as the outgroup (supplementary fig. 32, Supplementary Material online). To preserve the maximum number of positions in the concatenated tree, no specific filtering procedure was applied. We used the best-fit model of nucleotide substitution given by Akaike’s information criteria corrected (AICc) in the JModelTest2 (Darriba et al. 2012) to build the concatenated alignment tree with the most adapted model of nucleotide substitution, and used the APE R package to unroot the tree (Paradis et al. 2004; R Core Team 2013).

### Evolutionary Analyzes: Site and Branch-Site Tests

We used codeml from the PAML v4.9h software suite (Yang 2007) to detect the signatures of positive selection. We used the codon frequency model F3X4 and the combination of the following codeml parameters: NSsites = 0 1 2 7 8, CodonFreq = 2, clock = 0, fix kappa = 0, kappa = 0.3, fix omega = 0, omega = 0.4, method = 0, cleandata = 0. We estimated the ratio of nonsynonymous/synonymous substitutions per site (d*N*/d*S* or *ω*) using maximum likelihood (ML). The d*N*/d*S* ratio was computed for each gene, using the M0 model with the gene alignment and the phylogenetic tree built from the concatenated alignment. The branch-site test used the codon frequency model F3X4, and for the alternative model (model A, positive selection), we used the following codeml parameters: model = 2, NSsites = 2, fix kappa = 0, kappa = 2, fix omega = 0, omega = 1, method = 0, cleandata = 0. The null uses the same parameters as model A, except fix omega was fixed to 1.

### Site Test Analyzes

First, we searched for genes exhibiting sites under positive selection. Since all amino acid sites in a protein are not necessarily expected to be under the same selective pressure, we assumed heterogeneous classes of sites characterized by different *ω* (Yang and Swanson 2002). The random variation of *ω* was accounted for by using different statistical distributions implemented by Nielsen and Yang (1998) and Yang et al. (2000). We used two tests known to be particularly effective: the first compares the null model M1a (neutral; *ω* < 1) and the alternative model M2a (selection; *ω* > 1), and the second compares the null model M7 (beta distribution for *ω* < 1) and the alternative model M8 (beta and *ω* > 1). In both cases, each of the two models was separately fitted to data and the log-likelihood was computed. Then the LRT (log-likelihood ratio statistic) was approximated by a χ^2^ distribution of the two models with their respective degrees of freedom. The *P* values obtained for the two site tests were corrected using the Benjamini–Hochberg method with a criterion of FDR ≤0.05 (Benjamini and Hochberg 1995). All parameters of the evolutionary models are detailed in supplementary table 5, Supplementary Material online. After the maximum likelihood estimates (MLEs) of the model parameters were obtained, we used Bayes Empirical Bayes (BEB), selected for its ability to accommodate uncertainties in the MLEs of the parameters (Yang et al. 2005) to infer the most likely site classes for every site with *ω* > 1. We considered genes that rejected null models M1a or M7 to be positively selected, and sites with probability values ≥0.95 in BEB analyzes as evolving under positive selection. GO terms enrichment and interaction of proteins were retrieved from the STRING database (Szklarczyk et al. 2019). PSGs revealed by site test analyzes are listed in supplementary table 6, Supplementary Material online.

### Branch-Site Test Analyzes

Second, we aimed to detect which branches of the primate tree (Zhang et al. 2005) of the selected auditory genes are or were subject to positive selection. We define the “foreground branch” as the branch being tested for selection and all other branches as being the “background branches.” The null model allows *ω* to vary among sites, but fixes a set of sites where *ω* = 1 for the background and foreground branches. The alternative model lets *ω* be greater than 1 in the foreground branch, but forces *ω* = 1 in the background branches for the same sites. We computed the log-likelihood for each branch three times, then took the highest log-likelihood for the null and alternative models, as recommended by Yang and Dos Reis (2011). We then computed the LRT and compared it with a χ^2^ distribution with one degree of freedom. Since we tested a total of 52 branches for 123 genes, including 27 terminal branches and 25 internal branches, we corrected the *P* values obtained from the χ^2^ test as recommended by Anisimova and Yang (2007) using the p.adjust function and the FDR (False Discovery Rate) model (Benjamini and Hochberg 1995; R Core Team 2013). The last filtering step consisted in a visual inspection of the protein sequence alignments to eliminate artifacts due to alternative transcription or unreliable alignments surrounding the positively selected residues (Van Der Lee et al. 2017); a site was filtered out if a gap was present within three amino acids around it (Zhou et al. 2014) or if the sequence alignment surrounding the position displayed a high sequence divergence compared with the other species. For these validated PSGs, we assessed the functional consequences of the amino acid changes using Polyphen-2, a software that uses machine-learning classification to estimate the probability of a SNP being damaging based on: 1) protein sequence annotation; 2) structure; and 3) an evolutionary conserved multispecies alignment (Adzhubei et al. 2013). We checked whether these sites were present in the functional domain of the corresponding protein. The PSGs revealed by branch-site test analyzes are listed in supplementary table 7, Supplementary Material online. All validated PSGs were assigned to one of three categories: One-time PSG, a gene being observed positively selected in only one terminal branch; Recurrent PSG, a gene being determined to be positively selected in at least two independent branches; and continuous PSG, a gene whose selective signal is detected uninterruptedly in at least two external and internal connected branches, suggesting a selective signal detectable over a longer time period.

### Mitigating Potential Confounding Factors on False Detection

In addition to the previously detailed measures taken to reduce the impact of sequencing and assembly errors (MAFFT, GUIDANCE, and HMMCleaner), we investigated the potential impact of large amplicon duplications (Segmental duplications), gBGC, heterogeneous sequencing quality and, demographic histories on PSG detection. Another potential source of false PSGs is multinucleotide mutations (MNM), multiple and closely spaced substitutions in a sequence (Schrider et al. 2011). If MNM can be due to low sequencing quality in the detection of positive selection in our study, these adjacent substitutions could have either a mutational or a selective origin (Stoletzki and Eyre-Walker 2011). New models incorporating MNMs have been developed and these may help to determine the cause of adjacent substitutions (Venkat et al. 2018). However, it is still a challenge to distinguish between nucleotides affected by authentic positive selection from those caused by neutral fixation of MNMs (especially in the particular case of the amino acid serine; Rogozin et al. 2016); in addition, this bias has mainly be discussed in branch site, and not in site analyzes still poorly explored, and used in our paper (Dong et al. 2021). In consequences, we decided to rely on the conservative pipeline we had designed to attenuate the impact of all false positives, to avoid an overfiltration of potential PSG, especially when MNMs are shared, or partially shared, between neighbor species (Averof et al. 2000).

#### Impact of Large Amplicon Duplications on PSGs

We investigated the impact of large duplications (segmental duplications) on PSGs since they can 1) lead to misidentify orthologous exons across species (Conrad and Antonarakis 2007; Blake and Barolo 2014); and 2) be a fertile recombination substrate (Bailey and Eichler 2006; Marques-Bonet et al. 2009) they commonly trigger gene conversion events, which, in turn, may alter estimated evolutionary profiles. We used ASGART, an efficient tool we have developed, to quickly establish a SD map for any genome (Delehelle et al. 2018). SDs were defined as sets of segments longer than 1,000 bp exhibiting more than a 90% identity rate between each other. To assess which genes could be potentially affected by SDs, we used ASGART to establish de novo exhaustive SD maps for all the concerned genomes. ASGART was run with its default settings (probe-size = 20, gap-size = 100, min-length = 1,000), which are tuned toward relatively recent and well-conserved SDs, both direct and palindromic. All genes displaying evidence of positive selection, with at least one exon intersecting with a SD, were mentioned as duplicated in the final PSG list (supplementary table 8, Supplementary Material online). Detecting duplication events was only possible for 881 exons from 24 PSGs, when the appropriate exon annotation was available (supplementary tables 8 and 9, Supplementary Material online).

#### Impact of gBGC

A region evolving at an accelerated rate on a particular branch of a phylogenetic tree is interpreted as evidence of an underlying positive selection pressure. However, selective pressure is not the only process leading to this result; gBGC events, through the biased fixation of G and C nucleotides, can also lead to the same observation. To mitigate gBGC-originated perturbation in our branch-site tests, we tested whether: 1) selection pressure-originated patterns of substitutions; 2) biased gene conversion; or 3) a mix of these two pressures were most likely to be responsible for the observed positive branch-site test results. We used the RPHAST package (Hubisz et al. 2011; Kostka et al. 2012) to compare a neutral model of evolution with three alternative tree models reflecting these possibilities. We implemented the three models and computed the likelihoods values corresponding to each of them; likelihood ratio test (LRT) and the *P* values were computed and corrected by the False Discovery Rate method (Benjamini and Hochberg 1995). The lowest *P* value associated with the highest LRT for these three tests was chosen as the best fitting scenario, and PSGs were filtered accordingly. Results from the best-fit model and estimated parameters are provided in supplementary table 10, Supplementary Material online.

#### Genome Quality Assessment: Impact of Heterogeneous Genome Quality on PSG Detection?

Our study involves 27 genomes sequenced by multiple teams using various sequencing and assembly methods (supplementary table 11, Supplementary Material online); this variation may influence our results. In order to estimate the potential impacts of these technicalities, we computed a set of metrics using the QUAST-LG toolkit (Mikheenko et al. 2018). QUAST-LG computes two categories of metrics: first, a set of length measures based on the physical characteristics of the contigs and scaffolds, namely the N* and L* numbers, as well as the distribution of the lengths of the contigs and scaffolds. Second, leveraging the BUSCO database and when genome annotations are available, the toolkit assesses the presence or absence of a curated set of orthologous and paralogous gene families that are hypothesized to be present and well conserved in the concerned species. Using the default settings and, when available, the same genome annotations as used for the rest of the study, the computation amounted to 123 linear CPU-hours (Xeon Gold 6140@2.30 GHz), most of them being consumed during the BUSCO genes search. All metrics computed with QUAST-LG are summarized in supplementary table 11, Supplementary Material online. To avoid using specific metrics to characterize genome quality, we performed a PCA to plot the different genomes used according to their characteristics (supplementary fig. 33, Supplementary Material online). The genomes are discriminated along axis 1 by length metrics (N*, L*) and along axis 2 by BUSCO, GC content, that is, more biologically oriented metrics. To avoid any a priori on the informativeness of the metrics and due to the ongoing debate of which metrics should be used to assess genome quality (Jauhal and Newcomb 2021), each genome was considered using its spatial coordinates on the first two axes of the PCA analyzes: no correlation was detected between the number of PSGs and the coordinates along axes 1 and 2, suggesting the absence of an association between the PSG detected and the quality metrics considered here (supplementary table 12, Supplementary Material online).

#### Impact of Demography on PSG Detection

To assess whether demographic history could explain the large differences in PSG allele fixation, we tested the impact of demographic history on PSG detection. Precise estimates of census population size and effective population size (*Ne*) were not available for all the 27 species; thus, we used the IUCN status, a proxy metric for recent demographic history, combining current census size and information on habitat fragmentation (supplementary table 13, Supplementary Material online). We tested whether the number of inferred PSGs was correlated to IUCN status and found no relation between status rank and the number of PSGs detected for each species (Kruskal–Wallis, χ^2^ = 0.6056, df = 5, *P* value = 0.9877).

## Supplementary Material

Supplementary data are available at *Genome Biology and Evolution* online.

## Supplementary Material

evab133_Supplementary_Data
