# Supplementary material for: Hearing Sensitivity of Primates: Recurrent and Episodic Positive Selection in Hair Cells and Stereocilia Protein-Coding Genes
Source: Genome Biol Evol. 2021 Jun 17;13(8):evab133. doi: 10.1093/gbe/evab133 (PMC8358225; doi:10.1093/gbe/evab133)
Supplement: evab133_Supplementary_Data [file evab133_supplementary_data.zip › GBE201128_revision_Supplementary_Information_1.pdf]

Supplementary Information: Hearing sensitivity of primates: recurrent and episodic positive selection in hair cells and stereocilia protein-coding genes

Table S1. EARxpress database (in Supplementary File 2)

Table S2. The 123 selected auditory genes used in phylogenetic analyses

Table S3. Classification and genome version assembly of studied primates

Table S4. Undetected orthologous genes: concerned lineages

Figure S1-31. 31 PSGs protein alignments after all filtering.

Figure S32. Phylogenetic super-tree based on the concatenated alignment of 123 hearing genes from RAxML-NG

Table S5. Parameters used for site-test analyses

Table S6. PSGs revealed by the site test analyses

Table S7. PSGs revealed by the branch-site test analyses after alignment correction, multiple tests correction, and an ultimate visual inspection (in Supplementary File 2)

Table S8. PSGs interacting with duplications (in Supplementary File 2)

Table S9. Genomes used and their characteristics (version, proportion of unplaced fragments in the genome studied) for each primate species (in Supplementary File 2)

Table S10. PSGs tested by RPHAST revealing gBGC patterns

Table S11. Genomic information evaluated by QUAST-LG for each studied species and PSGs revealed by the branch-site test analyse after multiple test, alignment corrections and ultimate visual inspection (in Supplementary File 2)

Figure S33. PCA based on whole-genome metrics calculated with QUAST-LG from the studied primates and its PSGs

Table S12. QUAST-LG metrics used in the PCA (in Supplementary File 2)

Table S13. IUCN status of studied species and PSGs revealed by the branch-site test analyse after multiple test and alignment corrections (in Supplementary File 2)

Figure S34. Codon-based phylogenetic tree of 27 primates

Table S14. Gene- and species-specific ω values (in Supplementary File 2)

Table S15. PSGs revealed by the branch-site test analyse before any filtering (in Supplementary File 2)

Table S16. PSGs revealed by the branch-site test analyse after multiple test and alignment corrections (in Supplementary File 2)

Figure S35. Venn diagram representing shared PSGs from both site and branch-site test analyses, before (a) and after (b) filtering for confounding factors (multiple test and alignment). In bold are mentioned the PSGs with a continue signal, in Italic are the PSGs with a parallel signal and in Italic bold are the PSG with both type of signal.

Figure S36. Network based on the 31 PSGs from the branch-site test using STRING.

	Ensembl code/ GENCODE v24	CDS lenght (bp)
AADACL2	ENST00000356517.4	1473
ACBD7	ENST00000356189.6	267
ADGRV1	ENST00000405460.9	18921
AFTPH	ENST00000409933.5	2811
ALDH1A3	ENST00000329841.10	1539
ANP32B	ENST00000339399.5	756
ATP2B2	ENST00000360273.7	3732
ATP8A2	ENST00000381655.7	3567
ATP8B1	ENST00000648908.1	3756
BARHL1	ENST00000263610.7	984
BCL2	ENST00000398117.1	720
BMPER	ENST00000649409.2	2058
C1QTNF5	ENST00000528368.3	732
CACNA1D	ENST00000288139.11	6546
CALB1	ENST00000265431.7	786
CALB2	ENST00000302628.9	816
CASP3	ENST00000308394.9	834
CDH23	ENST00000224721.6	10080
CEACAM16	ENST00000587331.7	1278
CHRNA9	ENST00000310169.3	1440
CHRNB2	ENST00000368476.4	1509
CIB2	ENST00000258930.8	564
CLCA2	ENST00000370565.5	2832
CLIC5	ENST00000185206.12	1233
CLRN1	ENST00000328863.8	738
COCH	ENST00000396618.9	1653
COL10A1	ENST00000243222.8	2043
CST6	ENST00000312134.3	450
CTNNB1	ENST00000349496.11	2346
CXCL14	ENST00000337225.5	336
DNASE1	ENST00000407479.5	849
DNM1	ENST00000372923.7	2595
EBF1	ENST00000313708.11	1776
ECM2	ENST00000344604.9	2100
EGFL8	ENST00000395512.5	882
EPS8L2	ENST00000526198.5	2196
ESPN	ENST00000645284.1	2565
ESPNL	ENST00000343063.8	3018
ESRRG	ENST00000366937.5	1413
EYA4	ENST00000531901.5	1938
FAT4	ENST00000394329.7	14946

FGF9	ENST00000382353.6	627
FSCN2	ENST00000334850.7	1551
FZD6	ENST00000358755.5	2121
GABRB3	ENST00000541819.6	1590
GBX2	ENST00000306318.5	1047
GJB2	ENST00000382844.2	681
GRXCR2	ENST00000377976.1	744
IL17B	ENST00000261796.4	543
JAG2	ENST00000331782.8	3717
KCNB2	ENST00000523207.2	2736
KCNJ13	ENST00000233826.4	1083
KCNQ4	ENST00000347132.10	2088
KIAA1024	ENST00000305428.7	2751
KRT24	ENST00000264651.3	1578
LHFPL5	ENST00000360215.3	660
LHX3	ENST00000371746.9	1209
LMO7	ENST00000357063.7	5007
LOR	ENST00000368742.4	939
LRTOMT	ENST00000435085.5	876
MAF	ENST00000326043.5	1212
MCOLN3	ENST00000370589.7	1662
MLANA	ENST00000381477.8	357
MPZ	ENST00000533357.5	747
MYCL	ENST00000397332.2	1185
MYO15A	ENST00000647165.2	10593
MYO3A	ENST00000642920.2	4851
MYO3B	ENST00000408978.8	4026
MYO6	ENST00000369981.7	3888
MYO7A	ENST00000409709.9	6648
NEFH	ENST00000310624.7	3306
NEK1	ENST00000507142.5	3861
NHLRC2	ENST00000369301.3	2181
NT5DC1	ENST00000319550.9	1368
OCM	ENST00000242104.6	330
OGN	ENST00000262551.8	897
OTOF	ENST00000272371.6	5994
OTOS	ENST00000391989.6	270
PCDH15	ENST00000617051.4	5895
PJVK	ENST00000644580.1	1059
PLCE1	ENST00000371380.7	6909
POU4F2	ENST00000281321.3	1230

POU4F3	ENST00000646991.2	1017
PTN	ENST00000348225.7	507
PTPRQ	ENST00000616559.4	6999
RAB3IP	ENST00000550536.5	1431
RBMS3	ENST00000383767.7	1314
RDH10	ENST00000240285.10	1026
RIPOR2	ENST00000259698.9	3207
RPGRIP1L	ENST00000647211.2	3948
RPL38	ENST00000311111.11	213
SALL1	ENST00000251020.8	3975
SCRIB	ENST00000356994.7	4968
SH2D4B	ENST00000339284.6	1074
SH3GL2	ENST00000380607.5	1059
SHC4	ENST00000332408.9	1893
SHROOM2	ENST00000380913.8	4851
SIX1	ENST00000645694.3	855
SIX2	ENST00000303077.7	876
SLC13A4	ENST00000354042.8	1881
SLC17A8	ENST00000323346.10	1770
SLC1A3	ENST00000265113.9	1629
SLC26A5	ENST00000393727.5	2241
SLC52A3	ENST00000488495.2	1410
SOD1	ENST00000270142.10	465
SRRM4	ENST00000267260.5	1836
STOX1	ENST00000298596.11	2970
STRBP	ENST00000348403.10	2019
STRC	ENST00000450892.7	5328
TGFB2	ENST00000366929.4	1329
TMC1	ENST00000645208.2	2283
TMIE	ENST00000643606.2	471
TMPRSS3	ENST00000433957.7	1365
TTC8	ENST00000614125.4	1596
TUB	ENST00000305253.8	1686
TWF2	ENST00000305533.10	1050
TYR	ENST00000263321.6	1590
USH1G	ENST00000614341.5	1386
USH2A	ENST00000307340.8	15609
VANGL2	ENST00000368061.3	1566
WDR19	ENST00000399820.8	4029
WFS1	ENST00000226760.5	2673
ZFHX4	ENST00000651372.2	10851

Table S2: **The 123 selected "hearing" genes used in phylogenetic analyses**. Human transcript Ensembl ID version and size used in our analyses.

Genome version	Year published	Order	Suborder	Infraorder	Parvorder	Superfamily	Family	Subfamily	Tribe	Genus
cebCap1	Apr. 2016	Primates	Haplorhini	Simiiformes	Platyrrhini		Cebidae	Cebinae		Cebus
panPan2	Aug. 2015	Primates	Haplorhini	Simiiformes	Catarrhini	Hominoidea	Hominidae	Homininae	Hominini	Pan
rhiBie1	Aug. 2016	Primates	Haplorhini	Simiiformes	Catarrhini	Cercopithecoidea	Cercopithecidae	Colobinae	Presbytini	Rhinopitecus
GRCh38/hg38	Dec. 2013	Primates	Haplorhini	Simiiformes	Catarrhini	Hominoidea	Hominidae	Homininae	Hominini	Homo
papAnu3	Feb. 2013	Primates	Haplorhini	Simiiformes	Catarrhini	Cercopithecoidea	Cercopithecidae	Cercopithecinae	Papionini	Papio
ponAbe2	July 2007	Primates	Haplorhini	Simiiformes	Catarrhini	Hominoidea	Hominidae	Ponginae	Pongini	Pongo
macFas5	Jun. 2013	Primates	Haplorhini	Simiiformes	Catarrhini	Cercopithecoidea	Cercopithecidae	Cercopithecinae	Papionini	Macaca
aotNan1	Jun. 2017	Primates	Haplorhini	Simiiformes	Platyrrhini	Cebidae	Aotidae	Aotinae		Aotus
chlSab2	Mar. 2014	Primates	Haplorhini	Simiiformes	Catarrhini	Cercopithecoidea	Cercopithecidae	Cercopiithecinae	Cercopithecini	Chlorocebus
cerAty1	Mar. 2015	Primates	Haplorhini	Simiiformes	Catarrhini	Cercopithecoidea	Cercopithecidae	Cercopithecinae	Papionini	Cercocebus
colAng1	Mar. 2015	Primates	Haplorhini	Simiiformes	Catarrhini	Cercopithecoidea	Cercopithecidae	Colobinae	Colobini	Colobus
macNem1	Mar. 2015	Primates	Haplorhini	Simiiformes	Catarrhini	Cercopithecoidea	Cercopithecidae	Cercopithecinae	Papionini	Macaca
manLeu1	Mar. 2015	Primates	Haplorhini	Simiiformes	Catarrhini	Cercopithecoidea	Cercopithecidae	Cercopithecinae	Papionini	Mandrillus
gorGor5	Mar. 2016	Primates	Haplorhini	Simiiformes	Catarrhini	Hominoidea	Hominidae	Homininae	Gorillini	Gorilla
calJac3	March 2009	Primates	Haplorhini	Simiiformes	Platyrrhini	Cebidae	Callitrichidae	Callitrichinae		Callithrix
panTro5	May 2016	Primates	Haplorhini	Simiiformes	Catarrhini	Hominoidea	Hominidae	Homininae	Hominini	Pan
nasLar1	Nov. 2014	Primates	Haplorhini	Simiiformes	Catarrhini	Cercopithecoidea	Cercopithecidae	Colobinae		Nasalis
rheMac8	Nov. 2015	Primates	Haplorhini	Simiiformes	Catarrhini	Cercopithecoidea	Cercopithecidae	Cercopithecinae	Papionini	Macaca
saiBol1	Oct. 2011	Primates	Haplorhini	Simiiformes	Platyrrhini		Cebidae	Saimiriinae		Saimiri
nomLeu3	Oct. 2012	Primates	Haplorhini	Simiiformes	Catarrhini	Hominoidea	Hylobatidae			Nomascus
rhiRox1	Oct. 2014	Primates	Haplorhini	Simiiformes	Catarrhini	Cercopithecoidea	Cercopithecidae	Colobinae	Presbytini	Rhinopitecus
tarSyr2	Sep. 2013	Primates	Haplorhini	Tarsiiformes			Tarsiidae			Tarsius
eulFla1	Aug. 2015	Primates	Strepsirrhini	Lemuriformes			Lemuridae			Eulemur
eulMac1	Aug. 2015	Primates	Strepsirrhini	Lemuriformes			Lemuridae			Eulemur
micMur3	Feb. 2017	Primates	Strepsirrhini	Lemuriformes			Cheirogaleidae			Microcebus
otoGar3	Mar. 2011	Primates	Strepsirrhini	Lorisiformes			Galagidae			Otolemur
proCoq1	Mar. 2015	Primates	Strepsirrhini	Lemuriformes			Indriidae			Propithecus
mm10	Dec. 2011	Rodentia					Muridae			Mus

Table S3: **Classification and genome version assembly of studied primates**

Table S4: **Undetected orthologous genes: concerned lineages**. The missing genes have been replaced by unspecified nucleotide ('N') in the concatenated alignment.

**Gene Name**	**Absent orthologous for the species**
ACBD7	Proboscis monkey
KIAA1024	Coquerel's sifaka, Mouse Lemur
OTOS	Coquerel's sifaka
OCM	Bonobo, Green monkey
POU4F3	Crab-eating macaque
TMPRSS3	Mouse Lemur
EGFL8	Orangutan, Proboscis monkey, Black snub-nosed monkey, Golden snub-nosed monkey Baboon, Sclater's Lemur, Black Lemur, Tarsier

Figure S1: **AADACL2 protein alignment after filtering**. The black triangle indicates the beginning of the ORF and the asterisk the ending. The positively selected sites are surrounded by black and with a blue circle.

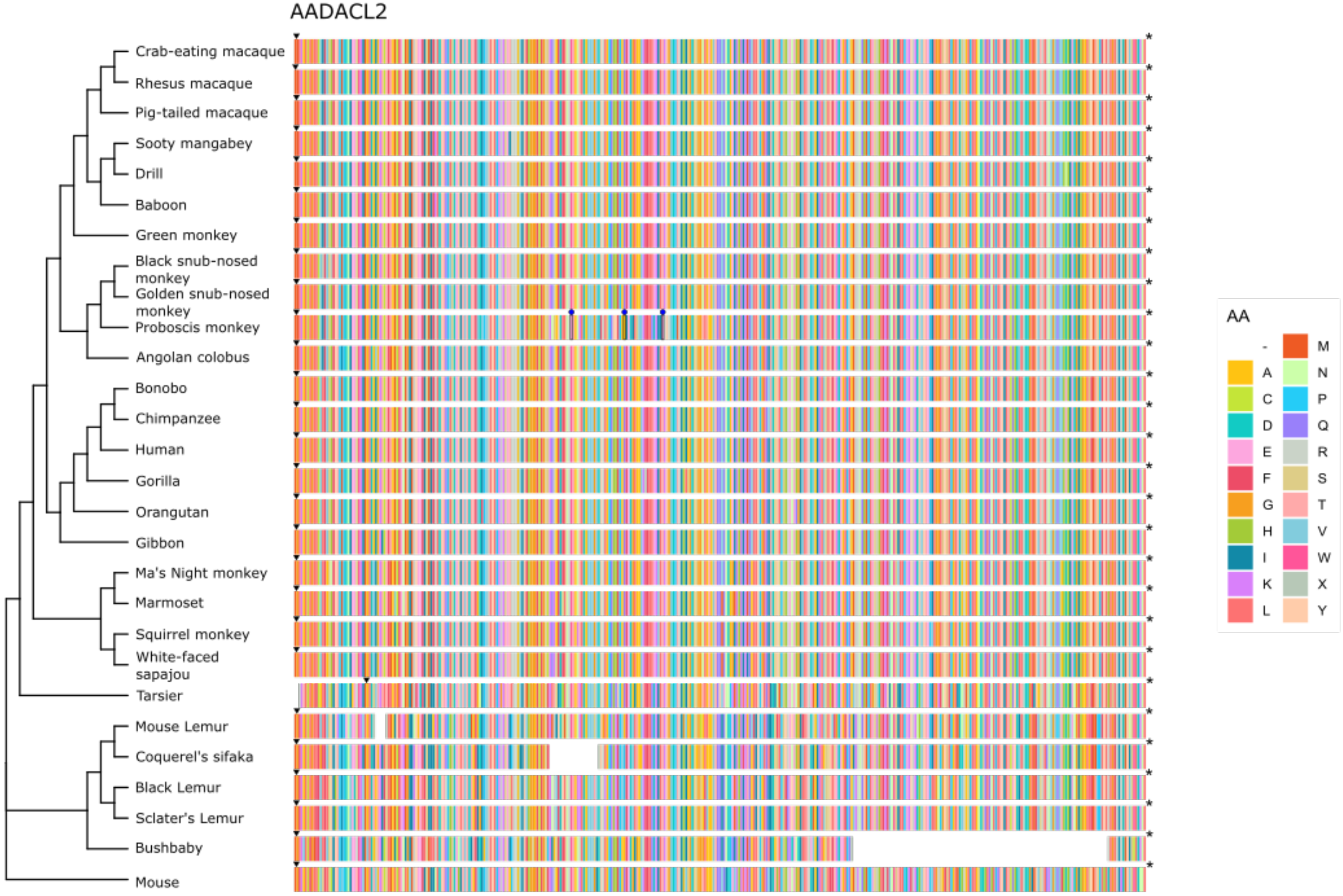

Figure S2: **ADGRV1 protein alignment after filtering**. The black triangle indicates the beginning of the ORF and the asterisk the ending. The positively selected sites are surrounded by black and with a blue circle.

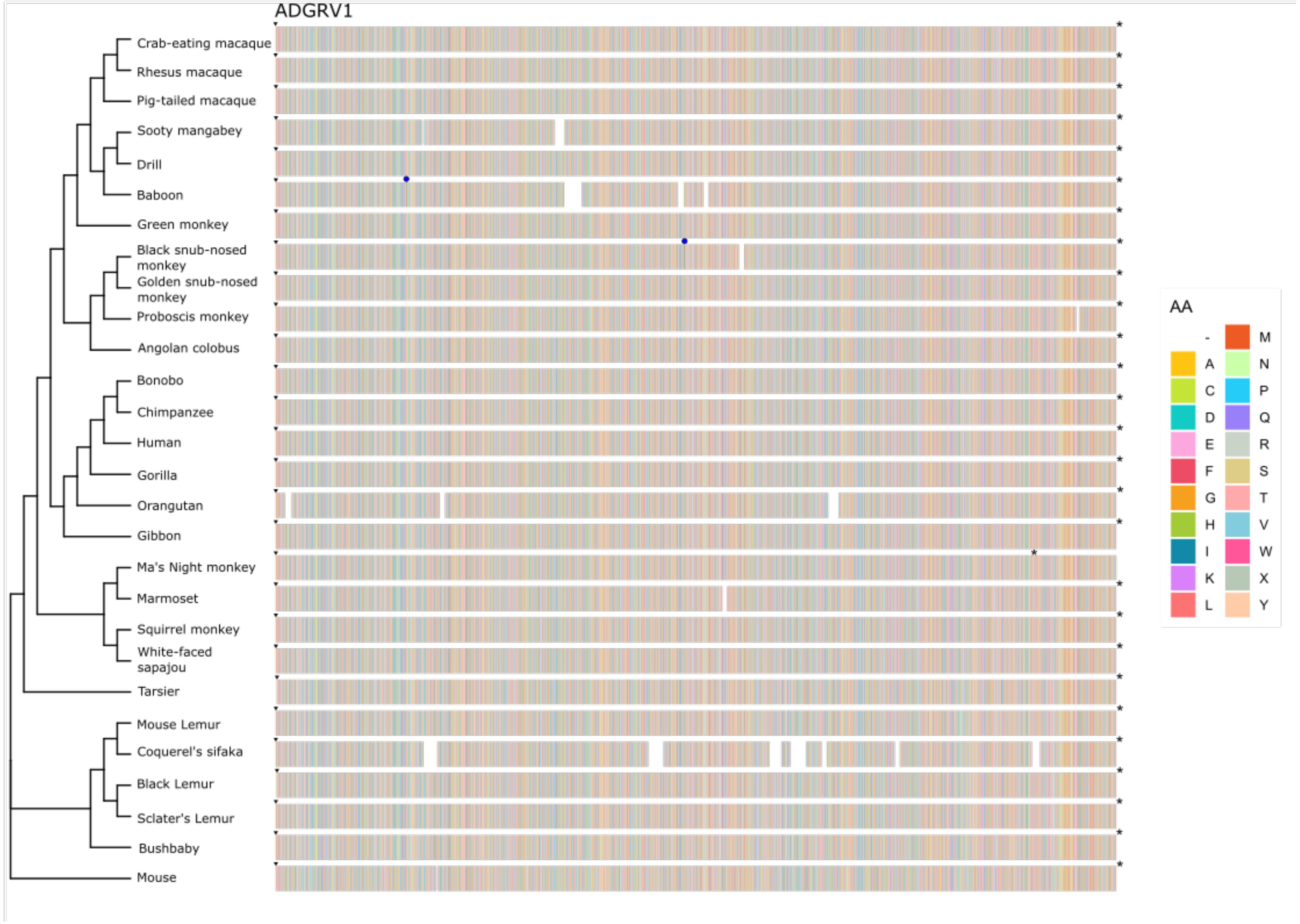

Figure S3: **ATP8A2 protein alignment after filtering**. The black triangle indicates the beginning of the ORF and the asterisk the ending. The positively selected sites are surrounded by black and with a blue circle.

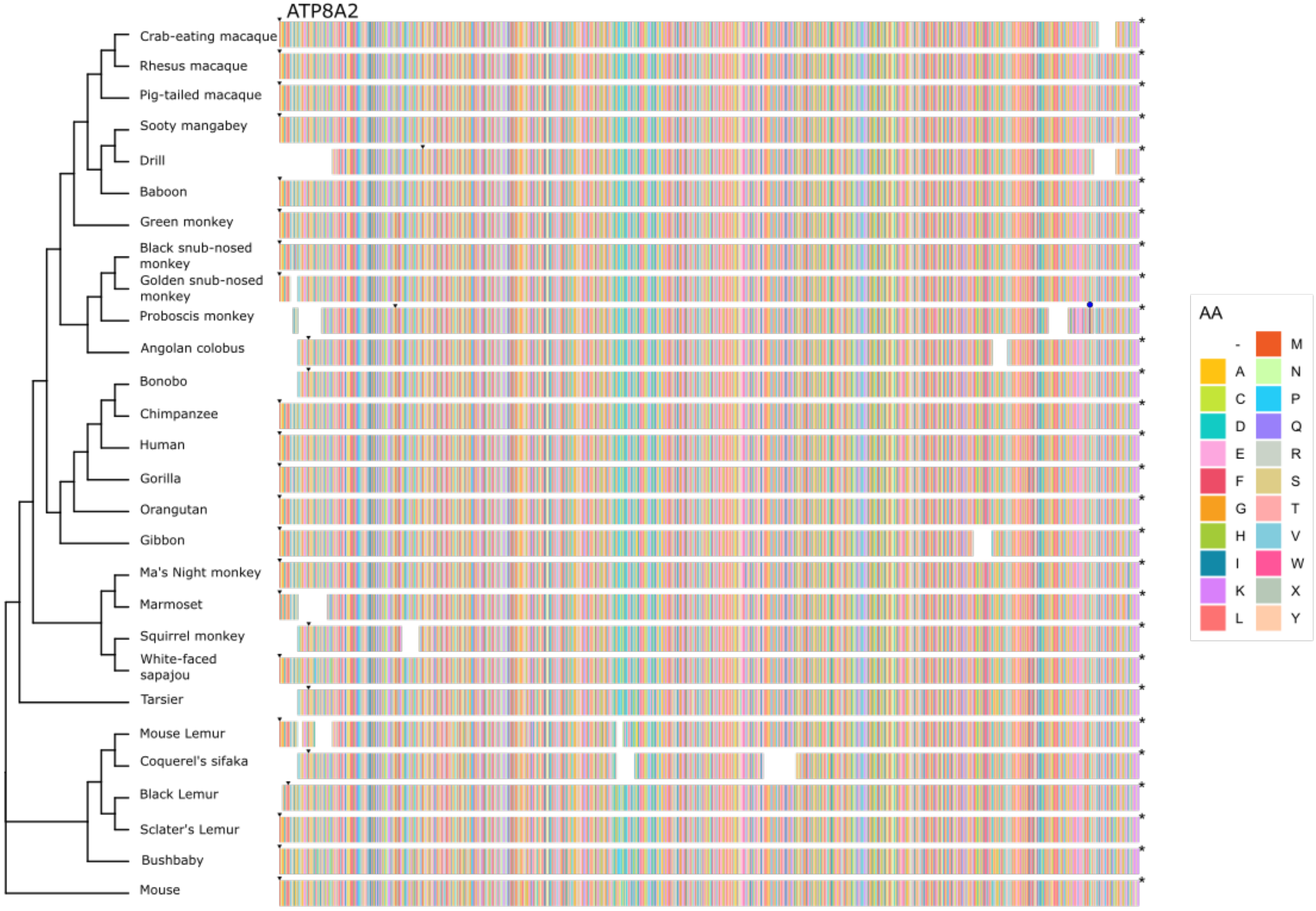

Figure S4: **ATP8B1 protein alignment after filtering**. The black triangle indicates the beginning of the ORF and the asterisk the ending. The positively selected sites are surrounded by black and with a blue circle.

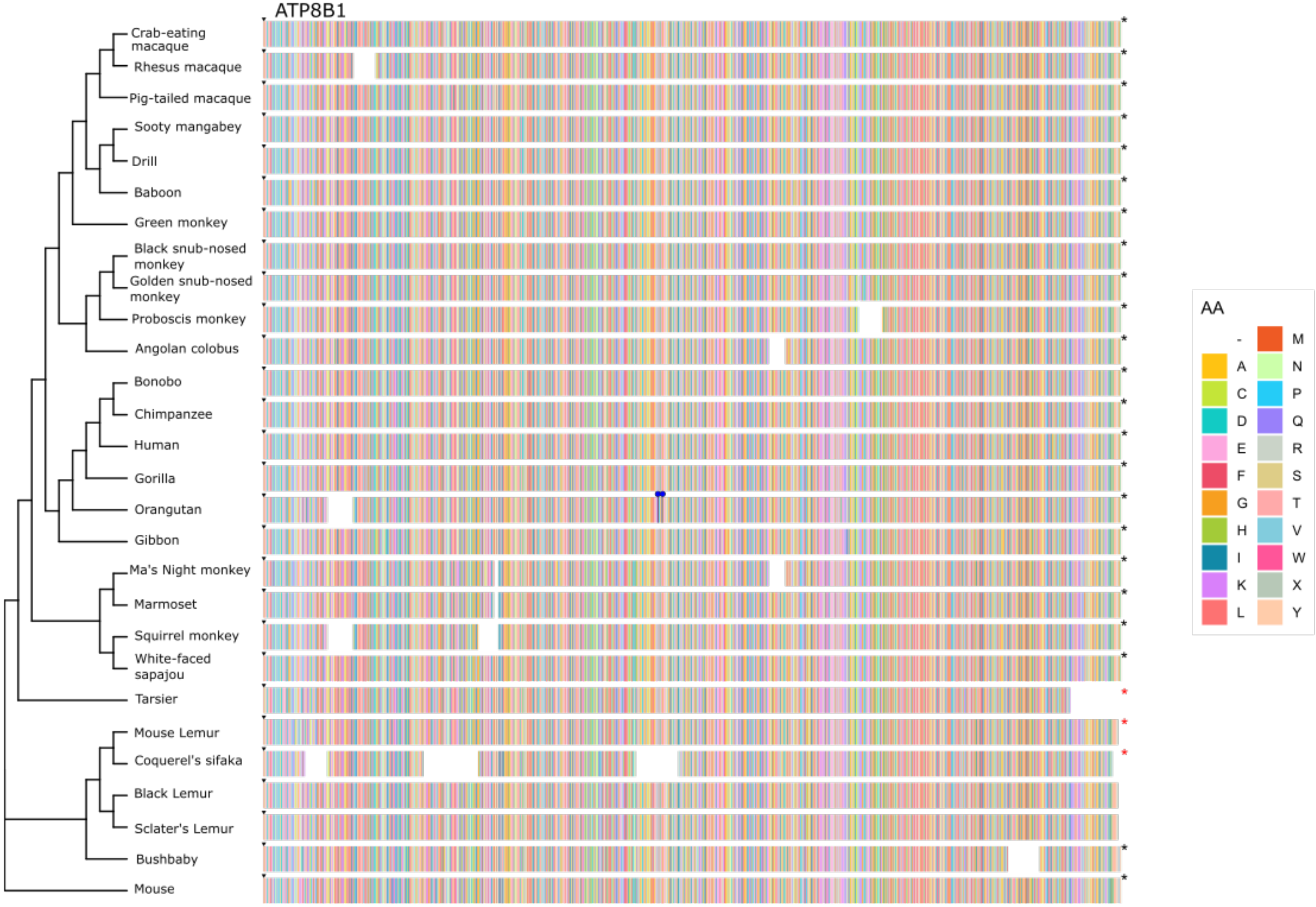

Figure S5: **CLCA2 protein alignment after filtering**. The black triangle indicates the beginning of the ORF and the asterisk the ending. The positively selected sites are surrounded by black and with a blue circle.

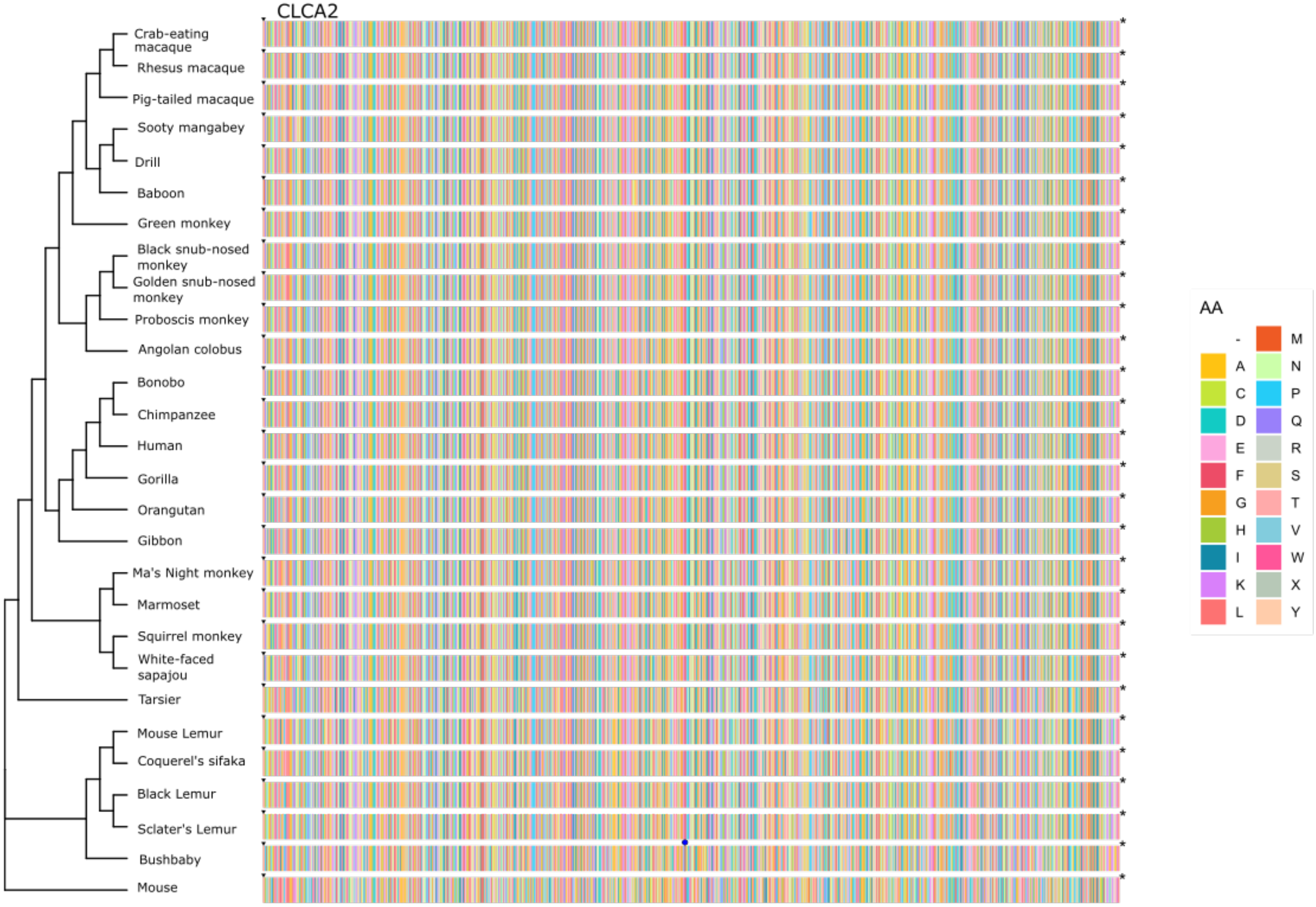

Figure S6: **CLIC5 protein alignment after filtering**. The black triangle indicates the beginning of the ORF and the asterisk the ending. The positively selected sites are surrounded by black and with a blue circle.

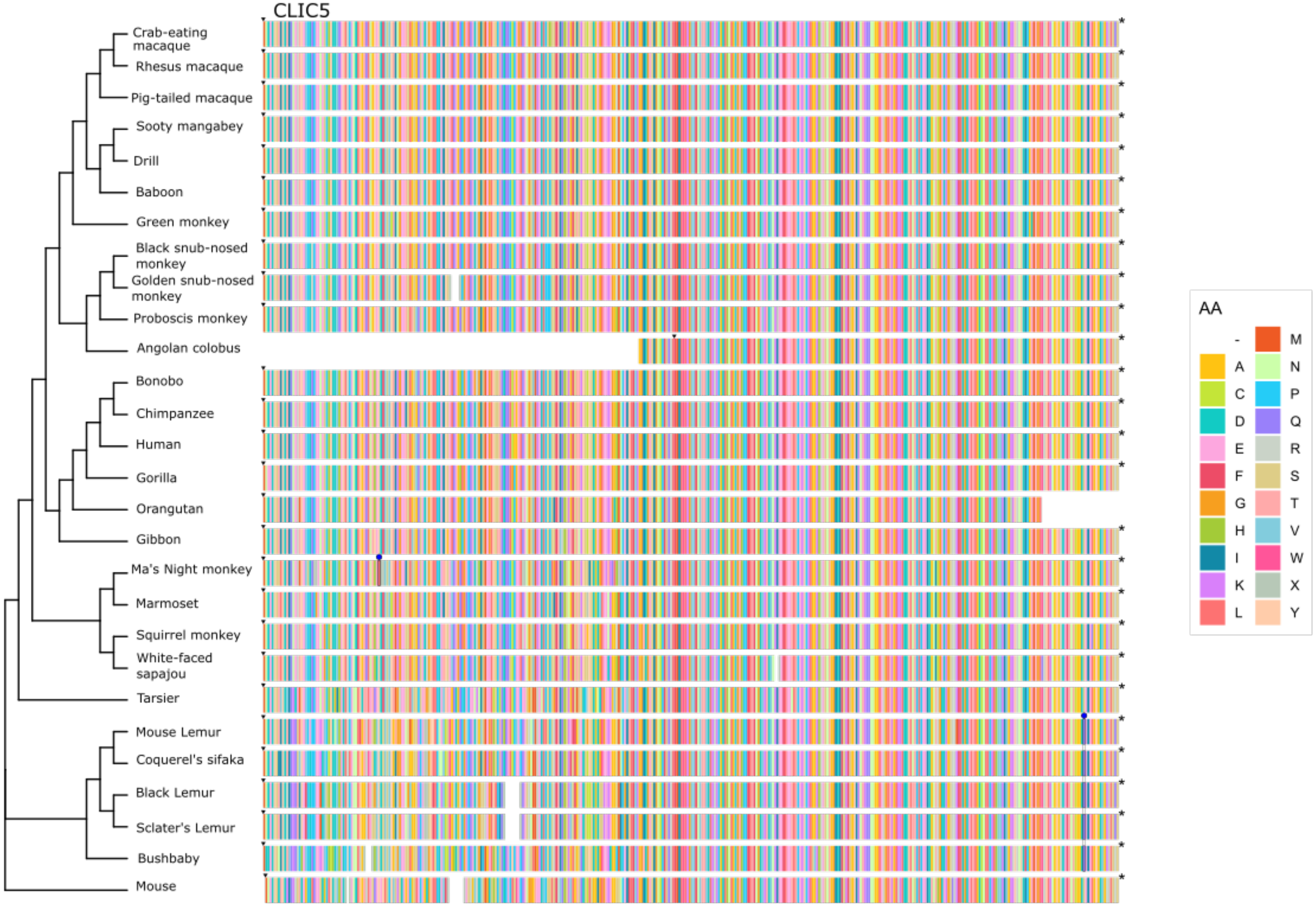

Figure S7: **COCH protein alignment after filtering**. The black triangle indicates the beginning of the ORF and the asterisk the ending. The positively selected sites are surrounded by black and with a blue circle.

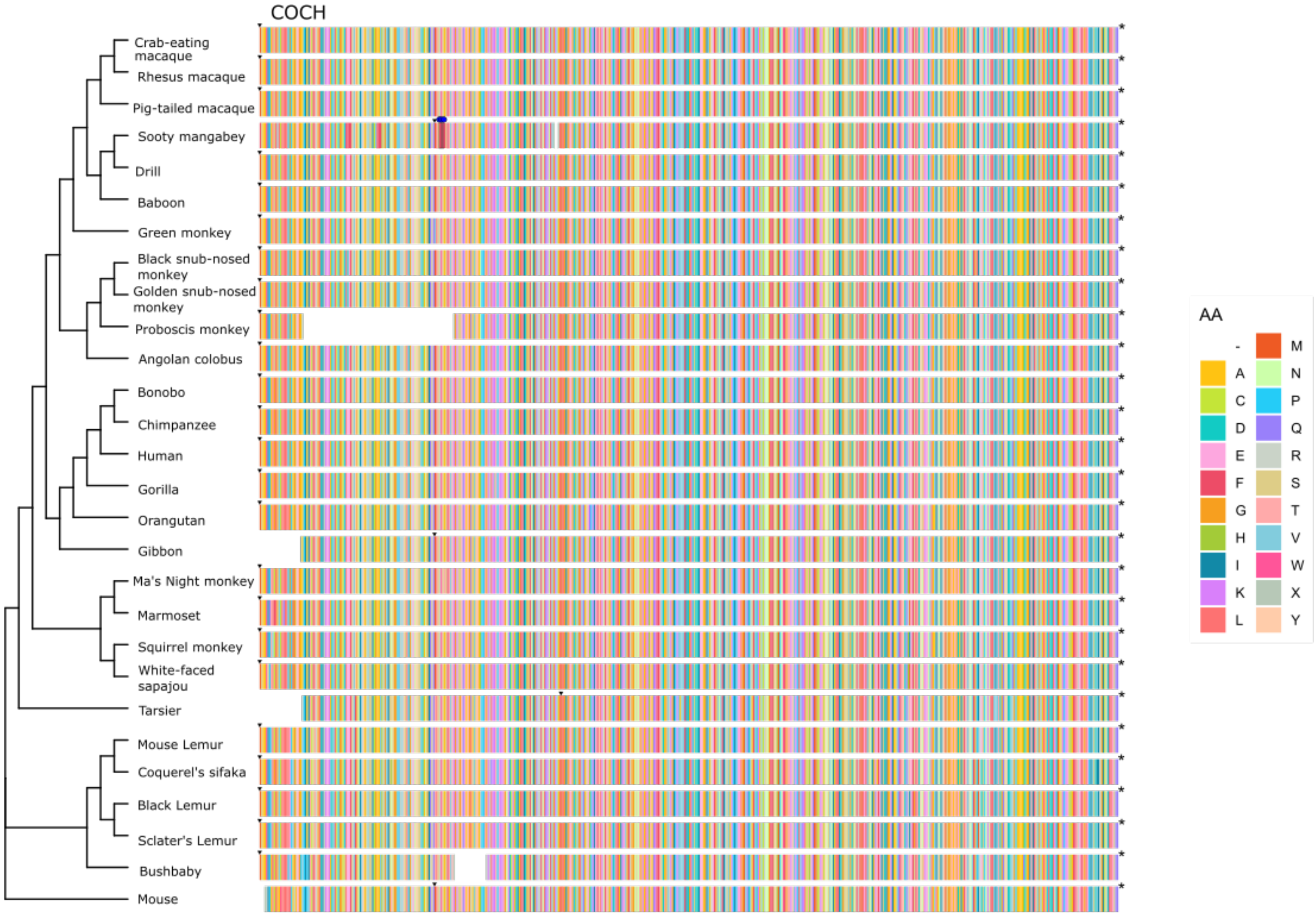

Figure S8: **COL10A1 protein alignment after filtering**. The black triangle indicates the beginning of the ORF and the asterisk the ending. The positively selected sites are surrounded by black and with a blue circle.

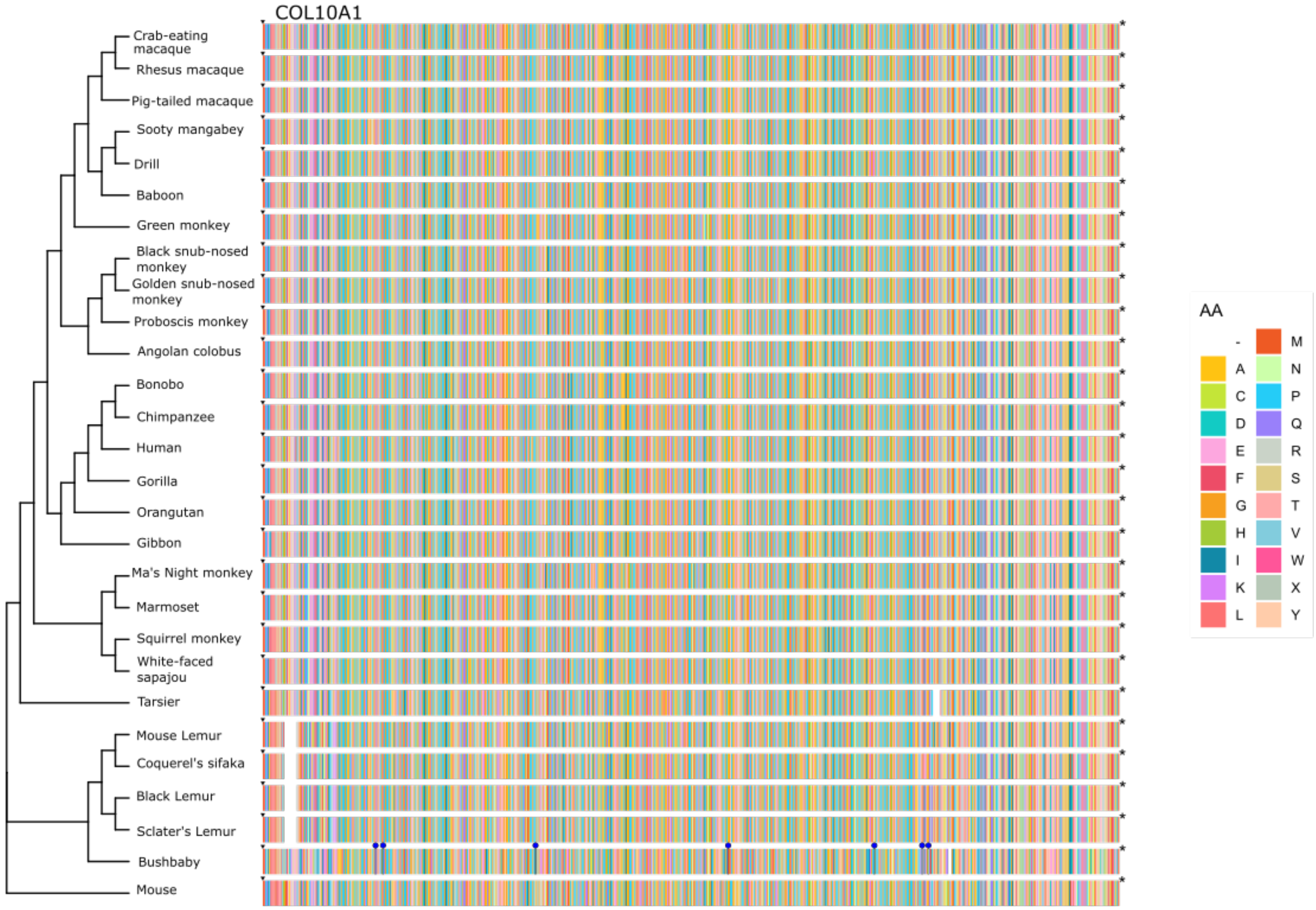

Figure S9: **DNM1 protein alignment after filtering**. The black triangle indicates the beginning of the ORF and the asterisk the ending. The positively selected sites are surrounded by black and with a blue circle.

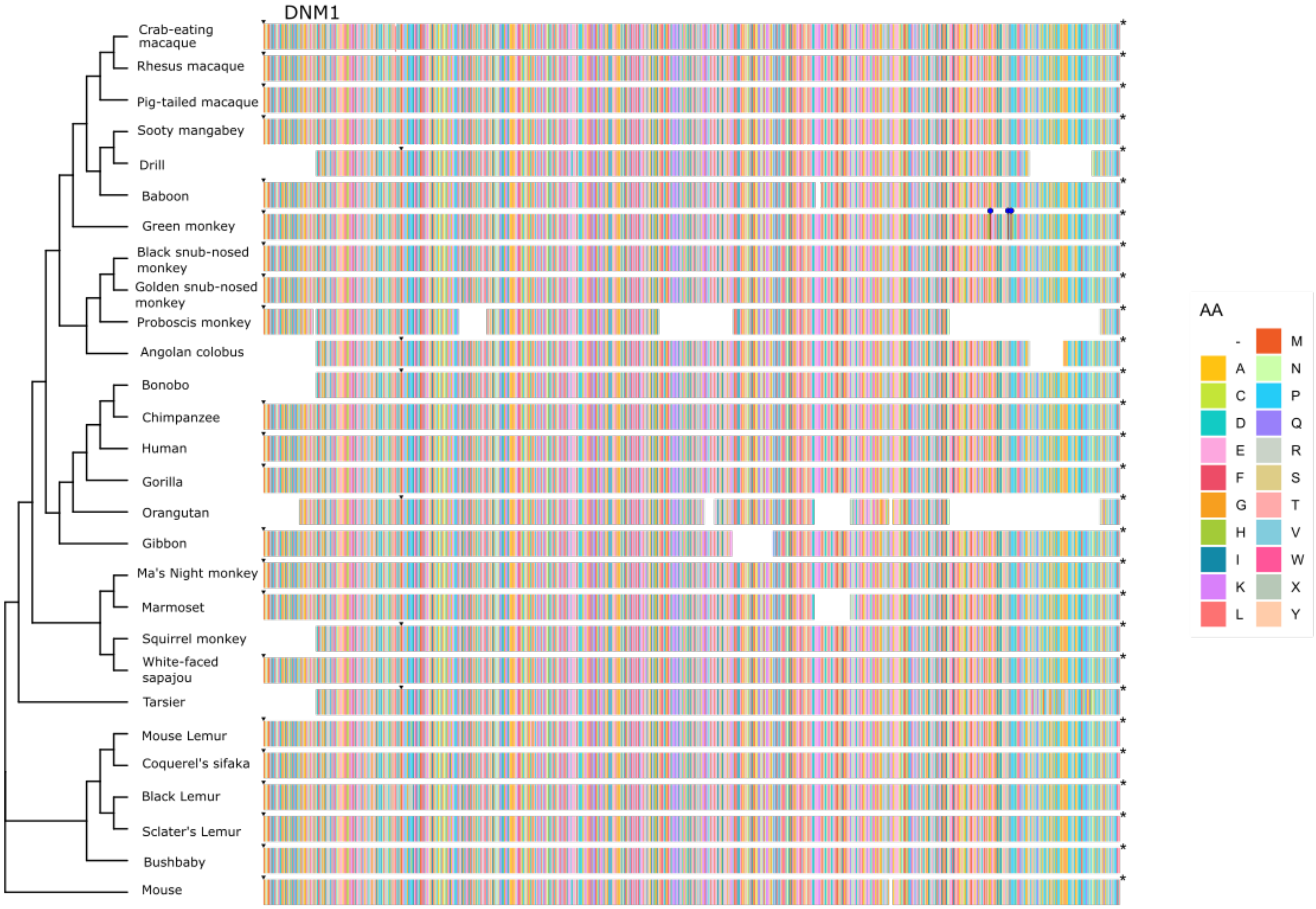

Figure S10: **FAT4 protein alignment after filtering**. The black triangle indicates the beginning of the ORF and the asterisk the ending. The positively selected sites are surrounded by black and with a blue circle.

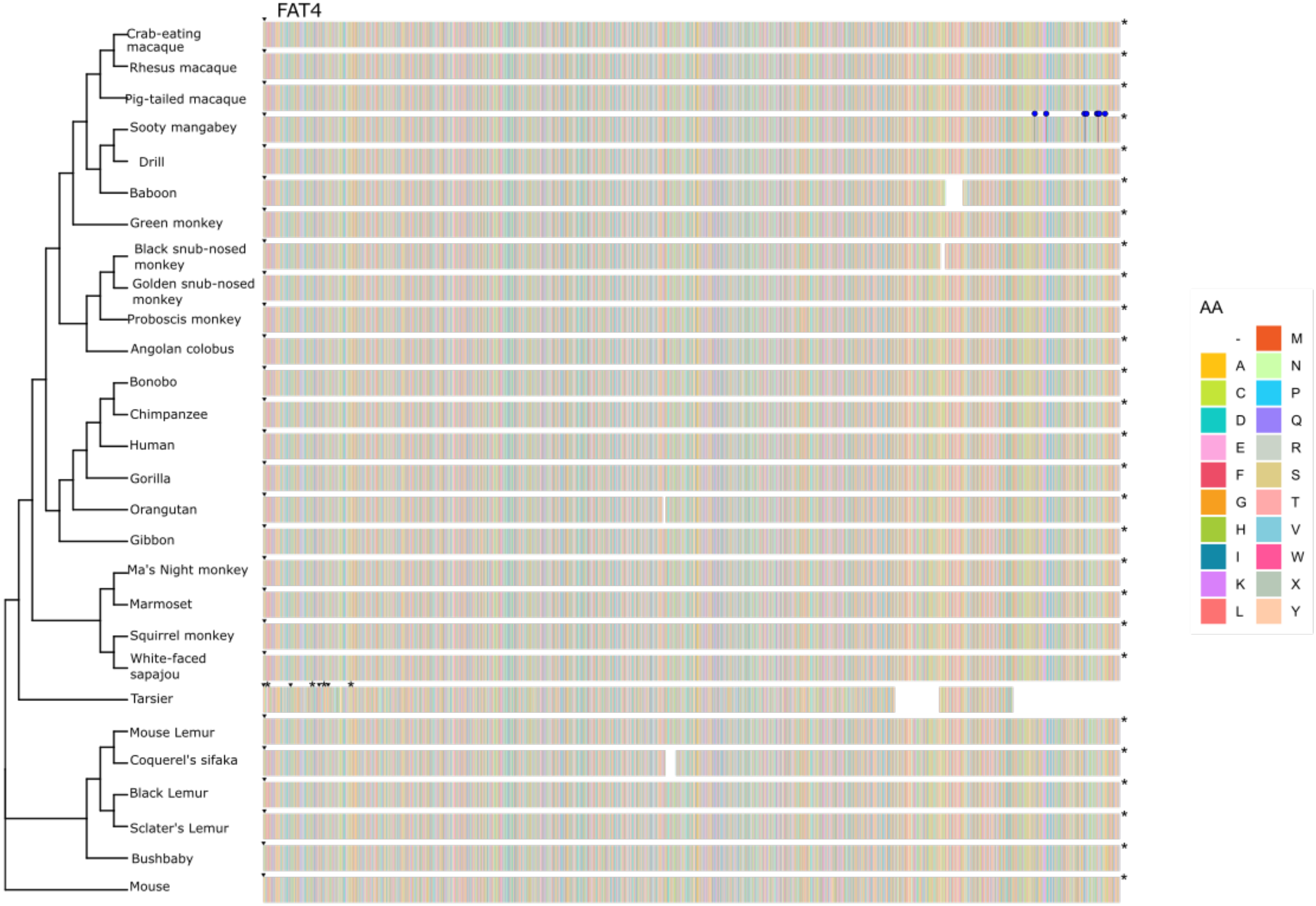

Figure S11: **FSCN2 protein alignment after filtering**. The black triangle indicates the beginning of the ORF and the asterisk the ending. The positively selected sites are surrounded by black and with a blue circle.

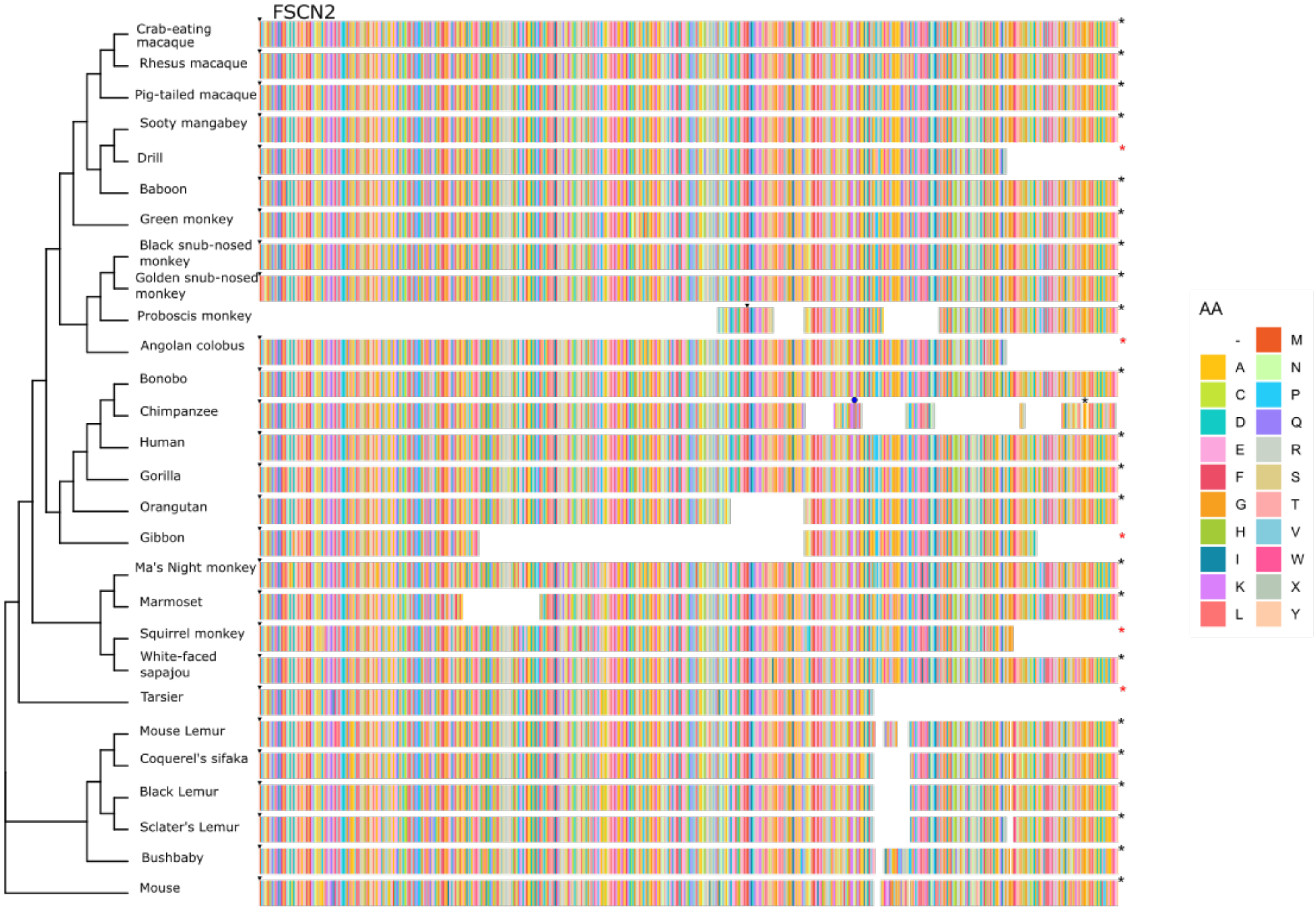

Figure S12: **KCNQ4 protein alignment after filtering**. The black triangle indicates the beginning of the ORF and the asterisk the ending. The positively selected sites are surrounded by black and with a blue circle.

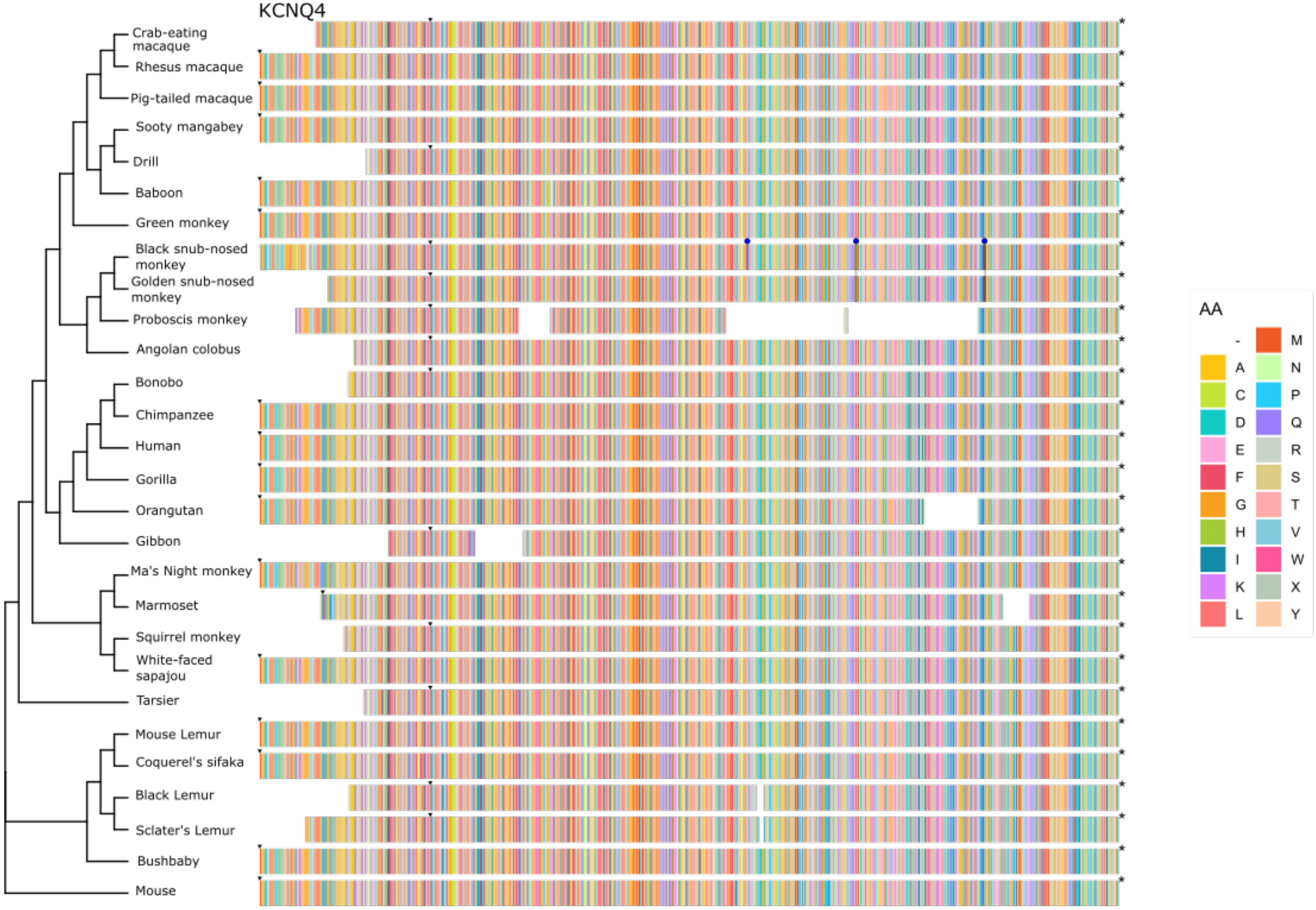

Figure S13: **LMO7 protein alignment after filtering**. The black triangle indicates the beginning of the ORF and the asterisk the ending. The positively selected sites are surrounded by black and with a blue circle.

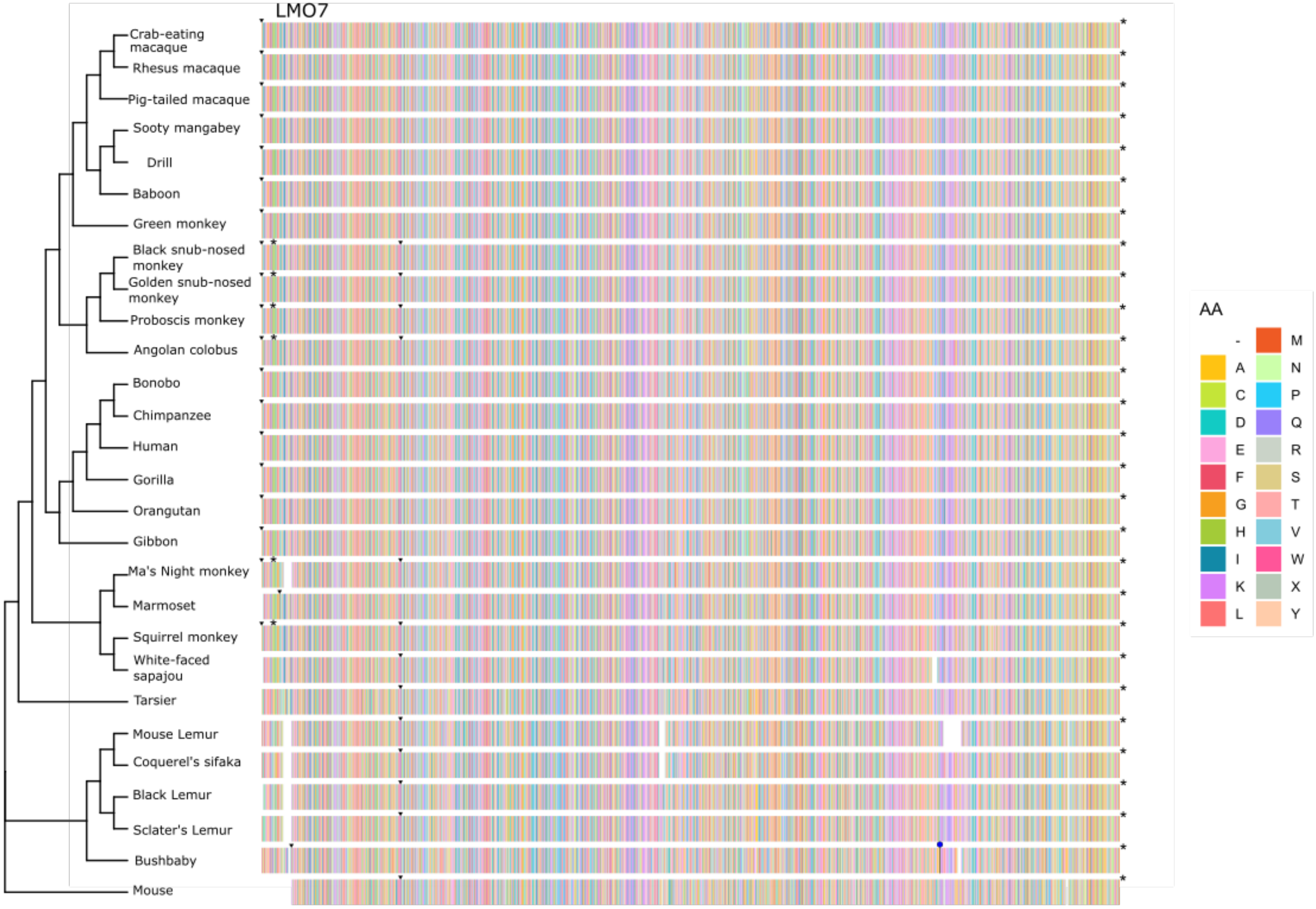

Figure S14: **MYCL protein alignment after filtering**. The black triangle indicates the beginning of the ORF and the asterisk the ending. The positively selected sites are surrounded by grey and black and with a grey circle.

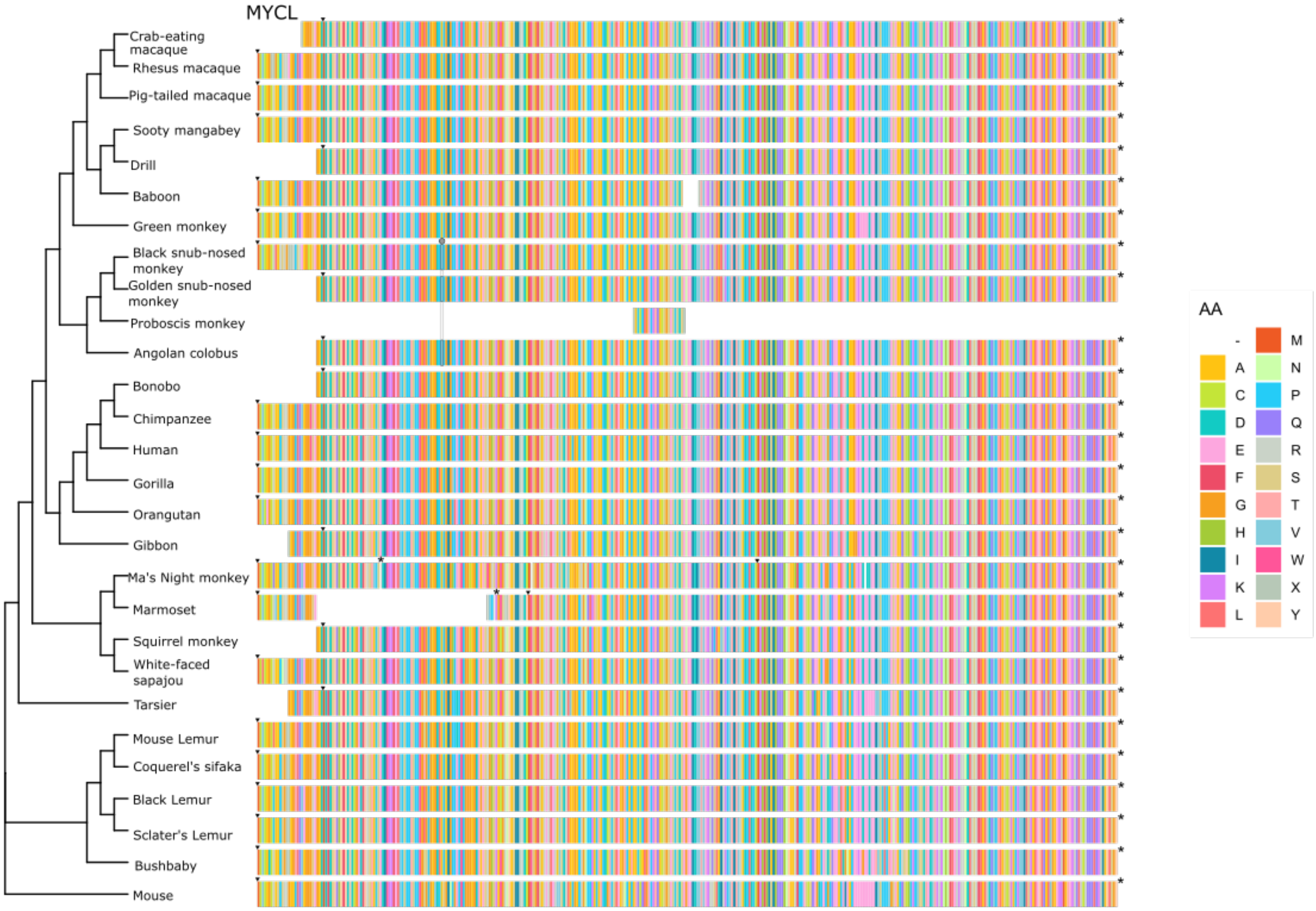

Figure S15: **MYO15A protein alignment after filtering**. The black triangle indicates the beginning of the ORF and the asterisk the ending.

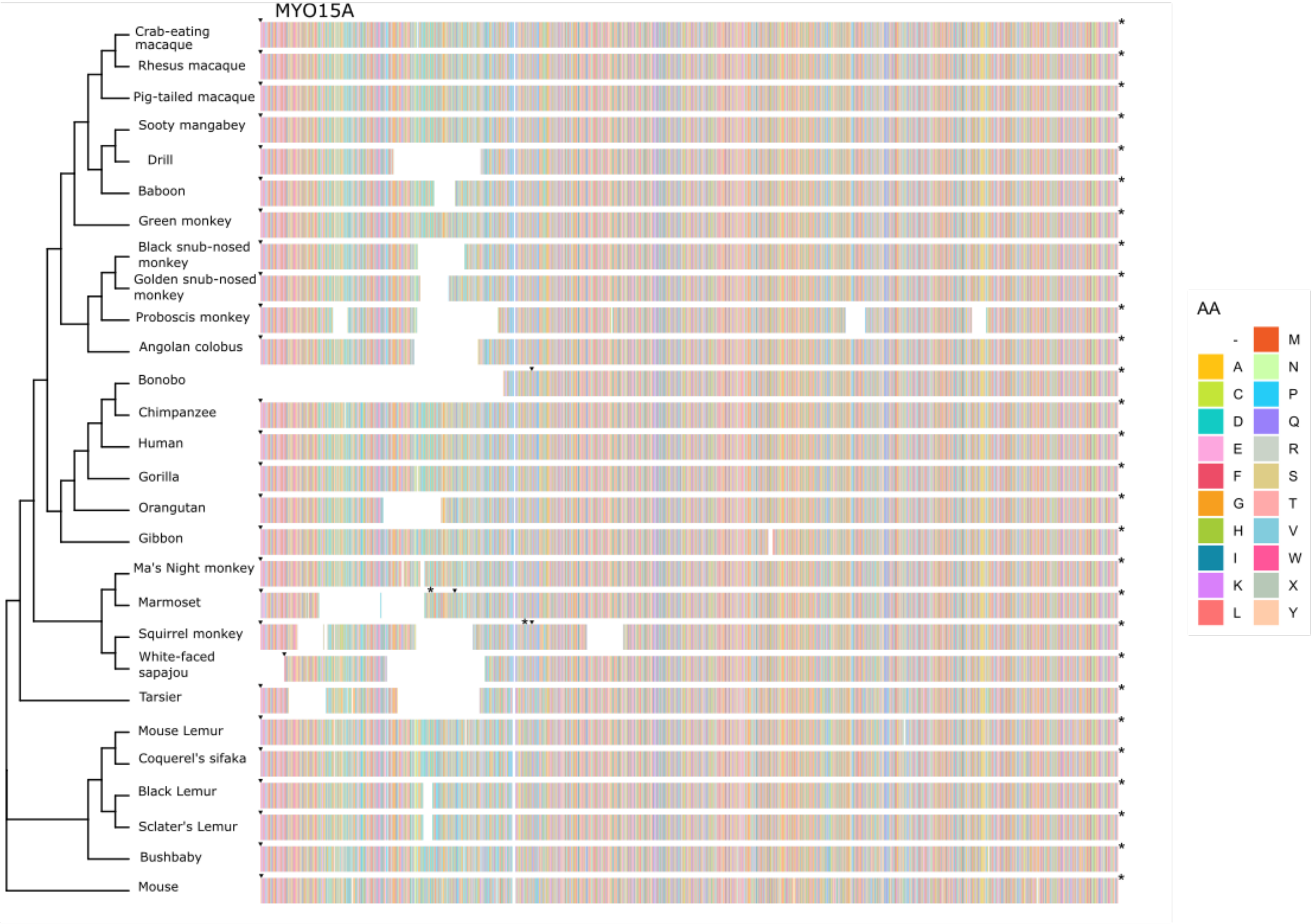

Figure S16: **MYO6 protein alignment after filtering**. The black triangle indicates the beginning of the ORF and the asterisk the ending. The positively selected sites are surrounded by black and with a blue circle.

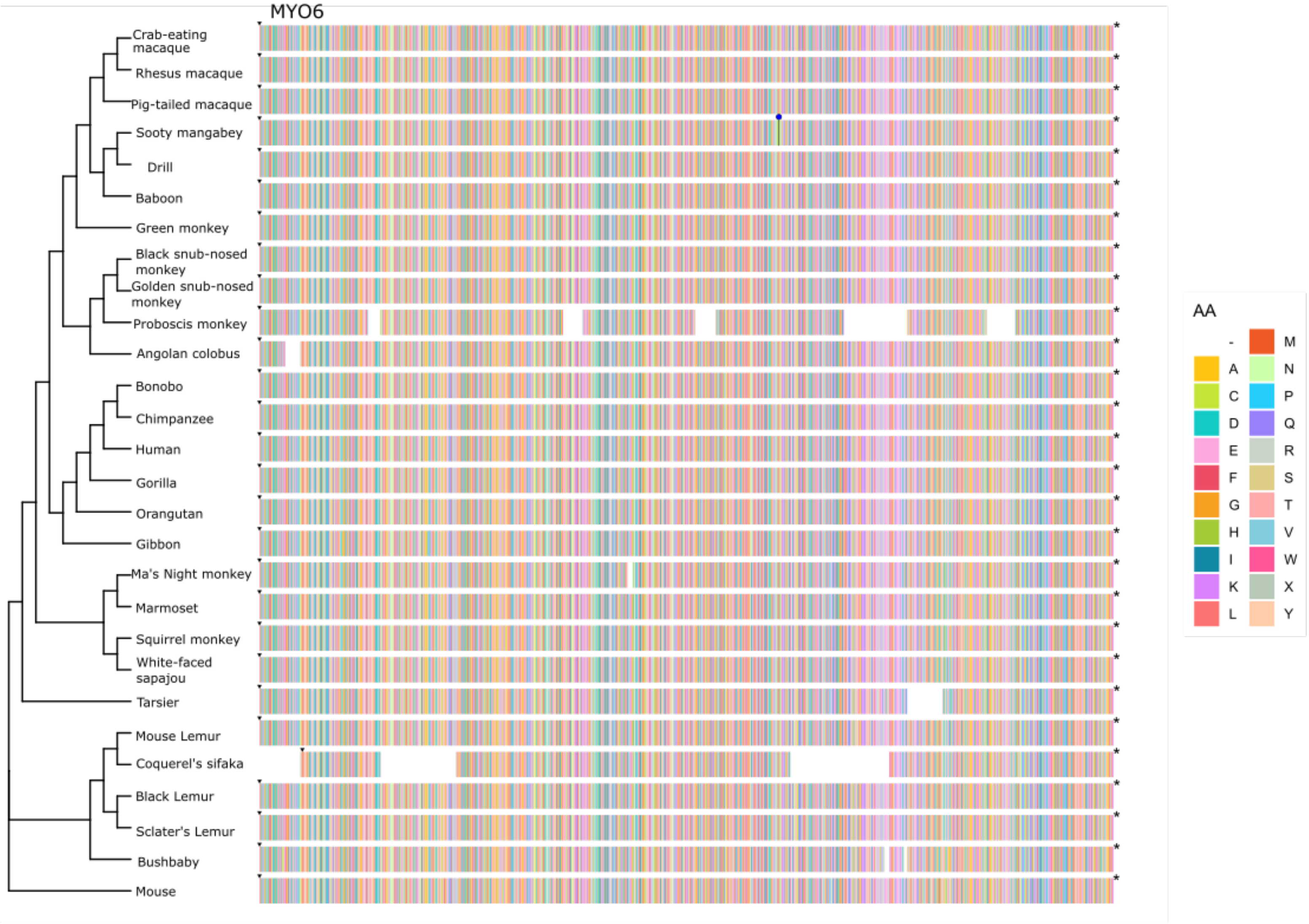

Figure S17: **PCDH15 protein alignment after filtering**. The black triangle indicates the beginning of the ORF and the asterisk the ending. The positively selected sites are surrounded by black and with a blue circle.

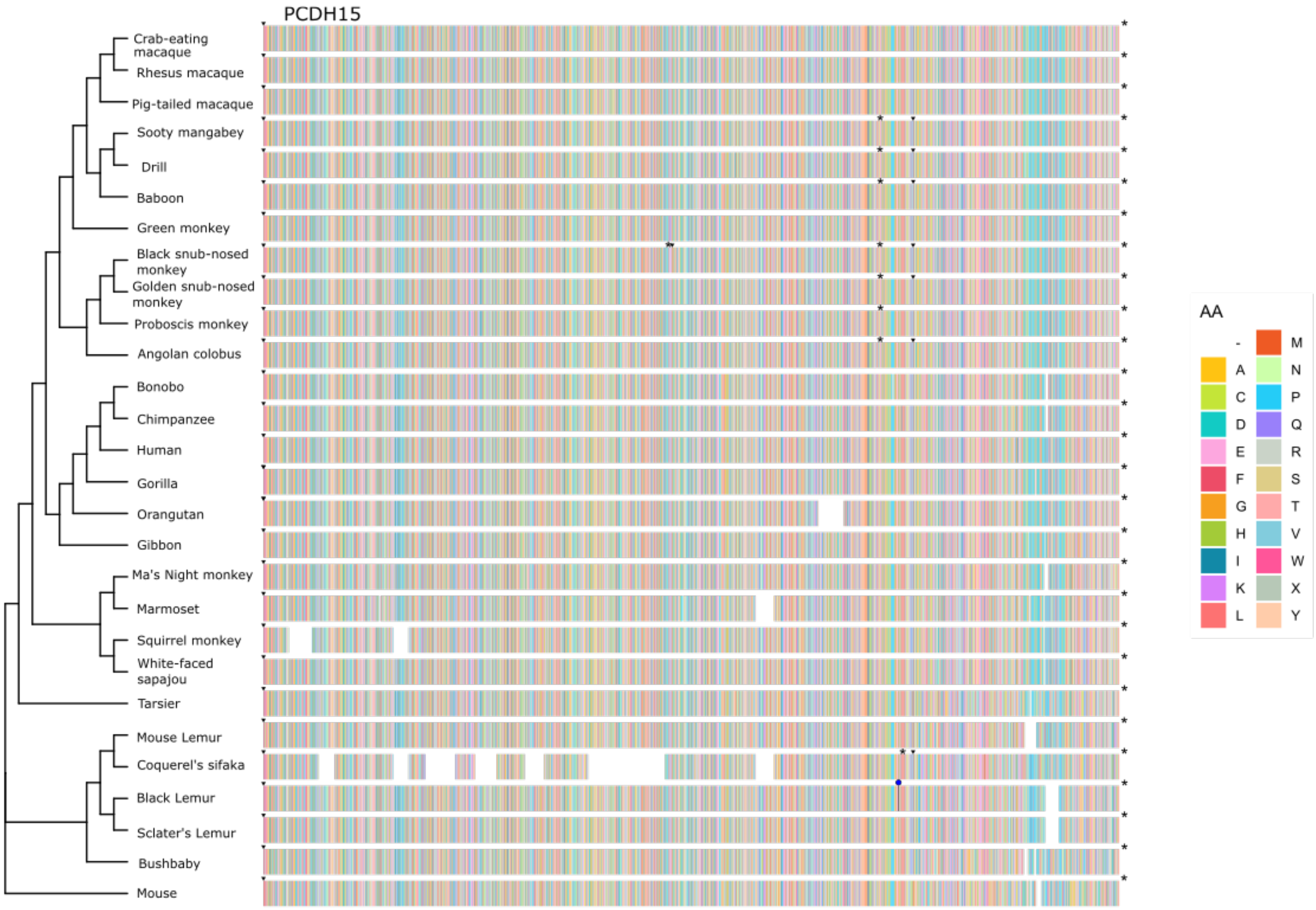

Figure S18: **PLCE1 protein alignment after filtering**. The black triangle indicates the beginning of the ORF and the asterisk the ending. The positively selected sites are surrounded by black and with a blue circle.

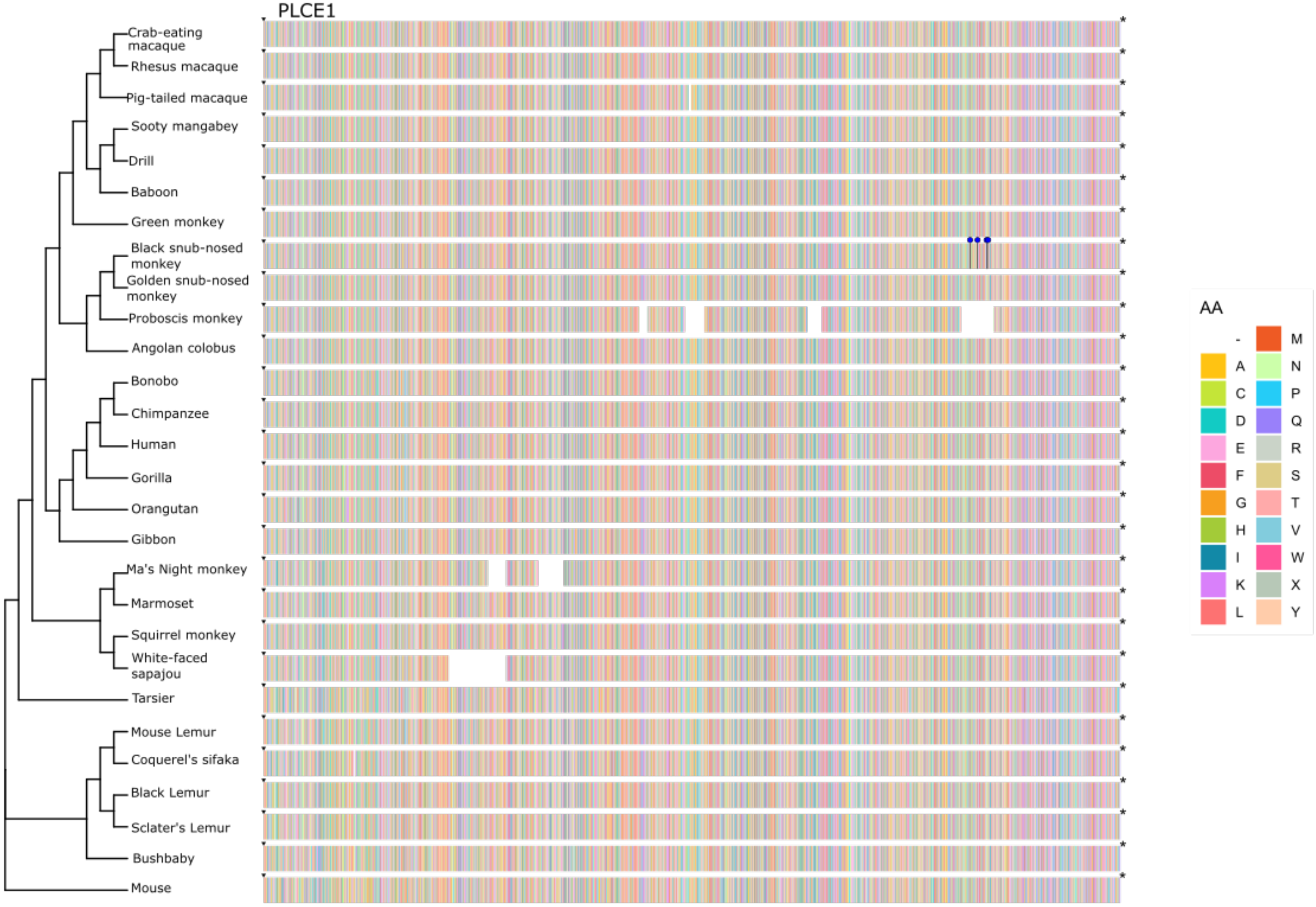

Figure S19: **PTPRQ protein alignment after filtering**. The black triangle indicates the beginning of the ORF and the asterisk the ending. The positively selected sites are surrounded by black and with a blue circle.

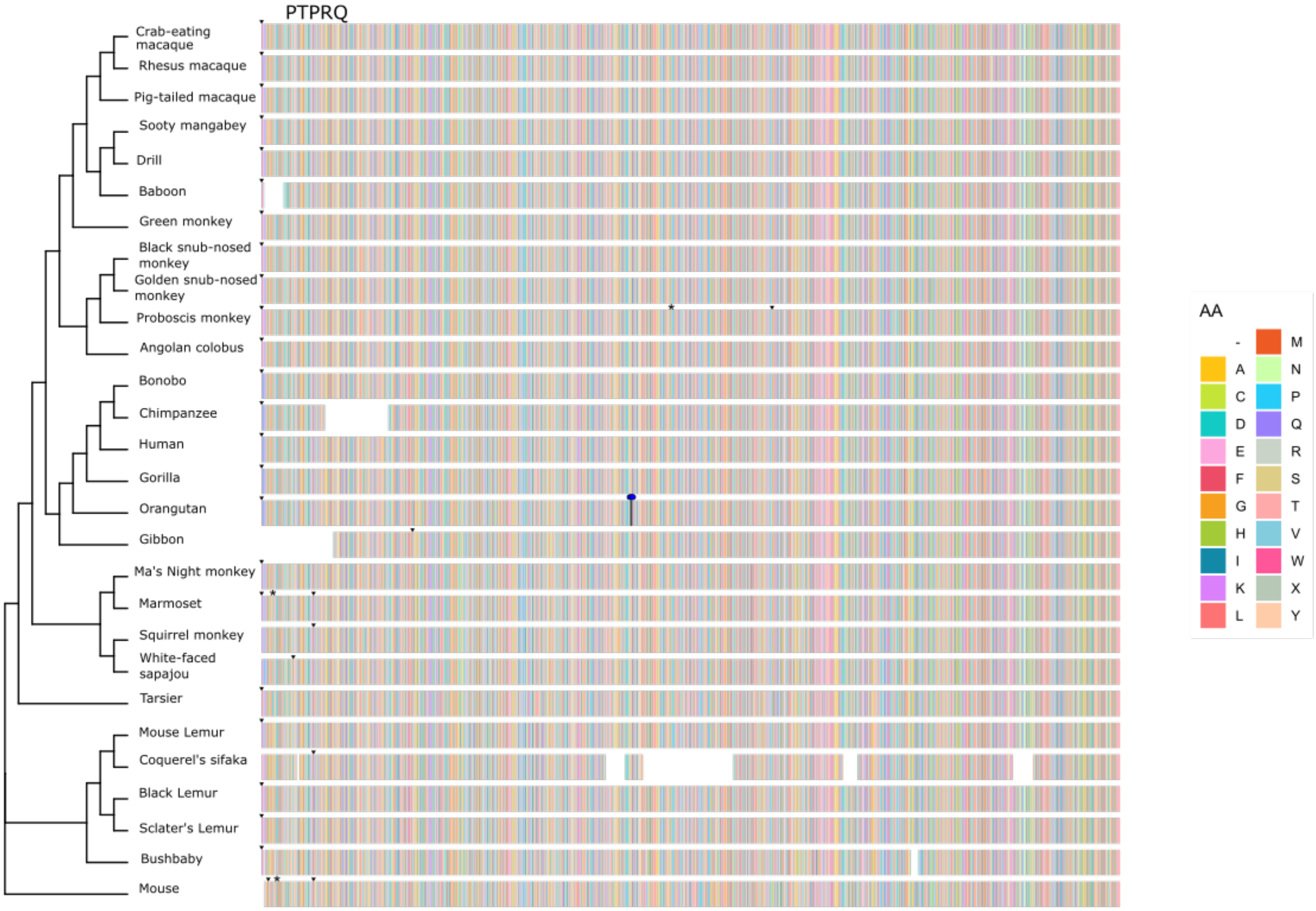

Figure S20: **RAB3IP protein alignment after filtering**. The black triangle indicates the beginning of the ORF and the asterisk the ending. The positively selected sites are surrounded by black and with a blue circle.

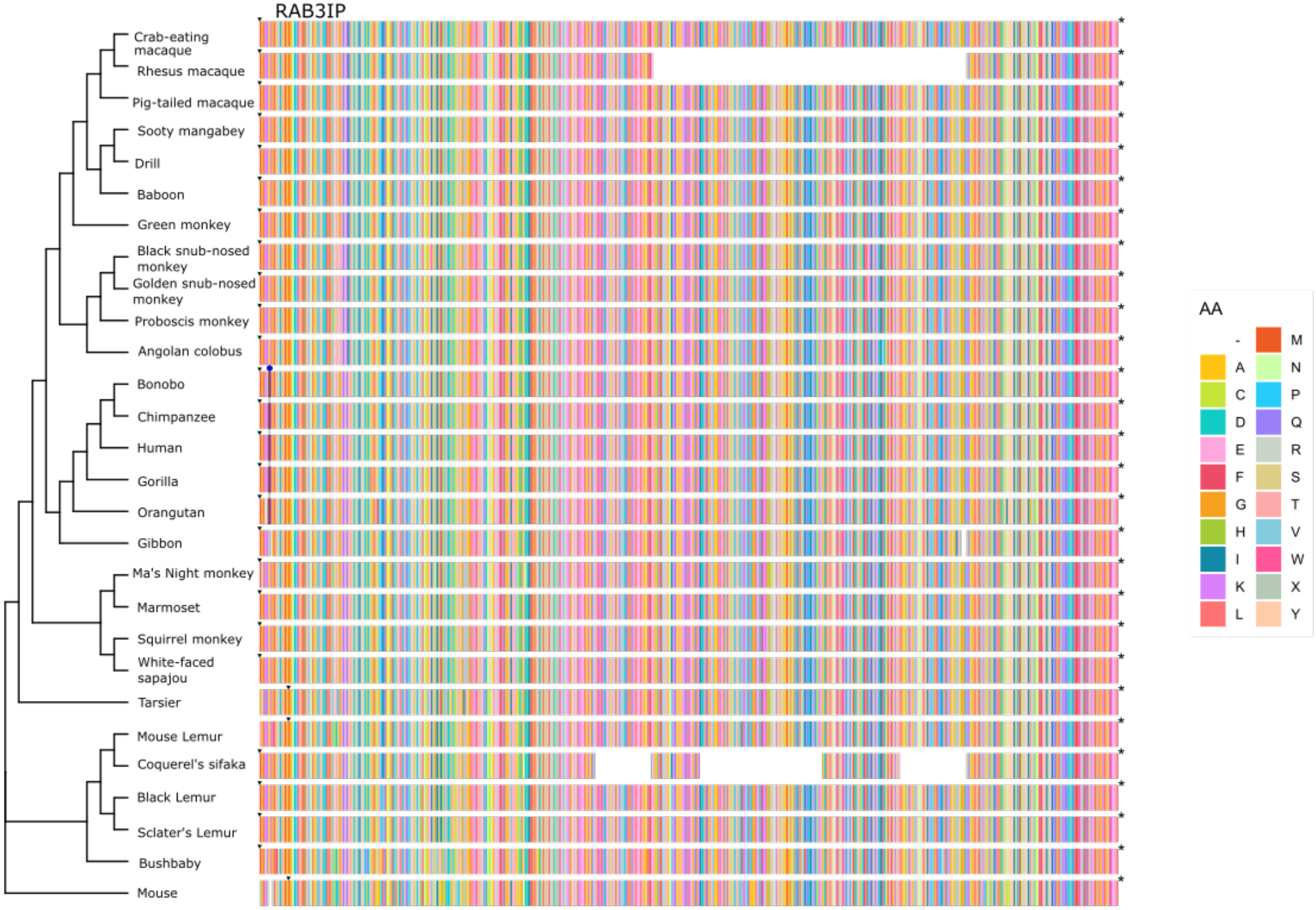

Figure S21: **RPGRIP1L protein alignment after filtering**. The black triangle indicates the beginning of the ORF and the asterisk the ending.

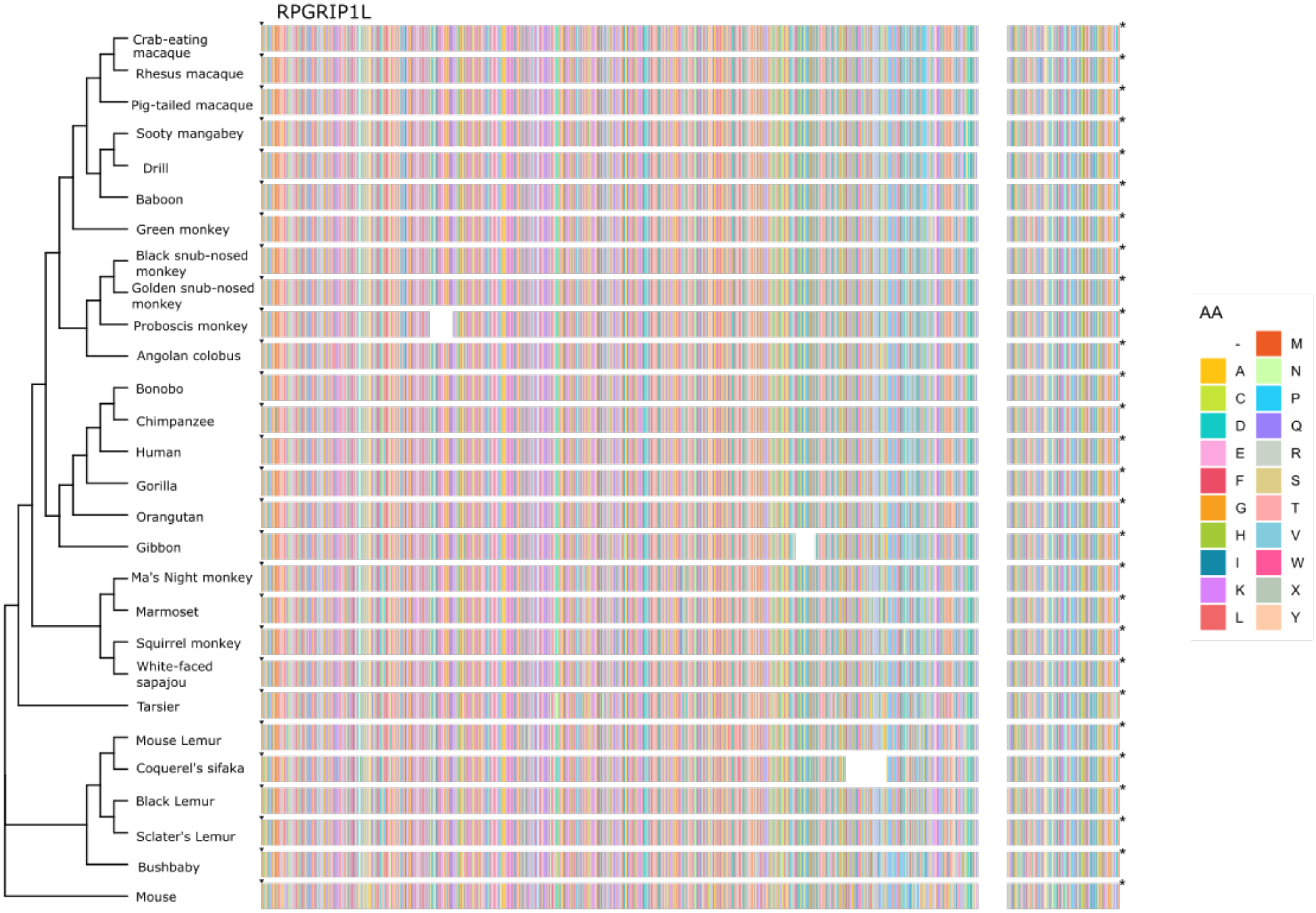

Figure S22: **SCRIB protein alignment after filtering**. The black triangle indicates the beginning of the ORF and the asterisk the ending. The positively selected sites are surrounded by black and with a blue circle.

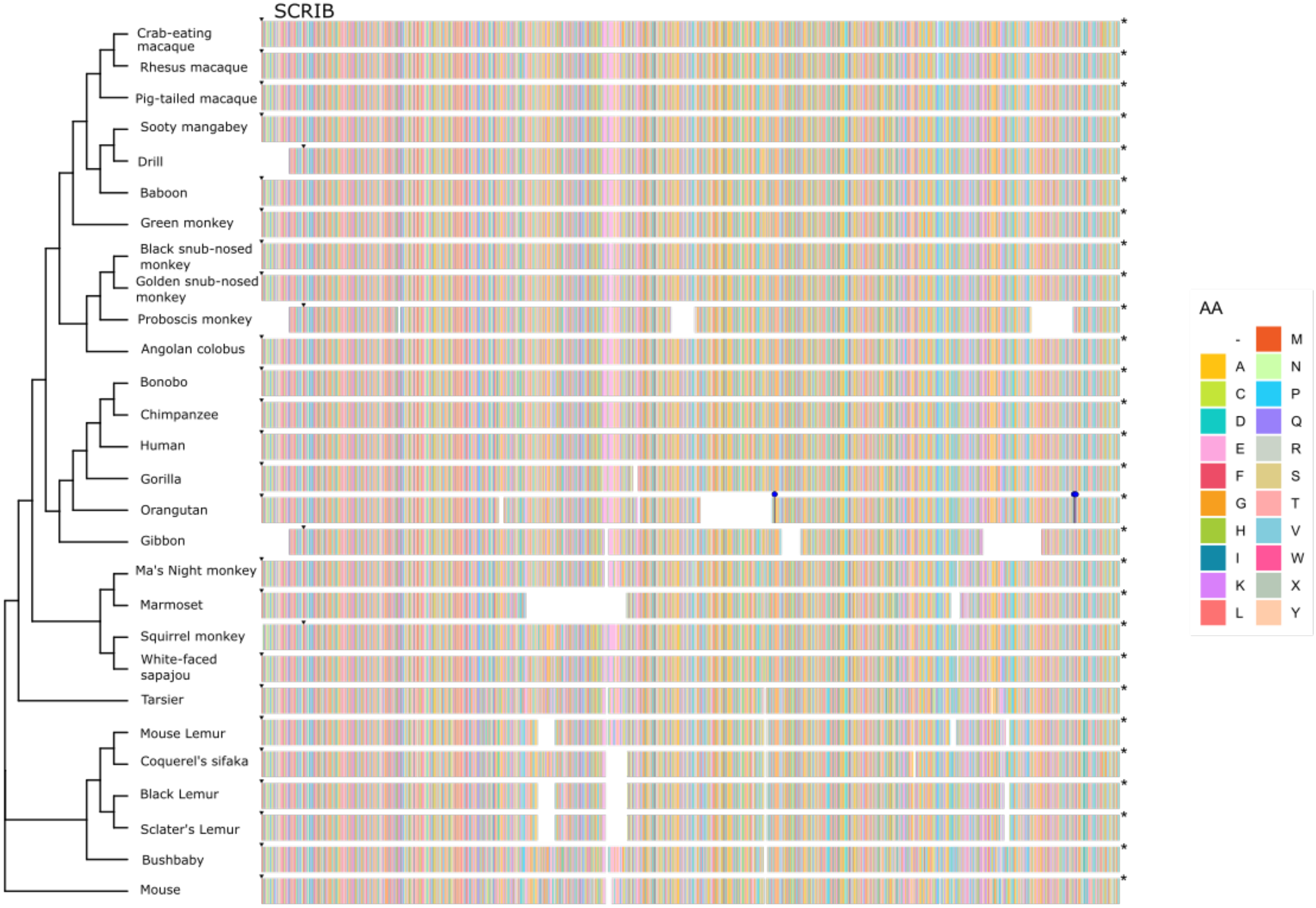

Figure S23: **SH3GL2 protein alignment after filtering**. The black triangle indicates the beginning of the ORF and the asterisk the ending. The positively selected sites are surrounded by black and with a blue circle.

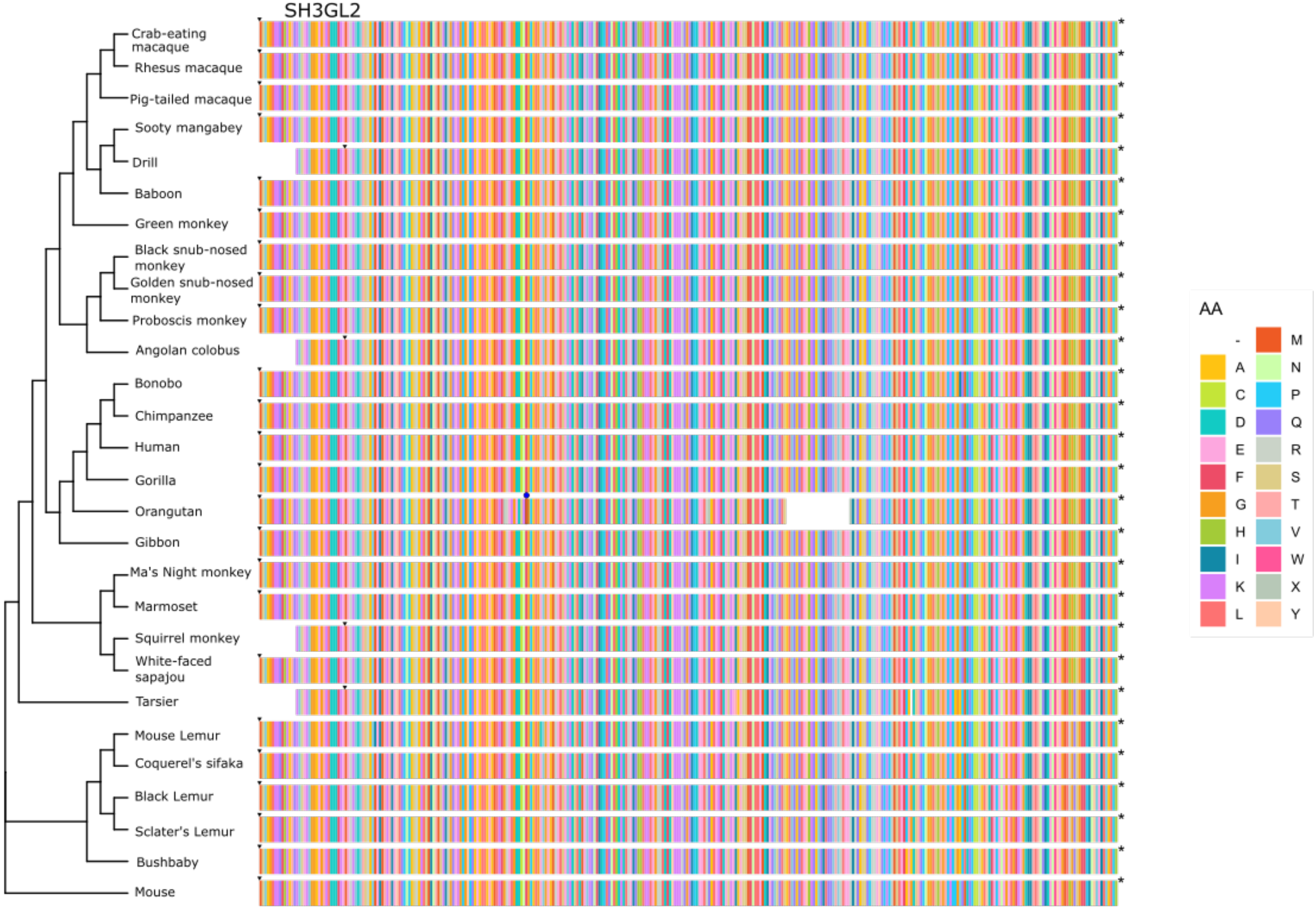

Figure S24: **SLC26A5 protein alignment after filtering**. The black triangle indicates the beginning of the ORF and the asterisk the ending. The positively selected sites are surrounded by black and with a blue circle.

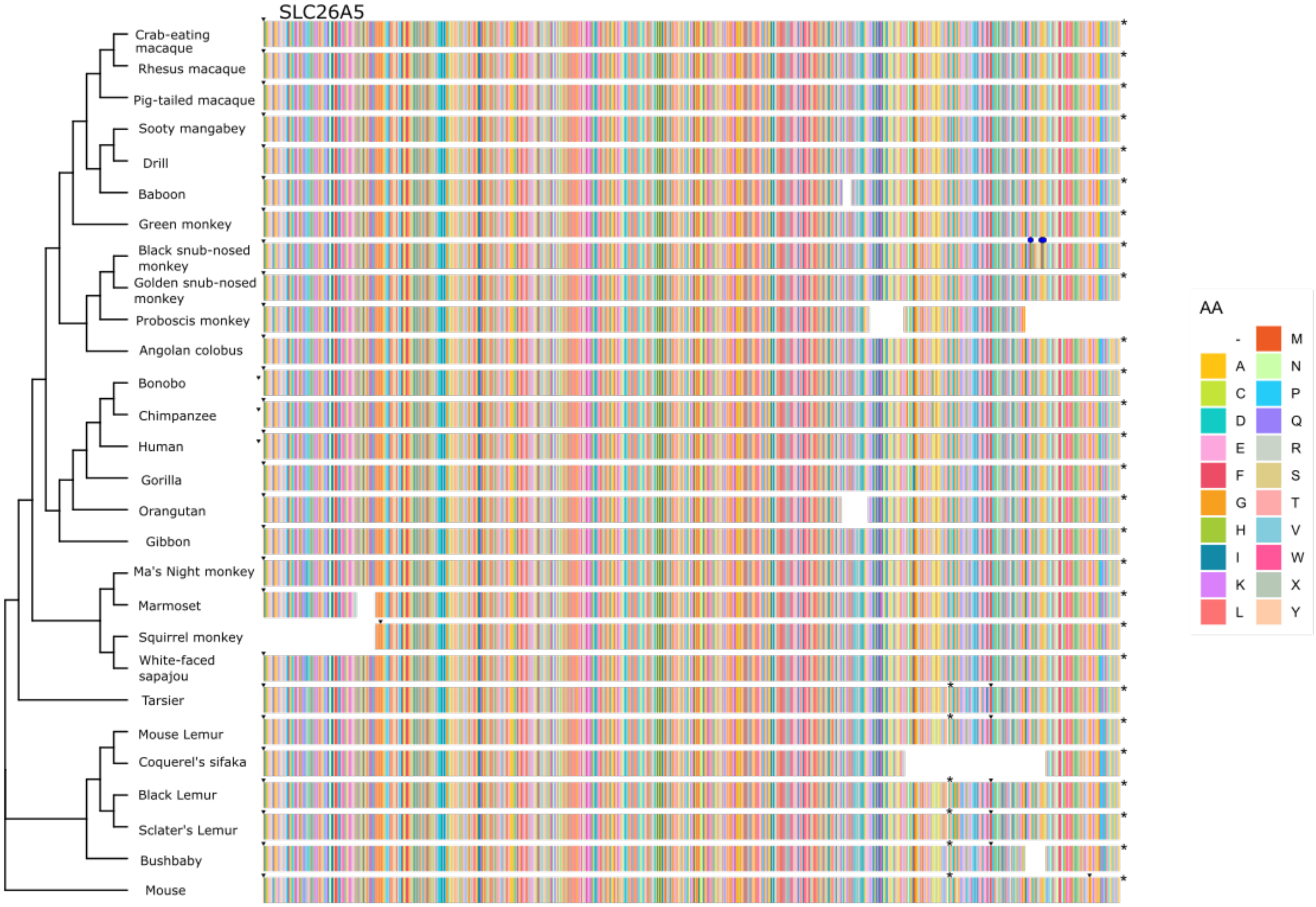

Figure S25: **SLC52A3 protein alignment after filtering**. The black triangle indicates the beginning of the ORF and the asterisk the ending. The positively selected sites are surrounded by black and with a blue circle.

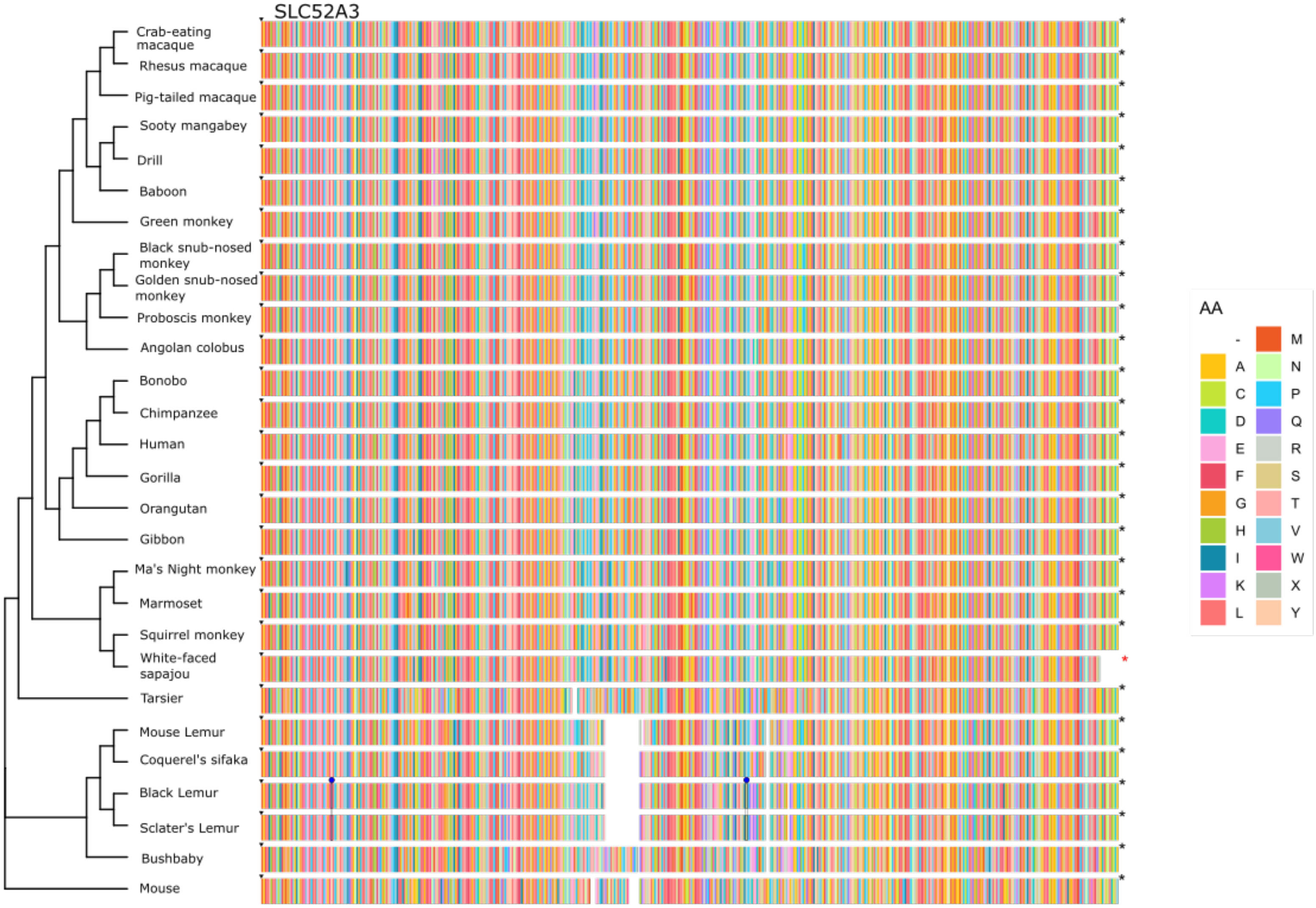

Figure S26: **SRRM4 protein alignment after filtering**. The black triangle indicates the beginning of the ORF and the asterisk the ending. The positively selected sites are surrounded by black and with a blue circle.

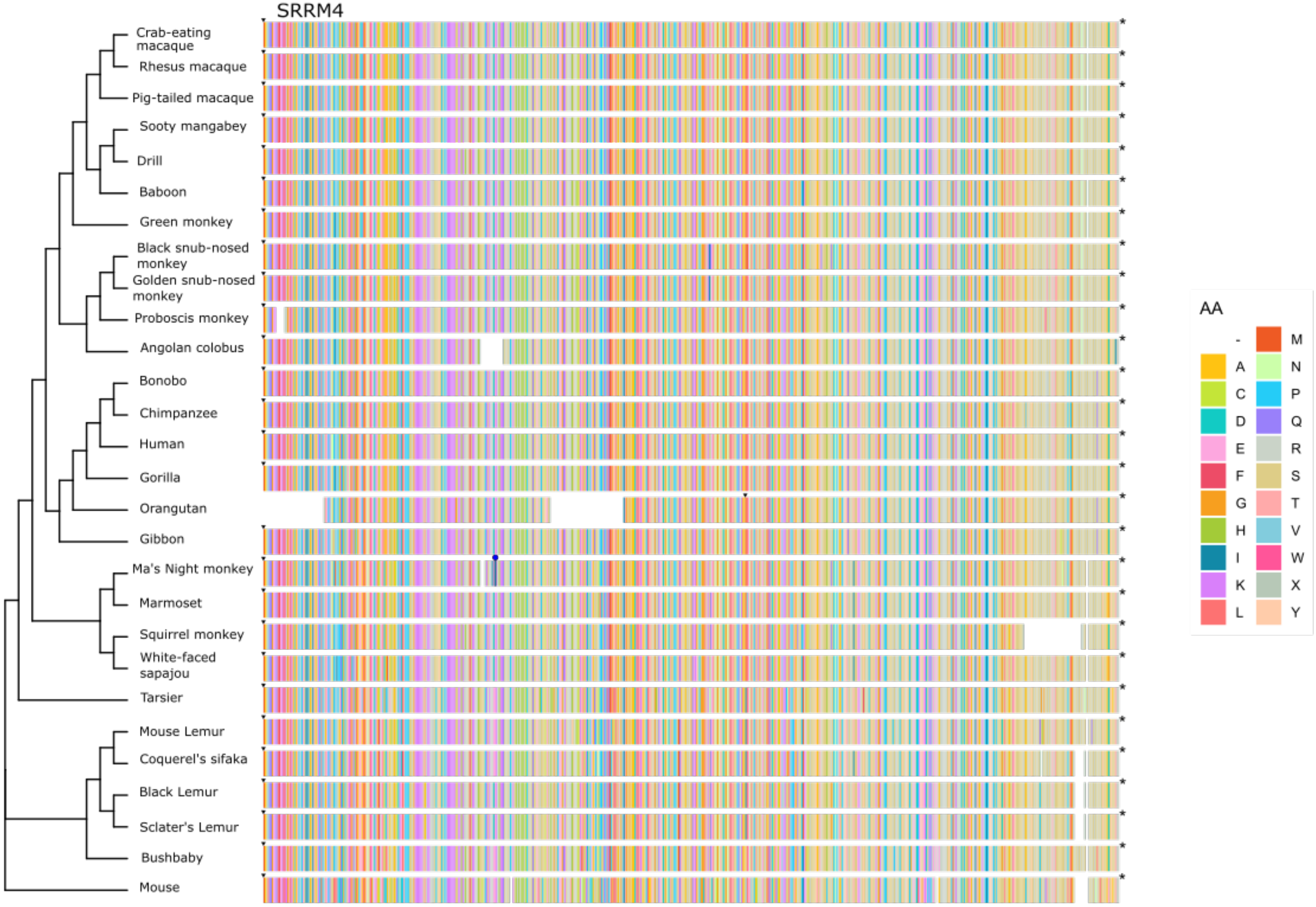

Figure S27: **STOX1 protein alignment after filtering**. The black triangle indicates the beginning of the ORF and the asterisk the ending. The positively selected sites are surrounded by black and with a blue circle.

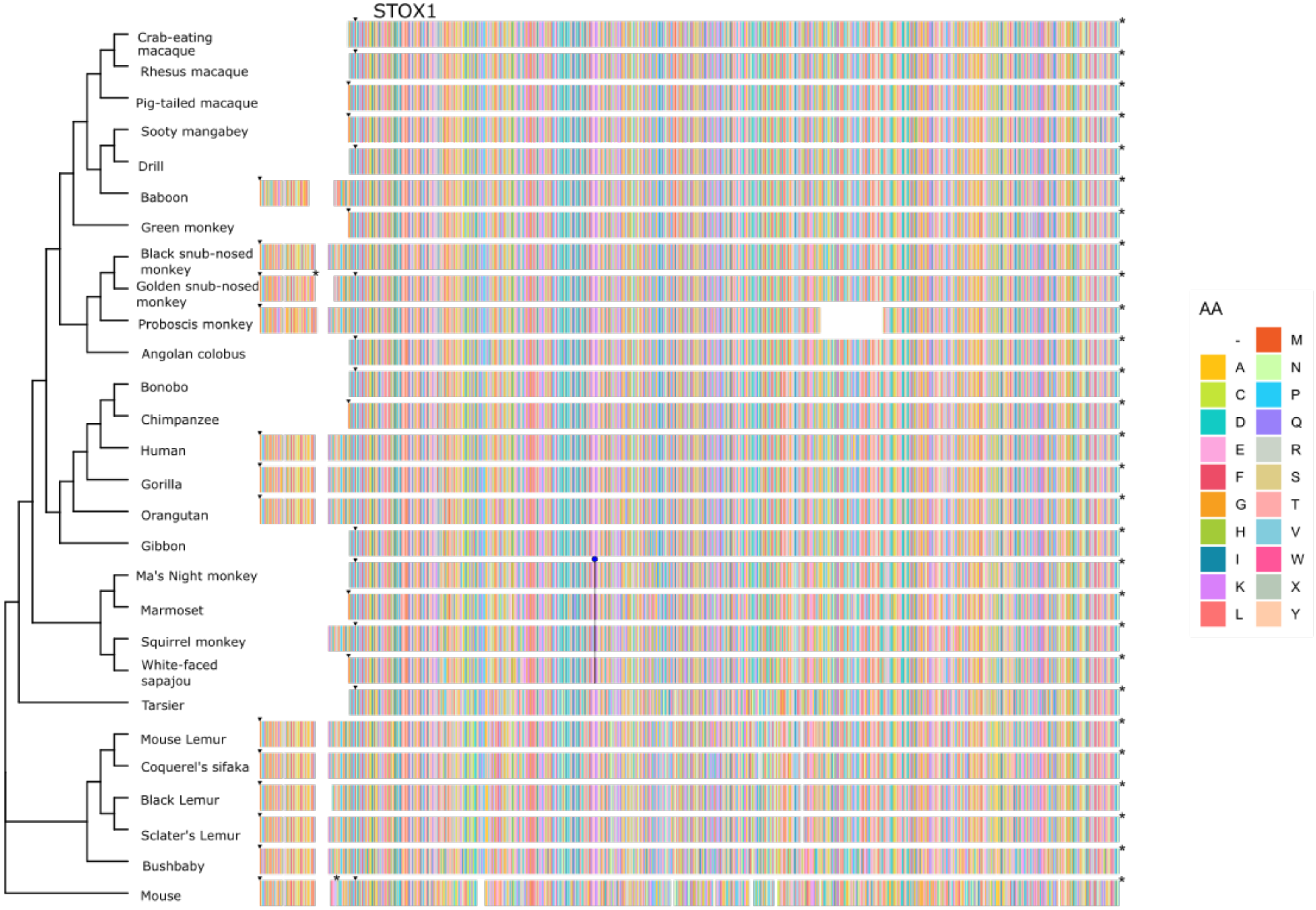

Figure S28: **STRBP protein alignment after filtering**. The black triangle indicates the beginning of the ORF and the asterisk the ending. The positively selected sites are surrounded by black and with a blue circle.

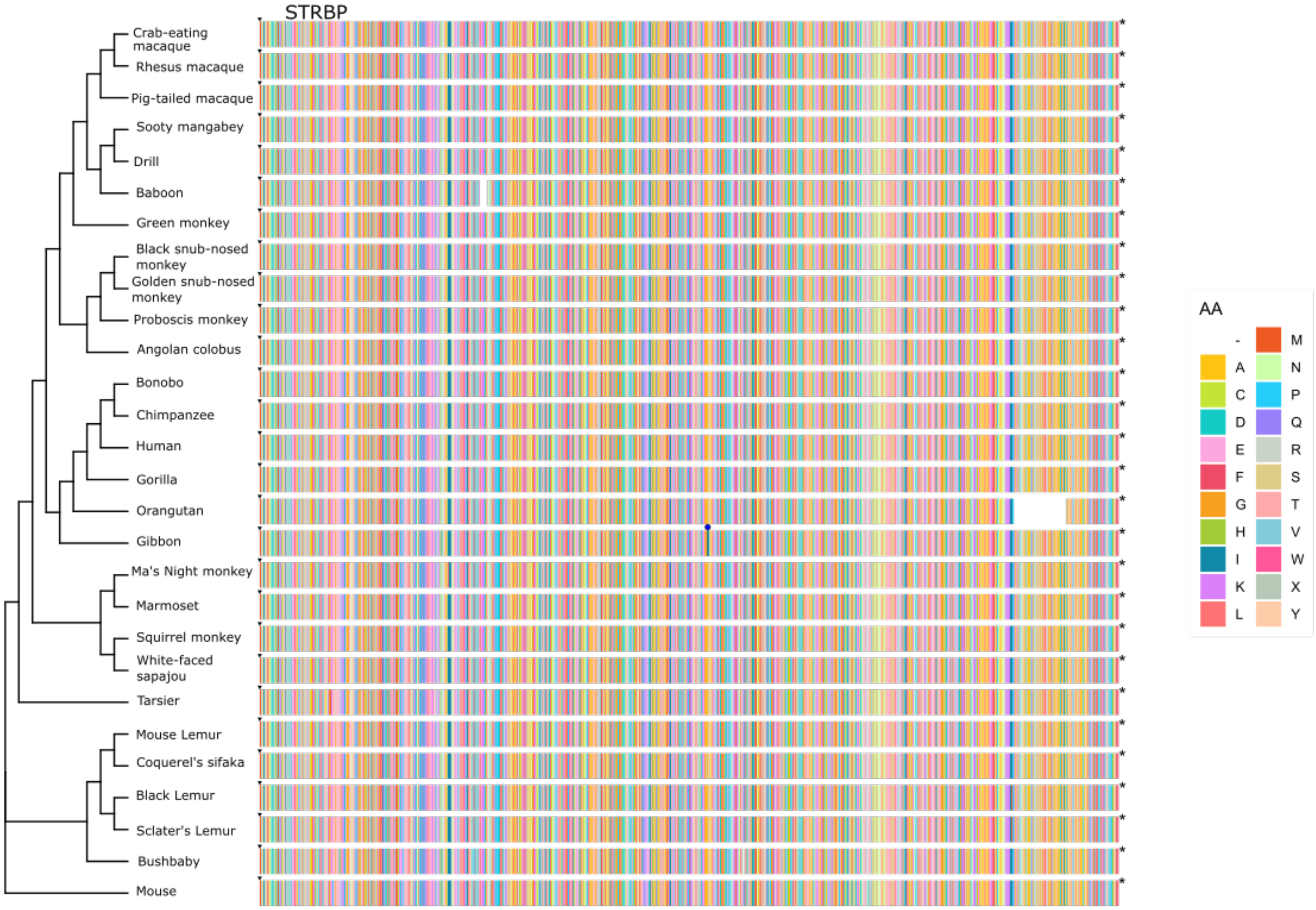

Figure S29: **USH2A protein alignment after filtering**. The black triangle indicates the beginning of the ORF and the asterisk the ending.

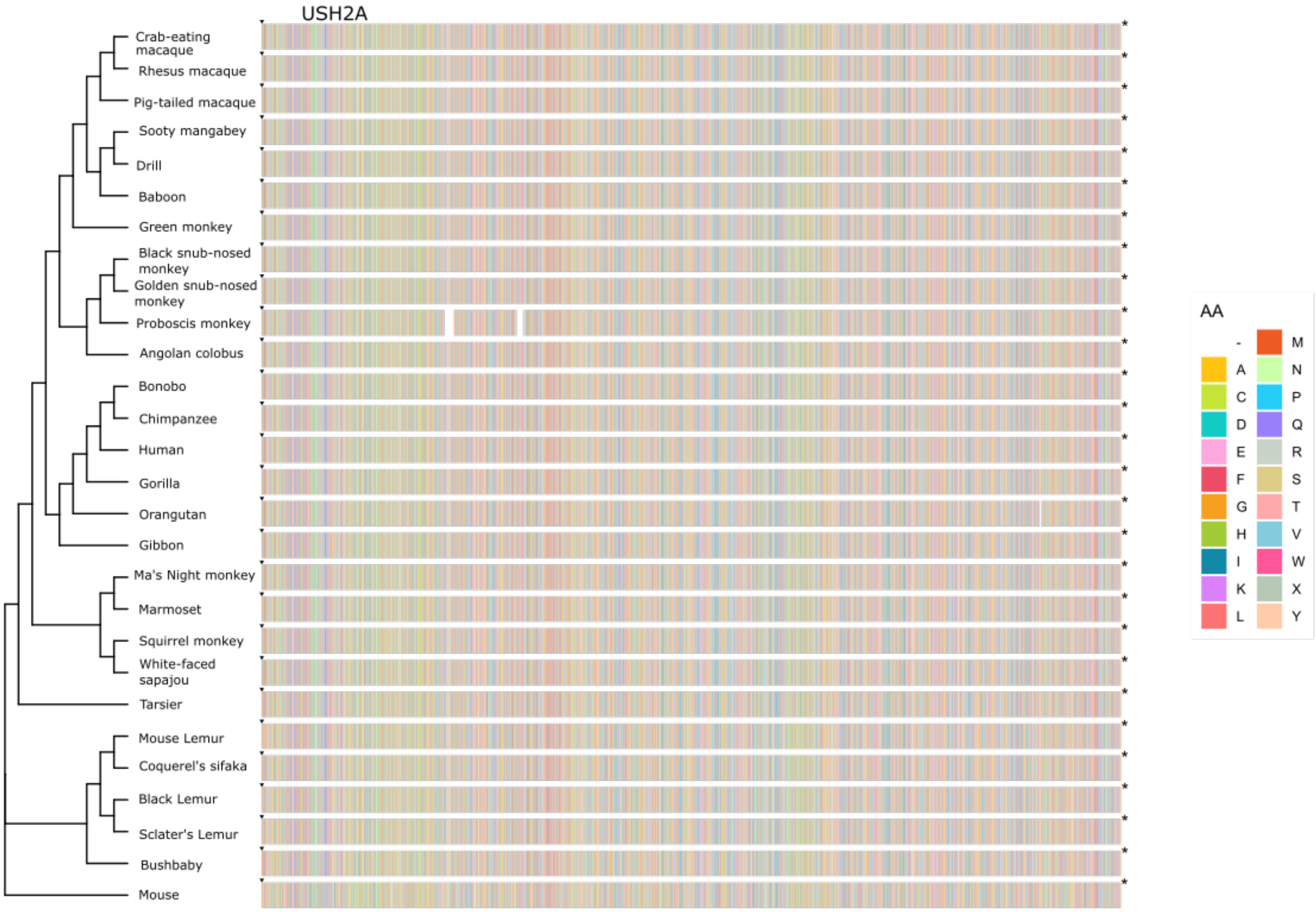

Figure S30: **WDR19 protein alignment after filtering**. The black triangle indicates the beginning of the ORF and the asterisk the ending.

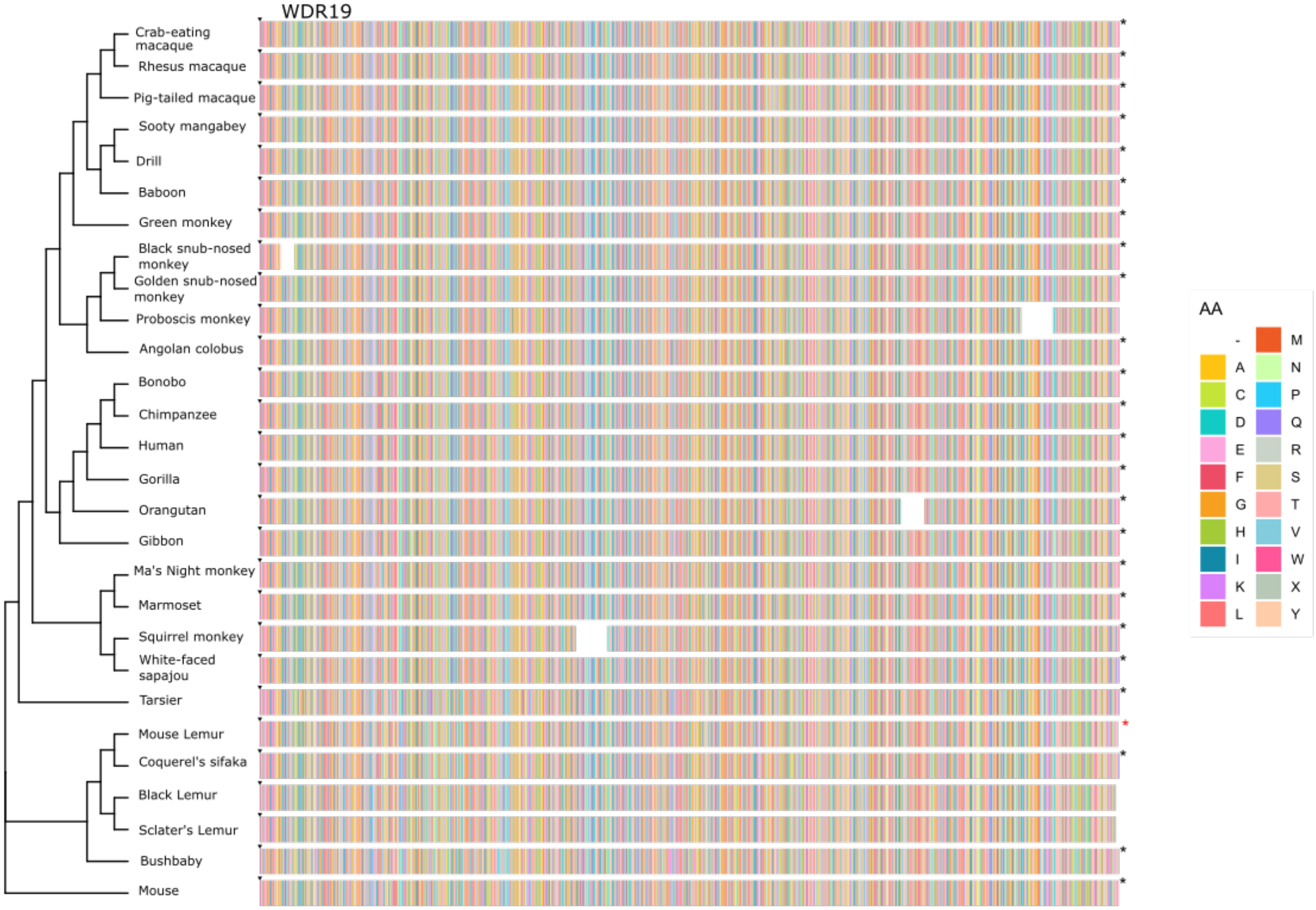

Figure S31: **WFS1 protein alignment after filtering**. The black triangle indicates the beginning of the ORF and the asterisk the ending. The positively selected sites are surrounded by black and with a blue circle.

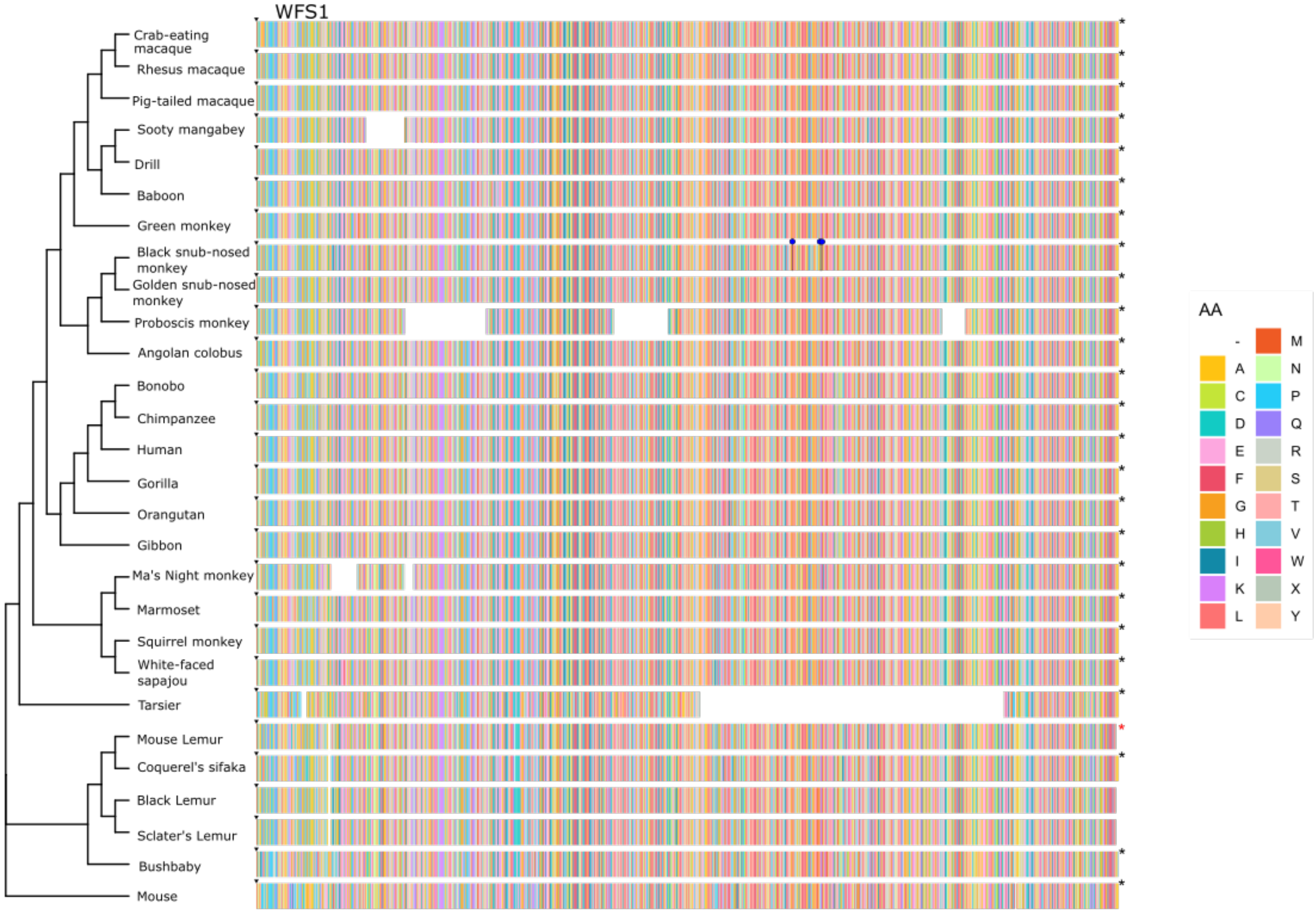

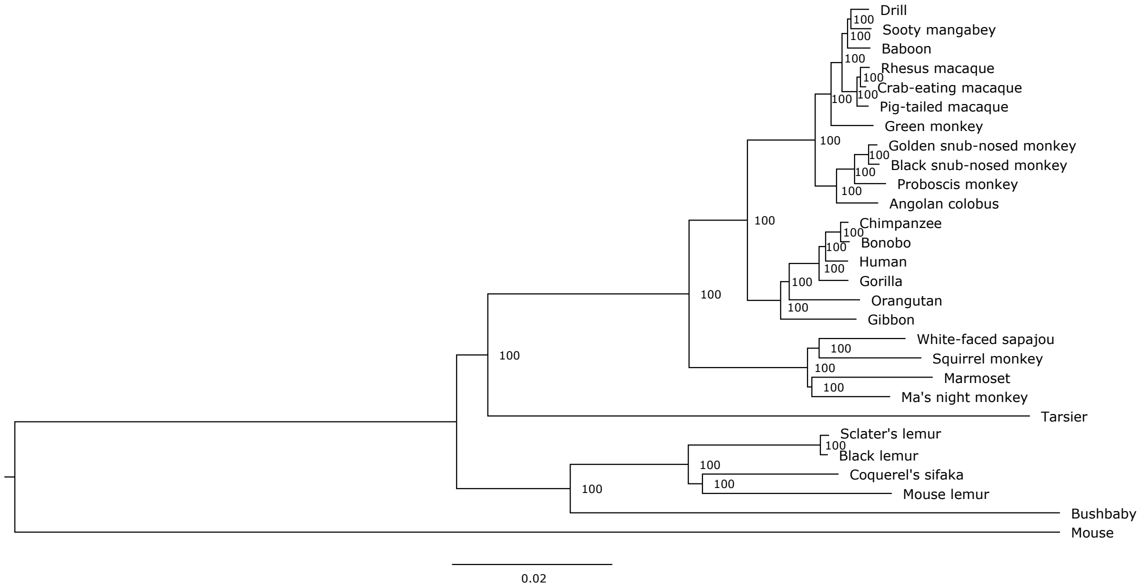

Figure S32: **Phylogenetic tree from RAxML-NG using the alignment of the 123 genes concatenated**

Table S5: **Random-site models used in site test analyses.** p is the number of free parameters in the ω distribution. M7 and M8 are referenced in Yang et al. 2000 [1] and M1a and M2a in Nielsen and Yang 1998 and Yang et al. 2005 [2, 3]

**Model**	**NSsites**	**p**	**Parameters**
M1a (neutral)	1	2	p_0 ($p_1 = 1 - p_0$), $\omega_0 < 1$, $\omega_1 = 1$
M2a (selection)	2	4	p_0, p_1, ($p_2 = 1 - p_0 - p_1$),
$\omega_0 < 1$, $\omega_1 = 1$, $\omega_2 > 1$			
M7 (beta)	7	2	p, q
M8 (beta & ω)	8	4	p_0 ($p_1 = 1 - p_0$), p, q, $\omega_s > 1$

	Gene Name	LRT M1avsM2a	LRT M7vsM8	p-value adjusted M8	BEB sites P>95%
**Site models M1a and M7 rejected**	AADACL2	8,580366	18,284264	0,000214	122Q; 315S
	CACNA1D	8,52616	25,717592	0,00002	1896I; 1918V; 1959S; 2132R
	CLCA2	8,85362	27,437232	0,00002	116W; 494H; 525M
	ECM2	6,289306	12,500792	0,00386	279G
	EPS8L2	12,595976	32,98396	0,00002	118R
	KCNJ13	6,386082	13,484298	0,00236	175T; 181M; 216K
	LMO7	7,404434	17,030682	0,0004	NA
	MYO15A	25,9459	33,315498	0,00002	1138Q
	MYO3B	7,863748	27,882002	0,00002	1138A
	NEFH	48,269298	67,92453	0,00002	369A; 685R; 690Q; 713S; 732K; 746*; 971V; 991A
	RPGRIP1L	11,17697	23,530698	0,00002	937R
	SLC13A4	10,049566	24,850782	0,00002	241Q; 443Q; 445H
	SLC52A3	14,479912	29,089864	0,00002	183R; 201G
	SOD1	11,97679	12,176496	0,00454	33W; 35S; 97D
	STOX1	13,711684	21,998824	0,000034	212R; 250M; 647S
**Site model M7 rejected**	ADGRV1	0,00209	58,912932	0,00002	3741R; 4297H
	AFTPH	0,000446	11,95472	0,005072	NA
	ATP2B2	-4E-05	20,169392	0,000084	NA
	BCL2	0	12,83259	0,00327	58H
	BMPER	0	8,997062	0,022252	280Q
	CDH23	-0,000328	52,301494	0,00002	1182I; 1997Q; 2071R; 3274R
	CLIC5	1,334706	7,398658	0,049482	18T
	COL10A1	3,100102	17,475602	0,00032	NA
	GABRB3	4,77797	8,100044	0,034844	12A; 23R
	GBX2	0	10,939802	0,008426	227A
	MYO3A	2E-06	24,657146	0,00002	NA
	MYO7A	0	14,386268	0,001504	NA
	NEK1	2,4E-05	9,07749	0,021374	NA
	NHLRC2	0,010416	14,710484	0,001278	NA
	NT5DC1	0	7,623698	0,044216	343T
	OTOF	-0,000358	8,977714	0,022468	1252R; 1313A
	RIPOR2	0	11,682308	0,00002	444T, 824V
	SALL1	2E-06	7,638062	0,0439	652A
	SHROOM2	0	30,210634	0,00002	NA
	SRRM4	0	20,616022	0,000066	55A
	STRC	0,50742	14,347528	0,001534	NA
	TMC1	1,568964	17,966318	0,000252	102I; 710V
	TWF2	0	8,097422	0,03489	NA
	USH2A	1E-05	73,104636	0,00002	1267A; 3802E; 4759G
	WFS1	0	23,072368	0,00002	57A; 80T
	ZFHX4	0,001206	17,373788	0,000338	1142I; 1312V; 1355L; 1841I; 2410A; 2573T

Table S6: **PSGs revealed by site tests after HMMCleaner and p-value corrections**. Null model M1a and M7 are both rejected in the first part of the table. The second part of the table is for the genes which the test only rejected the null model M7. The sites in the table with values superior to 0.95 are detected by the BEB method to be under positive selection. NA is for no values superior to 0.95 or no sites identified by BEB method at all.

Gene	Species	Annotation class	lnL	Selection global	Selection	gBGC	rho(S)
AADACL2	Proboscis monkey	Positive selection	-7015.06	0.3910037	11.13278	0	11.13294282
ADGRV1	Baboon	Positive selection	-86872.8	0.08797259	0.4995918	0	1.270509219
ADGRV1	Black snub-nosed monkey	Relaxation of purifying selection	-87322	0.1586822	-0.8373438	0	0.639085239
ADGRV1	Bushbaby	gBGC and Relaxation of purifying selection	-90713.66	0.2307684	-0.34369009	0.714201	0.837979203
ATP8A2	Black snub-nosed monkey	null model	-14955.78	-0.21603	0	0	0
ATP8A2	Proboscis monkey	Positive selection	-15386.07	-0.1856954	1.932492	0	2.259661468
ATP8B1	Orangutan	null model	-16272.09	0.011241542	0	0	0
CLCA2	Bushbaby	null model	-13136.53	0.3562831	0	0	0
CLIC5	Ma's night monkey	Positive selection	-6716.682	0.8018374	1.825945	0	2.176504491
COCH	Sooty mangabey	Positive selection	-5991.55	-0.8733257	7.353433	0	7.358145083
COL10A1	Bushbaby	gBGC and Positive selection	-9720.146	-0.06591739	1.0731214	0.6263824	1.630733698
DNM1	Green monkey	Positive selection	-10788.54	-2.134387	1.630534	0	2.027585974
FAT4	Sooty mangabey	Positive selection	-82647.74	-0.3692666	1.273626	0	1.768471019
FSCN2	Chimpanzee	Positive selection	-8210.26	0.656658	10.54022	0	10.54049881
KCNQ4	Black snub-nosed monkey	gBGC and Positive selection	-7417.999	-0.8675667	20.3215	8.840265	20.32150003

LMO7	Bushbaby	Relaxation of purifying selection	-22905.71	0.08328961	-0.3386662	0	0.840206579
MYO15A	Proboscis monkey	Relaxation of purifying selection	-47771.21	0.4927027	-0.8870929	0	0.621187082
MYO6	Sooty mangabey	null model	-16850.47	-0.8143292	0	0	0
PCDH15	Black Lemur	null model	-27309.34	0.02677578	0	0	0
PCDH15	Tarsier	Relaxation of purifying selection	-34241.44	-0.2586542	-0.6494285	0	0.710187598
PLCE1	Black snub-nosed monkey	Positive selection	-29395.25	0.01711486	1.995449	0	2.30942075
PTPRQ	Black Lemur	null model	-28721.88	-0.3050048	0	0	0
PTPRQ	Orangutan	Positive selection	-30691.59	-0.4375524	0.4927755	0	1.266541966
RP-GRIP1L	Ma's night monkey	null model	-16504.44	-0.3196605	0	0	0
SCRIB	Orangutan	null model	-28549.59	0.7374074	0	0	0
SH3GL2	Orangutan	Positive selection	-3481.015	-0.9717309	1.352634	0	1.824329596
SLC26A5	Black snub-nosed monkey	Positive selection	-11506.15	-2.042538	2.77201	0	2.956924782
SRRM4	Ma's night monkey	null model	-7226.447	-0.2146008	0	0	0
STOX1	Baboon	null model	-18448,09	0,8327099	0	0	0

STRBP	Gibbon	null model	-6143.348	-1.91852	0	0	0
USH2A	Bushbaby	gBGC and Relaxation of purifying selection	-80154.79	0.4914433	-0.7109771	1.26736	0.686284817
USH2A	Orangutan	Relaxation of purifying selection	-85863.09	0.3591449	-0.3947223	0	0.815589067
WFS1	Black snub-nosed monkey	Positive selection	-14233.63	0.4943452	5.70052	0	5.719647795

Table S10: **Best-fit models from the RPHAST applied to the PSGs after correction of the p-value and of the alignments.** Models included: positive selection, relaxation of the purifying selection, gBGC combined with positive selection or relaxation of the purifying selection and the null model. Only the best fit class is reported here with the likelihood and the values attributed to the species tree-wide selection coefficient (Selection global), the lineage-specific selection coefficient (Selection) and the lineage-specific conversion disparity (gBGC) and the rho (S). This last is given by the formula ρ(S) =S/(1-exp[-S]). Negative lineage specific selection coefficients and $\rho < 1$ revealed the presence of purifying selection while positive lineage specific selection coefficients and $\rho > 1$. In bold the PSGs considered like PSG with RPHAST.

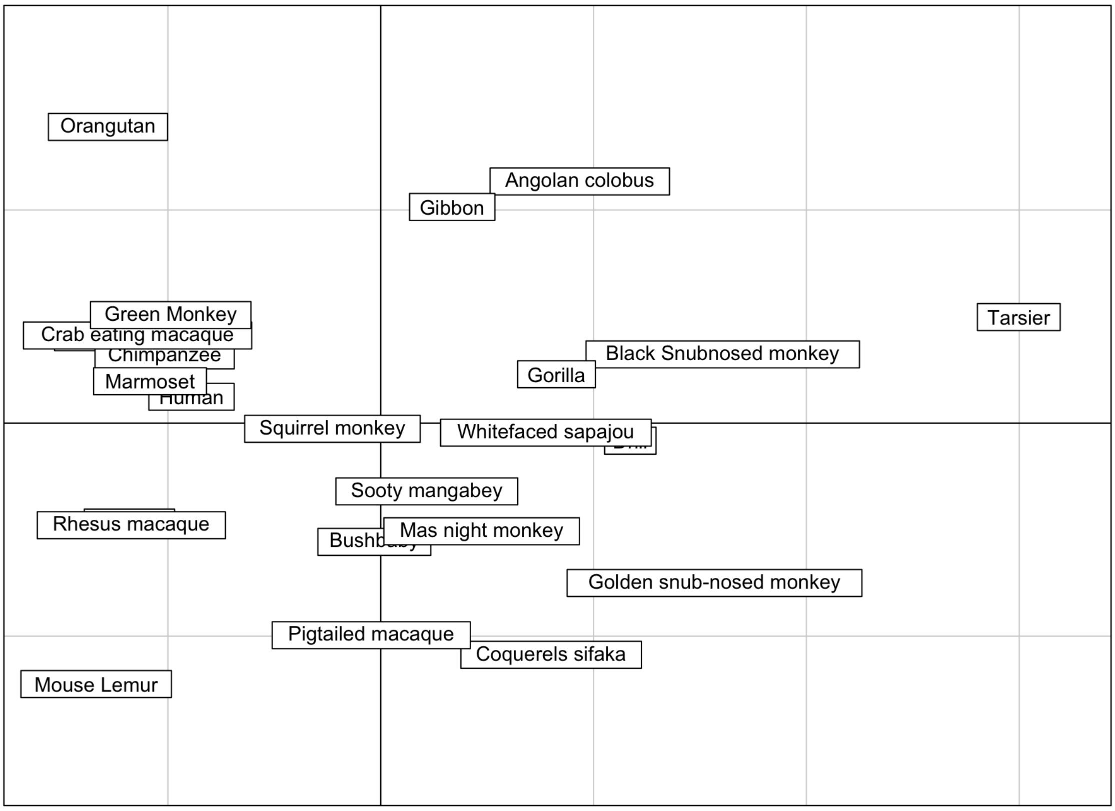

Figure S33: PCA based on whole-genome metrics calculated with QUAST-LG from the studied primates and its PSGs

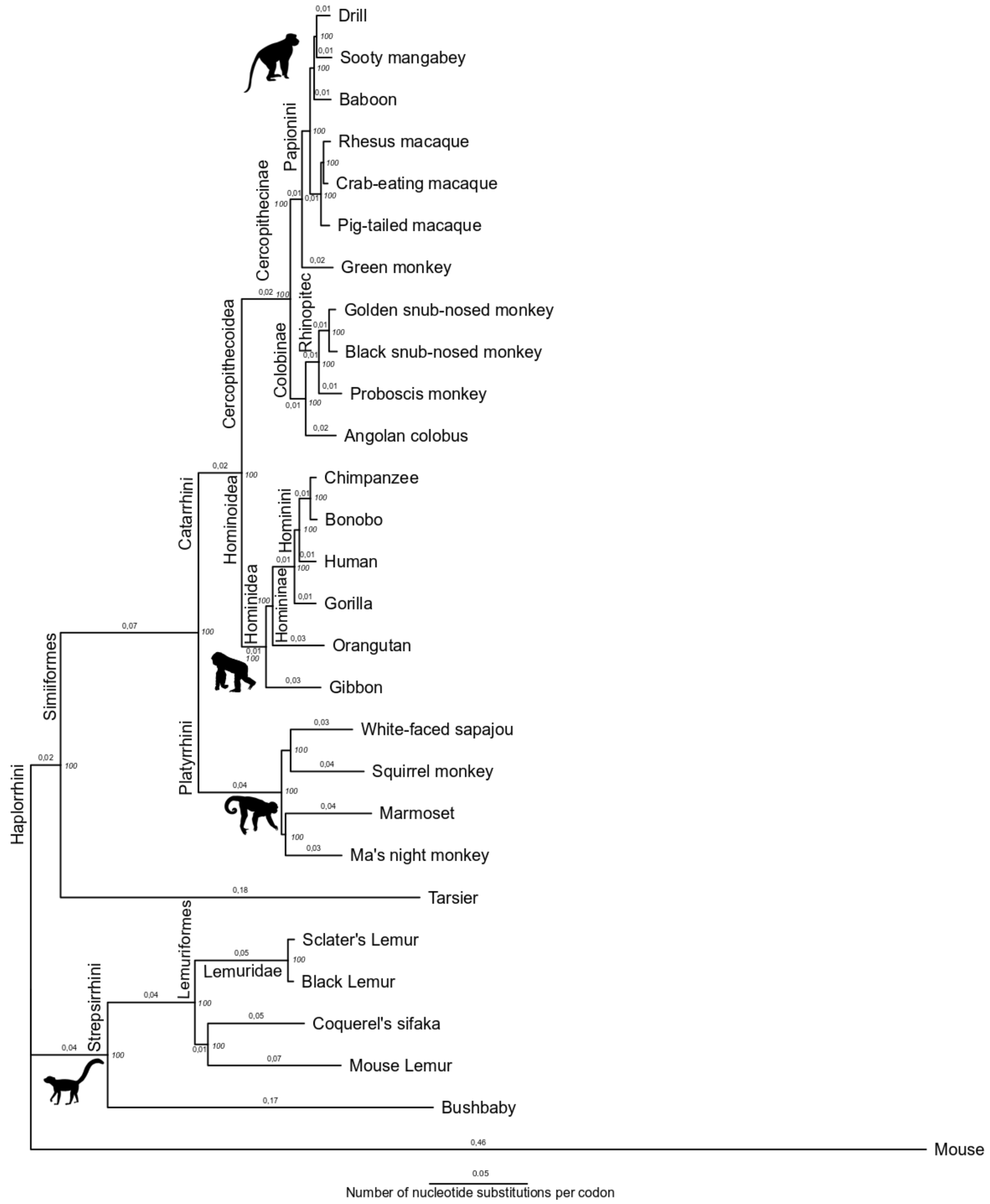

Figure S34: **Codon-based phylogenetic tree of 27 primates using the mouse species as outgroup.** Branch's lengths were estimated using the codeml M0 evolutionary model (F3X4) on the concatenated alignment using the 123 genes (106,358 codons). The length of branches reflects the number of nucleotide substitutions per codon mentioned on the tree, excepted when values are lower than 0.01. Bootstraps from phylogenetic reconstruction are highlighted in italics.

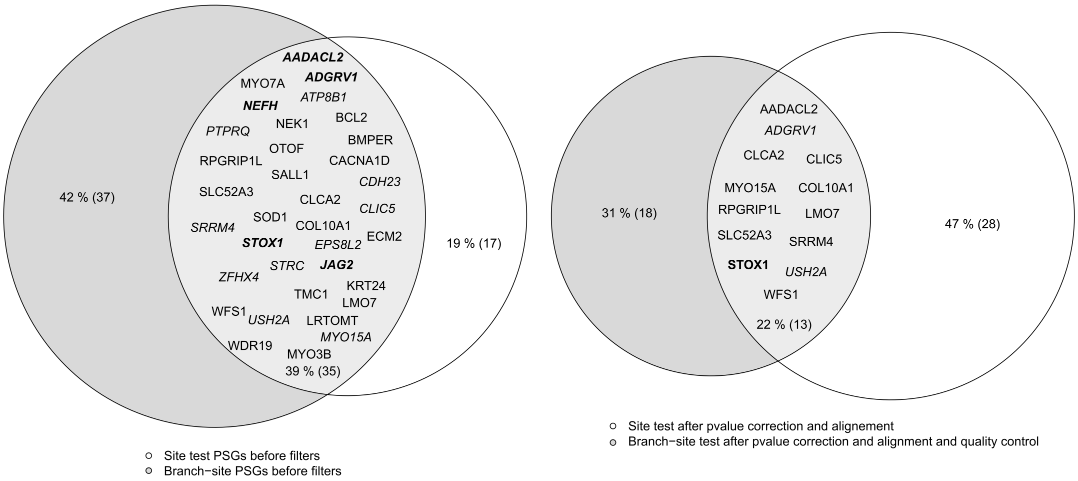

Figure S35: **Venn diagram representing shared PSGs between both site test and branch-site test analyses, before (a) and after filtering for confounding factors (b)**; in bold, are represented the Continuous PSG, in italic the Parallel PSGs and in italic bold those displaying both type of signals

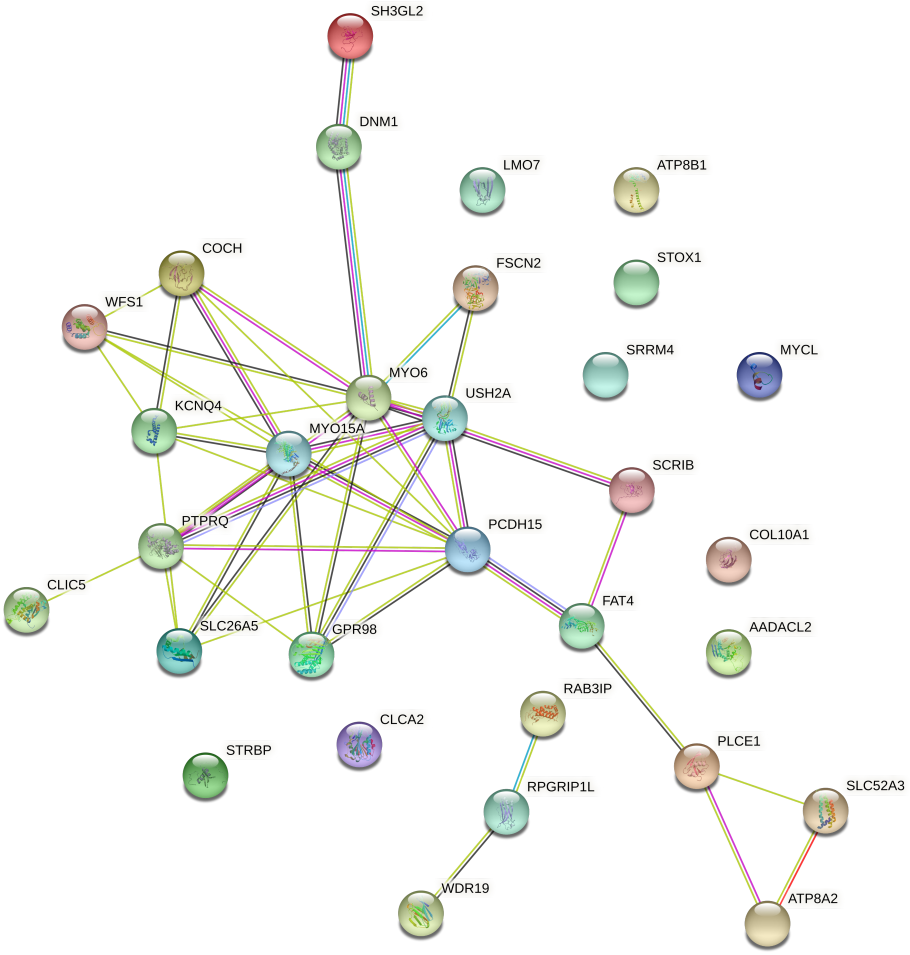

Figure S36: Network based on the 31 PSGs from the branch-site test used by STRING. GPR98 is also known as ADGRV1.

References

[1] Ziheng Yang, Rasmus Nielsen, Nick Goldman, and Anne-Mette Krabbe Pedersen. Codon-substitution models for heterogeneous selection pressure at amino acid sites. *Genetics*, 155(1):431–449, 2000.

[2] Rasmus Nielsen and Ziheng Yang. Likelihood models for detecting positively selected amino acid sites and applications to the hiv-1 envelope gene. *Genetics*, 148(3):929–936, 1998.

[3] Ziheng Yang, Wendy SW Wong, and Rasmus Nielsen. Bayes empirical bayes inference of amino acid sites under positive selection. *Molecular biology and evolution*, 22(4):1107–1118, 2005.
